# Rates of Physician Coprescribing of Opioids and Benzodiazepines After the Release of the Centers for Disease Control and Prevention Guidelines in 2016

**DOI:** 10.1001/jamanetworkopen.2019.8325

**Published:** 2019-08-02

**Authors:** Molly M. Jeffery, W. Michael Hooten, Anupam B. Jena, Joseph S. Ross, Nilay D. Shah, Pinar Karaca-Mandic

**Affiliations:** 1 Division of Health Care Policy Research, Mayo Clinic, Rochester, Minnesota; 2 Department of Anesthesiology and Perioperative Medicine, Mayo Clinic, Rochester, Minnesota; 3 Department of Health Care Policy, Harvard Medical School, Boston, Massachusetts; 4 Department of Medicine, Massachusetts General Hospital, Boston; 5 National Bureau of Economic Research, Cambridge, Massachusetts; 6 Section of General Internal Medicine, Department of Medicine, Yale University School of Medicine, New Haven, Connecticut; 7 Department of Health Policy and Management, Yale University School of Public Health, New Haven, Connecticut; 8 Center for Outcomes Research and Evaluation, Yale-New Haven Hospital, New Haven, Connecticut; 9 Robert D. and Patricia E. Kern Center for the Science of Health Care Delivery, Mayo Clinic, Rochester, Minnesota; 10 OptumLabs, Cambridge, Massachusetts; 11 Department of Finance, Carlson School of Management, University of Minnesota, Minneapolis

## Abstract

**Importance:**

The Centers for Disease Control and Prevention guidelines in 2016 recommended avoiding concurrent use of opioids and benzodiazepines.

**Objective:**

To determine whether the release of the guidelines was associated with changes in coprescription of opioids and benzodiazepines.

**Design, Setting, and Participants:**

This retrospective cohort study used claims data obtained from a US national database of medical and pharmacy claims for 3 598 322 adult commercially insured patients and 1 299 142 Medicare Advantage (MA) beneficiaries with no recent history of cancer, sickle cell disease, or hospice care who ever used prescribed opioids during the study period, January 1, 2014, through March 31, 2018.

**Exposures:**

Overlapping opioid and benzodiazepine prescriptions filled.

**Main Outcomes and Measures:**

The extent (proportion of person-months with any overlapping days of prescription of opioids and benzodiazepines) and intensity (proportion of days with opioids prescribed where benzodiazepines were also available) of coprescription.

**Results:**

Of 4 897 464 patients (with 13.4 million person-months of opioid use), the total number of unique commercially insured individuals was 3 598 322 (1 974 731 women [54.9%]), and the total number of unique MA beneficiaries was 1 299 142 (770 256 women [59.3%]). Among 128 576 participants experiencing chronic pain episodes, more than one-half of person-months of long-term opioid use occurred in women (52.7% of person-months among those with commercial insurance and 62.4% of person-months among MA beneficiaries). The median (interquartile range) age of the participants was 51 (41-58) years for patients in the commercial insurance group and 70 (61-77) years for those in the MA group. The mean (SE) extent of coprescription was 23.0% (0.18%) for the commercial insurance group and 25.7% (0.18%) for the MA group. The extent of coprescription decreased in the targeted guideline population—individuals with long-term opioid use—after the guideline release (postguideline slope, −0.95 percentages point per year [95% CI, −1.44 to −0.46 percentage points per year] for the commercial insurance group and −1.06 percentage points per year [95% CI, −1.49 to −0.63 percentage points per year] for the MA group). Nontargeted short-term episodes of opioid use were associated with no change or small declines in trend (for the MA group, postguideline slope of 0.47 percentage point per year [95% CI, 0.35-0.59 percentage point per year]; for the commercial insurance group, postguideline slope of −0.05 percentage point per year [95% CI, −0.12 to 0.02 percentage point per year]). High coprescribing intensity was seen, with 79.3% (95% CI, 78.9%-79.6%) of opioid prescription days in the commercial insurance group and 83.9% (95% CI, 83.7%-84.2%) in the MA group overlapping with benzodiazepines. There was no change in the intensity of coprescribing. Intensity of coprescription was higher when the same clinician prescribed opioids and benzodiazepines.

**Conclusion and Relevance:**

This study observed a reduction in the extent but not intensity of coprescribing of benzodiazepines for patients with long-term opioid use.

## Key Points

**Question:**

Was the release of the Centers for Disease Control and Prevention’s 2016 opioid prescribing guidelines associated with changes in the rate of coprescription of opioids and benzodiazepines?

**Findings:**

This cohort study of administrative data for 4 897 464 patients found small, statistically significant decreases in the rate of coprescription in the 2 years after the guideline release. These decreases were seen only in the targeted guideline population—people using opioids long term—not in those receiving short courses of opioids.

**Meaning:**

Coprescribing rates of opioids and benzodiazepines may have decreased after the Centers for Disease Control and Prevention guideline release, but associations with patient outcomes are not yet known.

## Teaser

This cohort study evaluates whether the 2016 release of the Centers for Disease Control and Prevention (CDC) guidelines for opioid prescribing was associated with changes in the rates of opioid and benzodiazepine coprescription among commercially insured patients and US Medicare Advantage beneficiaries.

## Introduction

Rates of opioid prescribing and deaths attributed to prescription opioid overdose have quadrupled in the United States over the past 20 years.^1^ One-quarter^2^ to one-half^3^ of opioid-related deaths also involve a benzodiazepine, which is consistent with the known risks of adverse events when these 2 drug classes are used concomitantly.^4,5,6,7^ In particular, the risk of overdose increases when people take opioids and benzodiazepines at the same time.^3,4,8^ Coprescribing of opioids and benzodiazepines in the United States has previously been shown to be common.^8^ One study^9^ found that, in 2013, 17% of prescription opioid users also used a benzodiazepine. Recognizing the risk of coprescribing, in 2016 the US Food and Drug Administration^10^ issued a warning to patients and clinicians about the potential risks of combined opioid and benzodiazepine use.

In March 2016, the US Centers for Disease Control and Prevention (CDC) released guidelines^11^ intended to help primary care clinicians treat patients with chronic non–cancer-related pain. These guidelines specifically included a category A recommendation to avoid coprescription of opioids and benzodiazepines “whenever possible.”^11^ The panel writing the guidelines noted that there may be clinically appropriate situations where the drugs may be used together, giving the example of a person taking a stable low dose of a benzodiazepine who experiences acute pain. However, the overall recommendation in the context of long-term opioid use was to avoid benzodiazepines if possible, given patient needs.

To better understand the association of the CDC opioid guidelines with coprescribing, changes in extent of coprescribing of benzodiazepines among patients newly initiating long-term opioid therapy were compared before and after release of the guidelines in a large population of commercially insured patients and Medicare Advantage (MA) beneficiaries. The primary outcomes were the extent and intensity of coprescription of opioids and benzodiazepines. Because of the well-recognized racial differences in opioid prescribing practices,^12,13,14,15,16^ changes in coprescribing by race and ethnicity were also analyzed.

## Methods

### Study Population

Episodes of opioid use between January 1, 2014, and March 31, 2018, were identified in the OptumLabs Data Warehouse,^17^ which includes deidentified claims data for privately insured participants and MA enrollees in a large, private, US health plan. Participants were stratified into 2 insurance groups, commercially insured and MA beneficiaries. Prescriptions for all opioids, including tramadol, filled through the health plan during the study period were captured (eAppendix 1 and eTable 1 in the Supplement).^18^

An episode of opioid use was defined as a period when the patient went no more than 30 days without using opioids. Opioids were considered to be available to a beneficiary from the prescription fill date to the time the prescription would be expected to be completed if the medication was consumed according to prescriber instructions. Episodes of opioid use that spanned at least 90 calendar days and included at least 120 days’ supply or 10 fills (ie, how many times a participant received medication from the pharmacy) were classified as long term.^19^ Episodes of opioid use not meeting these criteria were classified as short term and were used as a comparison group to estimate trends unrelated to the release of the CDC guidelines.

Inclusion criteria for incident episodes of opioid use were (1) the first opioid prescription filled in an episode was preceded by 90 days of insurance enrollment with no opioids available, and (2) the participant continued insurance enrollment for at least 120 days after the first prescription fill in the episode. The latter criterion was necessary to ascertain whether an episode represented a long-term episode of opioid use. Exclusion criteria included (1) episodes of opioid use with 1 or more prescriptions written for more than 1000 morphine milligram equivalents (MMEs) per day, (2) individuals younger than 18 years, (3) individuals with cancer, (4) individuals with sickle cell disease, (5) individuals receiving hospice services, and (6) episodes of opioid use that included buprenorphine, methadone, or naloxone, potentially suggesting treatment for opioid use disorder. A flowchart is provided in eFigure 1 in the Supplement.

This study was deemed exempt from review by the University of Minnesota and Mayo Clinic institutional review boards because data were existing and deidentified. This report follows the Reporting of Studies Conducted Using Observational Routinely Collected Health Data (RECORD) reporting guidelines for observational studies using routinely collected health data.^20^

### Outcomes

The primary outcomes were the extent and intensity of coprescription of opioids and benzodiazepines. The extent of coprescription was measured with a flag to identify each calendar month during which a patient had overlapping opioid and benzodiazepine prescriptions filled. The intensity of coprescription was measured as the proportion of opioid prescription days where benzodiazepines were also available. In a sensitivity analysis focusing on long-term episodes starting before and after the guideline release, the outcome variable was an indicator of whether there was any benzodiazepine prescription filled in the first 90 days of the opioid use episode.

### Covariates

Beneficiary covariates included age, sex, race/ethnicity, state of residence, and indicator variables for each of the Elixhauser comorbidities^21^ measured on a rolling 6-month basis. Coprescribing by the same physician (as opposed to 1 physician prescribing the benzodiazepine and another prescribing the opioid) was assessed for person-months with 1 or more days of overlap. The categorical variable could take the following values: (1) at least 1 day of overlap with opioid and benzodiazepine prescriptions written by different physicians, (2) at least 1 day of overlap with prescriptions written by the same physician, or (3) at least 1 day of overlap, but it was not possible to assess whether the same physician prescribed the drugs because of unknown prescriber identification numbers on 1 or both of the prescriptions.

### Statistical Analysis

Changes in coprescribing were analyzed both as a 1-time change in level at the time of CDC guideline release and as a change in trend after the guideline release. To implement this approach, piecewise regression with a single knot spline was used, with the knot placed at the release of the guidelines (March 2016).

The unit of observation was a person-month during which the beneficiary had opioids available for at least 1 day. Generalized linear models were specified with clustered SEs to account for repeated observations on the same patients.

The Holm^22^ sequentially rejective multiple test procedure (2-sided) was used to control the familywise error rate at 0.05. The tables, text, and figures denote whether comparisons are statistically significant after this adjustment. Summary statistics are presented as percentage (SE) for categorical variables and as quartiles (median, 25th percentile, and 75th percentile) for continuous variables.

Details on model specifications and secondary analyses are provided in eAppendixes 2, 3, 4, 5, and 6 in the Supplement. Figures present adjusted rates of overlapping opioid and benzodiazepine prescriptions filled and adjusted proportions of opioid prescription days where benzodiazepines were also available, with 95% confidence intervals shown in the tables. All analyses were conducted with Stata/MP statistical software version 15.0 (StataCorp).^23^

## Results

The study sample included 13.4 million person-months of opioid use contributed by 4 897 464 beneficiaries. In both the commercial and MA populations, non-Hispanic white patients accounted for a larger proportion of long-term compared with short-term opioid use episode person-months (Table 1). The total number of unique commercial beneficiaries was 3 598 322 (1 974 731 women [54.9%]), and the total number of unique MA beneficiaries was 1 299 142 (770 256 women [59.3%]). Among 128 576 beneficiaries experiencing chronic pain episodes, more than one-half of person-months of long-term opioid use occurred in women (52.7% of person-months among those with commercial insurance and 62.4% of person-months among MA beneficiaries). The median (interquartile range) age was 51 (41-58) years for those with commercial insurance and 70 (61-77) years for MA beneficiaries. The median age was older for patients with long-term compared with short-term opioid use episodes in the commercial population; the opposite was true in the MA population.

**Table 1.** Characteristics of Study Cohort^a^

Characteristic	Commercial Insurance		Medicare Advantage	
	Long-term Opioid Episode	Short-term Opioid Episode	Long-term Opioid Episode	Short-term Opioid Episode
Categorical variables^b^				
Female^b^	52.7 (0.25)	55.7 (0.03)	62.4 (0.22)	61.9 (0.06)
Race/ethnicity^b^				
Non-Hispanic white	77.9 (0.20)	71.5 (0.03)	70.5 (0.21)	67.0 (0.06)
Non-Hispanic black	10.2 (0.15)	10.7 (0.02)	17.1 (0.17)	17.0 (0.05)
Hispanic	7.96 (0.13)	11.0 (0.02)	7.88 (0.12)	9.65 (0.04)
Non-Hispanic Asian	1.14 (0.05)	3.0 (0.01)	1.09 (0.04)	1.85 (0.02)
Unknown or other	2.82 (0.08)	3.7 (0.01)	3.50 (0.08)	4.59 (0.02)
Selected comorbidities^c^				
Hypertension without complications	19.7 (0.14)	9.94 (0.02)	42.2 (0.17)	38.6 (0.04)
Diabetes without complications	7.03 (0.10)	4.04 (0.01)	17.50 (0.14)	16.4 (0.04)
Chronic pulmonary disease	4.48 (0.07)	2.37 (0.01)	14.1 (0.12)	10.0 (0.03)
Depression	7.39 (0.09)	3.21 (0.01)	11.6 (0.11)	6.19 (0.02)
Diabetes with complications	3.57 (0.07)	1.64 (0.01)	11.0 (0.11)	9.56 (0.03)
Concurrent opioid and benzodiazepines prescriptions, mo^b^	23.00 (0.18)	8.99 (0.02)	25.70 (0.18)	14.00 (0.04)
Proportion of opioid prescription days overlapping with benzodiazepines^b^				
All person-months	18.7 (0.17)	9.27 (0.03)	22.0 (0.18)	13.6 (0.05)
Person-months with any overlap days	79.3 (0.16)	78.5 (0.06)	83.9 (0.13)	83.4 (0.06)
Person-months, No.	168 810	583 020	228 195	454 771
Same physician prescribing opioids and benzodiazepines (of person-months with any overlap)^b^				
Yes	59.1 (0.12)	51.8 (0.07)	60.3 (0.10)	50.4 (0.07)
No	37.9 (0.12)	39.5 (0.06)	30.0 (0.10)	39.3 (0.07)
Unknown	2.99 (0.04)	8.8 (0.04)	9.7 (0.06)	10.4 (0.05)
Person-months, No.	168 810	583 020	228 195	454 771
Opioid dose per opioid use day, morphine milligram equivalents				
<20	28.37 (0.19)	18.94 (0.02)	39.52 (0.19)	33.87 (0.05)
20 to <50	49.04 (0.20)	59.11 (0.03)	42.52 (0.19)	53.01 (0.05)
50 to <90	13.17 (0.12)	18.36 (0.02)	10.04 (0.11)	10.60 (0.02)
90 to <120	3.70 (0.06)	2.36 (0.01)	2.95 (0.06)	1.59 (0.01)
≥120	5.73 (0.10)	1.23 (0.01)	4.97 (0.10)	0.94 (0.01)
Continuous variables				
Age, median (interquartile range), y	51 (41-58)	46 (34-56)	70 (61-77)	72 (67-78)
Opioid use, median (interquartile range), mo				
Opioid prescription days	28 (19-30)	5 (3-8)	28 (20-30)	7 (4-15)
Morphine milligram equivalents opioids	630 (340-1200)	150 (80-240)	520 (300-990)	160 (90-300)
Observations, No.				
Person-months	734 545	6 485 891	889 459	3 255 263
Unique beneficiaries^d^	59 423	3 564 678	69 153	1 262 727

^a^ Except where noted otherwise, data are percentage of person-months (SE).

^b^ The SEs for proportions were clustered on beneficiary identification to account for multiple observations of the same beneficiaries.

^c^ Elixhauser comorbidities were calculated on a rolling 6-month basis using *International Classification of Diseases, Ninth Revision* and *Tenth Revision* codes from Quan et al.^21^

^d^ Some beneficiaries had both short-term and long-term opioid episodes while observed. Each is counted as a unique beneficiary in both categories of opioid use. The total number of unique commercial beneficiaries was 3 598 322; the total number of unique Medicare beneficiaries was 1 299 142. A small number of people appeared both as commercially insured and Medicare beneficiaries at different times (3708 [0.08%]).

### Extent of Coprescription

#### Unadjusted Rates

Among patients using long-term opioids, approximately one-quarter of person-months involved overlapping opioid and benzodiazepine prescriptions (mean [SE], commercial group, 23.0% [0.18%]; MA group, 25.7% [0.18%]). Opioid and benzodiazepine coprescription was less common during short-term opioid use episodes compared with long-term opioid episodes (Table 1).

#### Adjusted Rates

**Figure 1.**
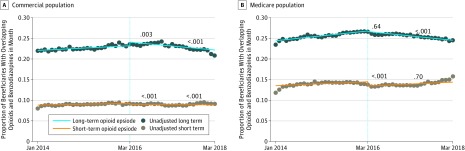
Extent of Coprescription of Opioids and Benzodiazepines Before and After the Centers for Disease Control and Prevention Guideline Release Adjusted proportion of person-months with overlapping opioid and benzodiazepine prescriptions in the population of commercially insured individuals (A) and Medicare Advantage beneficiaries (B). Statistical significance was set at *P* = .05. Statistical significance for change in level is indicated next to dashed line, which denotes release of opioid guidelines. Statistical significance for change in trend (line slope) is indicated further to the right of the statistical significance of the change in level. Point estimates and 95% confidence intervals are shown in eTable 6 in the Supplement.

Before the CDC guidelines were released, the extent of coprescription was increasing in both the commercial and MA long-term opioid use populations (preguideline slope for commercial long-term use population, 0.52 percentage point per year [95% CI, 0.02-1.03 percentage points per year]; preguideline slope for MA long-term use population, 1.09 percentage points per year [95% CI, 0.56-1.62 percentage points per year]) (Figure 1 and Table 2). The release of the CDC guidelines was associated with a decrease in trend for both of these populations, resulting in a negative slope after release of approximately 1 percentage point per year (−0.95 percentage point per year [95% CI, −1.44 to −0.46 percentage points per year] for the commercial group and −1.06 percentage points per year [95% CI, −1.49 to −0.63 percentage points per year] for the MA group; the postguideline trend minus preguideline trend was −0.12% per month for the commercial population and −0.18% per month for the MA population). These changes were statistically significant after controlling for a familywise error rate of 0.05 across all comparisons in extent and intensity of coprescription. The guidelines were associated with a 0.87–percentage point increase in the level of coprescribing in the commercial population, resulting in similar coprescribing rates at the beginning and end of the study period. There was no statistically significant change in level for the MA population using opioids long term. Complete regression results are available in eTables 2, 3, 4, and 5 in the Supplement; adjusted rates with confidence intervals are available in eTable 6 in the Supplement.

**Table 2.** Changes in Extent and Intensity of Opioid and Benzodiazepine Coprescribing Associated With Centers for Disease Control and Prevention Guideline Release

Population or Opioid Episode Type	Commercial Insurance				Medicare Advantage			
	Long Term		Short Term		Long Term		Short Term	
	Value (95% CI)	*P* Value^a^	Value (95% CI)	*P* Value^a^	Value (95% CI)	*P* Value^a^	Value (95% CI)	*P* Value^a^
Extent of coprescription: adjusted rates of overlapping opioids and benzodiazepines, mo^b^								
Change in level just before to just after guideline release, %	0.87 (0.29 to 1.45)	.003^c^	−0.25 (−0.35 to −0.15)	<.001^c^	−0.14 (−0.72 to 0.45)	.64	−1.02 (−1.21 to −0.83)	<.001^c^
Preguideline release slope, %/y	0.52 (0.02 to 1.03)		0.19 (0.13 to 0.24)		1.09 (0.56 to 1.62)		0.44 (0.32 to 0.56)	
Postguideline release slope, %/y	−0.95 (−1.44 to −0.46)	<.001^c^	−0.05 (−0.12 to 0.02)	<.001^c^	−1.06 (−1.49 to −0.63)	<.001^c^	0.47 (0.35 to 0.59)	.70
Intensity of coprescription: adjusted percentage of opioid prescription days with concurrent benzodiazepines among those with any overlap in the month^b^								
Change in level just before to just after guideline release, %	−0.08 (−0.74 to 0.57)	.80	−0.31 (−0.68 to 0.07)	.11	−0.32 (−0.87 to 0.22)	.25	−0.17 (−0.56 to 0.23)	.41
Preguideline release slope, %/y	0.57 (0.04 to 1.11)		0.19 (−0.02 to 0.39)		0.56 (0.09 to 1.02)		0.28 (0.03 to 0.52)	
Postguideline release slope, %/y	0.27 (−0.25 to 0.79)	.45	0.30 (0.03 to 0.56)	.53	0.84 (0.48 to 1.20)	.37	0.34 (0.11 to 0.58)	.70

^a^ *P* value for change in level tests whether change in level equals 0; *P* value for postguideline release slope tests whether postguideline release slope equals preguideline release slope.

^b^ Adjusted rates of overlapping opioid and benzodiazepine prescriptions filled represent predictive margins from a logit model that included the covariates patient age, sex, race/ethnicity, state of residence, and Elixhauser comorbidity flags calculated on a rolling 6-month basis. Adjusted rates of opioid prescription days with benzodiazepines represent predictive margins from negative binomial models specified with the same covariates as specified in the previous sentence, using the number of opioid prescription days in the month as an exposure variable (ie, the natural log of opioid prescription days was included in the model with the coefficient constrained to be 1). Separate models were specified for each patient population and opioid episode type (ie, commercial long term, commercial short term, Medicare Advantage long term, and Medicare Advantage short term). The SEs in the models were adjusted for clustering on individual patient.

^c^ Statistically significant after controlling for familywise error rate of 0.05 within table.

The comparison group of short-term opioid use episodes in the MA and commercial populations showed smaller changes or no change. In the MA short-term use population, the extent of coprescribing was increasing at approximately the same rate before and after the guideline release (slope, 0.44 percentage point [95% CI, 0.32-0.56 percentage point] before and 0.47 percentage point [95% CI, 0.35-0.59 percentage point] after; *P* = .70); a statistically significant decrease of −1.02 percentage points (95% CI, −1.21 to −0.83; *P* < .001 after controlling for familywise error rate of 0.05) attenuated the overall trend toward increasing coprescribing in this population. In the commercially insured population with short-term opioid use, the trend before guideline release was a slight increase (slope, 0.19 percentage point per year [95% CI, 0.13-0.24 percentage point per year]). After guideline release, the slope was no longer increasing (not statistically different from 0); this change was statistically significant (postguideline slope, −0.05 percentage point per year [95% CI, −0.12 to 0.02 percentage point per year]; *P* < .001 after controlling for familywise error rate of 0.05). There was a small, statistically significant change of −0.25 percentage point (95% CI, −0.35 to −0.15; *P* < .001 after controlling for familywise error rate of 0.05) associated with guideline release in the commercial short-term use group.

### Intensity of Coprescription

#### Unadjusted Rates

Intensity of coprescription was measured for participants with long-term opioid use episodes who had at least 1 day of overlapping benzodiazepine and opioid prescriptions in the months evaluated. Coprescription intensity was 79.3% (95% CI, 78.9%-79.6%) in the commercial group and 83.9% (95% CI, 83.7%-84.2%) in the MA population (Table 1).

#### Adjusted Rates

**Figure 2.**
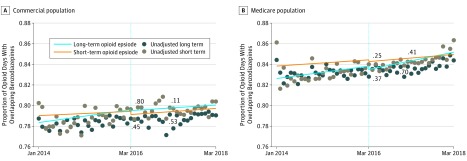
Intensity of Coprescription of Opioids and Benzodiazepines Before and After the Centers for Disease Control and Prevention Guideline Release Adjusted proportion of opioid prescription days with overlapping benzodiazepine prescriptions in the population of commercially insured individuals (A) and Medicare Advantage beneficiaries (B). Statistical significance was set at *P* = .05. Statistical significance for change in level is indicated next to the dashed line, which denotes the release of the opioid guidelines. Statistical significance for change in trend (line slope) is indicated further to the right of the statistical significance of the change in level. Point estimates and 95% confidence intervals are shown in eTable 6 in the Supplement.

There were no statistically significant changes in either level or trend of intensity of coprescription of opioids and benzodiazepines in any beneficiary or opioid use group (Figure 2 and Table 2). Slopes were positive for all beneficiary and opioid use groups and periods except for long-term opioid use episodes in the commercial group in the postguideline period, where the slope was not statistically significantly different from 0. However, these slopes were exceptionally small; for example, among commercial insurance beneficiaries with long-term opioid use episodes, the intensity of coprescription was increasing by 0.57% per year (95% CI, 0.04%-1.11% per year) before the guidelines were released, on a baseline intensity rate of 78.36% (95% CI, 77.53%-79.185%), for a relative change of 0.73% per year. Complete regression results are available in eTables 7, 8, 9, and 10 in the Supplement.

### Same Physician Prescribing

#### Unadjusted Proportions

Overlapping prescriptions for opioids and benzodiazepines were frequently written by the same physician. For commercial participants with long-term use episodes, a mean (SE) of 59.1% (0.12%) of overlapping prescriptions were from the same physician, and for MA beneficiaries, a mean (SE) of 60.3% (0.10%) of overlapping prescriptions were from the same physician (Table 1).

#### Adjusted Proportions

**Figure 3.**
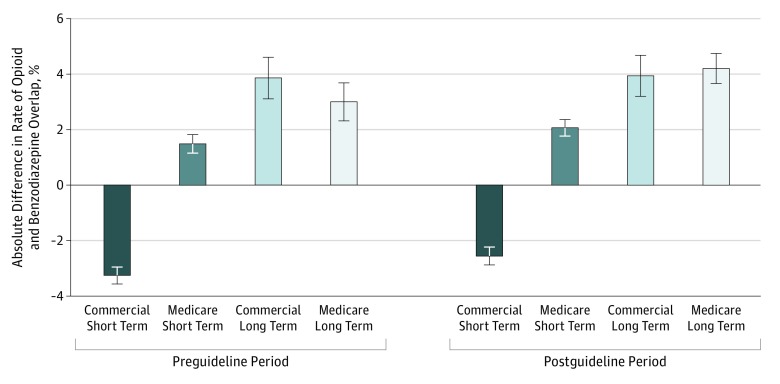
Difference in Intensity With Same vs Different Physicians Prescribing Opioids and Benzodiazepines Error bars denote 95% confidence intervals, and column height indicates the percentage point increase in the rate of coprescribing (overlap days in month per opioid prescription days in month). For example, in the commercial population using long-term opioids before the guideline release, the regression-adjusted proportion of opioid prescription days with overlapped benzodiazepines was 80.5% with the same physician prescribing and 76.6% with different physicians prescribing. The absolute difference is 80.5% − 76.6% = 3.9%. All differences were statistically different from 0 when adjusted for a familywise error rate of 0.05. Point estimates and differences with 95% confidence intervals are provided in eTable 15 in the Supplement.

Figure 3 shows the difference in rates of overlap for patients with the same prescribing physician compared with those with different physicians prescribing opioids and benzodiazepines. In months with any coprescription during long-term opioid use episodes, having the same physician prescribe both opioids and benzodiazepines was associated with a small but consistent increase in the proportion of overlap days in both patient populations. This association was present both before and after the release of the CDC guidelines.

For beneficiaries with short-term opioid use episodes, conditional on having any overlap in the month, MA beneficiaries experienced greater coprescription intensity when the same physician prescribed both drugs, compared with having different prescribers for the 2 drugs, whereas commercial insurance beneficiaries actually had reduced coprescription intensity when the same physician prescribed both drugs. Complete regression results are available in eTables 11, 12, 13, and 14 in the Supplement; adjusted rates with confidence intervals are available in eTable 15 in the Supplement.

### Secondary Analyses

#### Race/Ethnicity-Stratified Analysis

White and Hispanic beneficiaries had higher rates of coprescription than did black beneficiaries over the entire study period (eFigure 2 and eTables 16, 17, 18, and 19 in the Supplement). There were no statistically significant changes in trend or level for black and Hispanic beneficiaries with long-term opioid use episodes (mean [SD] monthly sample size, 4522 [1684] for black and 2565 [839] for Hispanic beneficiaries).

#### Opioid Dose–Stratified Analysis

The risk of overdose in people taking both opioids and benzodiazepines increases with opioid dose.^3^ We compared coprescribing extent by opioid dose: less than 20 MMEs, 20 to less than 50 MMEs, 50 to less than 90 MMEs, 90 to less than 120 MMEs, and 120 or more MMEs of opioids per opioid use day. We found that the extent of coprescribing was highest in the 120 or more MME group, with adjusted proportions at the end of the study of 35.9% (95% CI, 33.8%-38.0%) of person-months in the long-term opioid use population and 19.6% (95% CI, 18.7%-20.4%) of person-months in the short-term opioid use population (eFigure 3, eTable 20, and eTable 21 in the Supplement). Statistically significant decreases in slope were seen in the 20 to less than 50 MME and 50 to less than 90 MME long-term opioid use dose groups. Statistically significant increases in slope were found in the short-term opioid use group at those same dose levels (20 to < 50 MME and 50 to < 90 MME).

#### Coprescribing Extent in Episodes Starting Before vs After Guideline Release

A sensitivity analysis was specified post hoc to look only at long-term episodes starting before vs after the guideline release. This analysis found no change in the extent of coprescribing level or trend in the first 90 days of the episode for commercial beneficiaries and a statistically significant decrease in trend for MA beneficiaries (MA preguideline slope, 0.22 percentage point per year [95% CI, −0.54 to 0.99 percentage point per year]; MA postguideline slope, −1.23 percentage points per year [95% CI, −2.26 to −0.32 percentage points per year]) (eTable 22 and eTable 23 in the Supplement).

## Discussion

After the release of the CDC guidelines addressing long-term opioid prescribing, previously increasing trends in the extent of coprescribing of opioids and benzodiazepines during long-term opioid use episodes were observed to reverse, with decreasing trends over time seen after the CDC guideline release. The coprescribing extent in short-term opioid use episodes saw smaller decreases or no change. There were no differences in the intensity of coprescribing after the release of the CDC guidelines.

Bohnert et al^24^ reported that, according to IQVIA retail pharmacy data for the years 2015 to 2017, coprescribing across all patient populations was declining both before and after the CDC guidelines. We did not find this in our observed populations, who demonstrated increasing or stable rates of coprescribing before the CDC guidelines. This difference could be due to a difference in the populations studied, because the article by Bohnert et al^24^ included people who paid in cash or used Medicaid, who are not present in our analysis. This difference may also reflect differences in study designs. Unlike the study by Bohnert et al,^24^ we separately report trends in long-term vs short-term opioid use episodes and for MA vs commercial insurance beneficiaries. The differences between the 2 studies could be an example of the Simpson paradox,^25^ where trends in stratified analyses are reversed in the unstratified analysis. As a concrete example, given the large difference in overall coprescription rates between long-term and short-term opioid episodes, if the relative proportion of short-term episodes increased (or if the length of long-term episodes increased), the overall rate of coprescription would decrease, even while the coprescription rate in short-term episodes increased.

Our study adds to the marketwide findings by disaggregating the population into people using opioids long term, who are the target of the guidelines and at greatest risk for adverse events, vs people using opioids short-term. We also considered both how common coprescription is (ie, its extent) as well as how much overlap patients experience when they have coprescribed drugs (ie, the intensity of coprescription). We found that the release of the guidelines was associated with a reduction in the extent of coprescribing in long-term opioid use episodes, but not with a change in intensity of coprescribing.

We also characterized coprescribing by the same physician vs the case when different physicians prescribe the opioids and benzodiazepines. We found that most of the time, the same physician was prescribing both opioids and benzodiazepines and that when the same physician prescribed both opioids and benzodiazepines, the intensity of coprescription increased. In other words, coprescription seemed not to be driven by lack of information or coordination among treating physicians.

One explanation for this finding could be that physicians coprescribe opioids and benzodiazepines to treat co-occurring pain and mental health conditions. Chronic pain and mental health disorders are common in the general population, and high rates of co-occurrence have been reported.^26^ The estimated current or 12-month frequency of depressive symptoms exceeds 50% among adults with fibromyalgia,^27,28,29^ temporomandibular joint disorder,^30,31^ chronic spinal pain,^32,33,34^ and chronic abdominal pain.^35,36^ Similarly, the 12-month frequency of anxiety exceeds 50% in individuals with temporomandibular joint disorder,^37,38^ fibromyalgia,^29,39^ and chronic abdominal pain.^35,36^ Although benzodiazepines are generally indicated for the treatment of anxiety disorders, their use in adults with chronic pain has been associated with greater levels of depression, greater pain scores, and use of other psychotropic medications.^40,41^

Physicians treating people with co-occurring pain and anxiety may be choosing to treat with opioids and benzodiazepines, perhaps after other treatments have failed. They may also have patients with an established regimen of either opioids or benzodiazepines who later develop anxiety (in the case of opioids) or pain (in the case of benzodiazepines). Coprescription may result because the patient does not want to or is unable to discontinue one of the drugs.

Having seen relatively minor changes in coprescribing, we specified a post hoc analysis to explore whether physicians were less likely to initiate coprescription after the CDC guidelines were released. We reasoned that for beneficiaries who did not have an established history of using 1 or both drugs, physicians may have faced fewer barriers to avoiding this practice altogether. This assumption was tested with a comparison of rates of coprescription in the first 90 days of new long-term episodes of opioid use that started before the guidelines were released vs those that started after the guidelines were released. In the MA population, we observed a decrease in trend for new coprescribing after the guidelines, although no change was seen in the commercial population. The contradictory findings may suggest that physicians consider MA beneficiaries to face higher risks from coprescription, but our study was not designed to test this hypothesis.

Guidelines are a passive intervention to change physician behavior and might not be expected to yield substantial impact. We can compare our results with the 2013 implementation of the Opioid Safety Initiative, a Veterans Health Administration initiative to reduce coprescription of benzodiazepines and high-dose opioids.^42^ At the beginning of the study period, rates of coprescribing were already decreasing. Segmented regression found the Opioid Safety Initiative was associated with a change in trend of −0.11% per month. This is similar to the association seen in long-term opioid use episodes in our study (postguideline trend minus preguideline trend was −0.12% per month for the commercial population and −0.18% per month for the MA population).

Studies of the uptake of clinical guidelines have explored the reasons physicians do not adhere to guideline recommendations.^43^ The framework by Pathman et al^44^ suggests that, before adhering to a guideline, physicians must become aware of the guideline, agree with the content, and adopt the recommendations. The increase in overlap intensity when the same physician prescribes both drugs may suggest that some physicians do not agree with the CDC’s recommendation to avoid coprescribing opioids and benzodiazepines. Alternatively, some physicians may have adopted the recommendation but made the clinical decision not to adhere to the recommendation for select patients either because some patients were resistant to making the recommended pharmacological changes or the physician believed the patient was benefiting from the coprescription of both drugs. Observing little change in coprescription intensity could suggest the guidelines had little impact or, alternatively, that physicians who continued to coprescribe were coprescribing for patients for whom the balance of risks and benefits was favorable. However, the high intensity of coprescribing, particularly among those with the highest opioid doses, suggests that some patients remain at risk because of inappropriate prescribing. Future studies should explore changes in coprescribing at the physician and patient levels to further understand changes in physician behavior and its impact on patients.

### Limitations

As a retrospective study using administrative data, this study is subject to limitations. The study results represent an association rather than a causal effect of the guideline release on coprescription of opioids and benzodiazepines. The data also may be missing prescriptions filled by beneficiaries who paid cash at the pharmacy, which could affect the ascertainment of the outcome measures. Our data provide trends for 2.25 years before and 2 years after the guideline release, and trends may change if they are observed for shorter or longer periods. Also, this administrative data set does not include details necessary to determine the appropriateness of coprescribing.

## Conclusions

Coprescription of opioids and benzodiazepines was common among this sample of commercially insured and MA beneficiaries, both before and after the release of the CDC guidelines. After guideline release, the extent of coprescribing decreased by a modest amount in people using opioids long term, but not those using opioids short term. There was no change associated with the guidelines in the intensity of coprescribing for any population. Future studies focused on identifying patterns of physician response to these guidelines may provide insight into who was affected by the guideline release on coprescription of opioids and benzodiazepines.

## Notes

Supplement. **eAppendix 1.** Identification of Opioids and Conversion to Morphine Milligram Equivalents **eAppendix 2.** Details of the Statistical Analysis Methodology **eAppendix 3.** Elixhauser Comorbidity Calculations **eAppendix 4.** Cancer **eAppendix 5.** Sickle Cell Disease **eAppendix 6.** Hospice **eTable 1.** Opioid Drugs and Combinations Included **eTable 2.** Regression Results: Extent of Coprescribing for the Commercially Insured—Short-term Opioid Use Episodes **eTable 3.** Regression Results: Extent of Coprescribing for the Commercially Insured—Long-term Opioid Use Episodes **eTable 4.** Regression Results: Extent of Coprescribing for Medicare Advantage—Short-term Episodes **eTable 5.** Regression Results: Extent of Coprescribing for Medicare Advantage—Long-term Episodes **eTable 6.** Adjusted Estimates: Extent and Intensity of Coprescription **eTable 7.** Regression Results: Intensity of Coprescribing for the Commercially Insured—Short-term Episodes **eTable 8.** Regression Results: Intensity of Coprescribing for the Commercially Insured—Long-term Episodes **eTable 9.** Regression Results: Intensity of Coprescribing for Medicare Advantage—Short-term Episodes **eTable 10.** Regression Results: Intensity of Coprescribing for Medicare Advantage—Long-term Episodes **eTable 11.** Regression Results: Intensity of Coprescription Among Commercial Beneficiaries With and Without Same Physician Prescribing Opioids and Benzodiazepines—Short-term Opioid Use Episodes **eTable 12.** Regression Results: Intensity of Coprescription Among Commercial Beneficiaries With and Without Same Physician Prescribing Opioids and Benzodiazepines: Long-term Opioid Use Episodes **eTable 13.** Regression Results: Intensity of Coprescription Among Medicare Advantage Beneficiaries With and Without Same Physician Prescribing Opioids and Benzodiazepines—Short-term Episodes **eTable 14.** Regression Results: Intensity of Coprescription Among Medicare Advantage Beneficiaries With and Without Same Physician Prescribing Opioids and Benzodiazepines—Long-term Episodes **eTable 15.** Intensity of Coprescribing Before and After the Guideline Release: Comparing Same Physician Prescribing to Different Physicians Prescribing Opioids and Benzodiazepines **eTable 16.** Extent of Coprescription by Race/Ethnicity: Adjusted Percent of Overlapping Opioids and BZDs in Month **eTable 17.** Changes in Extent of Opioid and Benzodiazepine Coprescribing Associated With CDC Guideline Release, by Race/Ethnicity **eTable 18.** Regression Results Secondary Analysis: Race/Ethnicity Short-term Episodes **eTable 19.** Regression Results Secondary Analysis: Race/Ethnicity Long-term Episodes **eTable 20.** Extent of Coprescription by Opioid Dose: Adjusted Percent With Overlapping Opioids and Benzodiazepines in Month **eTable 21.** Changes in Extent of Opioid and Benzodiazepine Coprescribing Associated With CDC Guideline Release, by Opioid Dose **eTable 22.** Regression Results Sensitivity Analysis: Episodes Starting Before vs After Guideline Release **eTable 23.** Adjusted Coprescribing Extent Estimates and Trends in Long-term Opioid Use Episodes Starting Before vs After the Guidelines Were Released **eFigure 1.** Cohort Flow Chart Describing Construction of the Study Sample **eFigure 2.** Race/Ethnicity: Coprescribing Extent **eFigure 3.** Coprescribing Extent by Opioid Dose **eReferences**
