# Supplementary material for: Rates of Physician Coprescribing of Opioids and Benzodiazepines After the Release of the Centers for Disease Control and Prevention Guidelines in 2016
Source: JAMA Netw Open. 2019 Aug 2;2(8):e198325. doi: 10.1001/jamanetworkopen.2019.8325 (PMC6681551; doi:10.1001/jamanetworkopen.2019.8325)
Supplement: Supplement. — eAppendix 1. Identification of Opioids and Conversion to Morphine Milligram Equivalents eAppendix 2. Details of the Statistical Analysis Methodology eAppendix 3. Elixhauser Comorbidity Calculations eAppendix 4. Cancer eAppendix 5. Sickle Cell Disease eAppendix 6. Hospice eTable 1. Opioid Drugs and Combinations Included eTable 2. Regression Results: Extent of Coprescribing for the Commercially Insured—Short-term Opioid Use Episodes eTable 3. Regression Results: Extent of Coprescribing for the Commercially Insured—Long-term Opioid Use Episodes eTable 4. Regression Results: Extent of Coprescribing for Medicare Advantage—Short-term Episodes eTable 5. Regression Results: Extent of Coprescribing for Medicare Advantage—Long-term Episodes eTable 6. Adjusted Estimates: Extent and Intensity of Coprescription eTable 7. Regression Results: Intensity of Coprescribing for the Commercially Insured—Short-term Episodes eTable 8. Regression Results: Intensity of Coprescribing for the Commercially Insured—Long-term Episodes eTable 9. Regression Results: Intensity of Coprescribing for Medicare Advantage—Short-term Episodes eTable 10. Regression Results: Intensity of Coprescribing for Medicare Advantage—Long-term Episodes eTable 11. Regression Results: Intensity of Coprescription Among Commercial Beneficiaries With and Without Same Physician Prescribing Opioids and Benzodiazepines—Short-term Opioid Use Episodes eTable 12. Regression Results: Intensity of Coprescription Among Commercial Beneficiaries With and Without Same Physician Prescribing Opioids and Benzodiazepines: Long-term Opioid Use Episodes eTable 13. Regression Results: Intensity of Coprescription Among Medicare Advantage Beneficiaries With and Without Same Physician Prescribing Opioids and Benzodiazepines—Short-term Episodes eTable 14. Regression Results: Intensity of Coprescription Among Medicare Advantage Beneficiaries With and Without Same Physician Prescribing Opioids and Benzodiazepines—Long-term Episodes eTable 15. Int [file jamanetwopen-2-e198325-s001.pdf]

Supplementary Online Content

Jeffery MM, Hooten WM, Jena AB, Ross JS, Shah ND, Karaca-Mandic P. Rates of physician coprescribing of opioids and benzodiazepines after the release of the Centers for Disease Control and Prevention guidelines in 2016. *JAMA Netw Open*. 2019;2(8):e198325. doi:10.1001/jamanetworkopen.2019.8325

eAppendix 1. Identification of Opioids and Conversion to Morphine Milligram Equivalents
eAppendix 2. Details of the Statistical Analysis Methodology
eAppendix 3. Elixhauser Comorbidity Calculations
eAppendix 4. Cancer
eAppendix 5. Sickle Cell Disease
eAppendix 6. Hospice
eTable 1. Opioid Drugs and Combinations Included
eTable 2. Regression Results: Extent of Coprescribing for the Commercially Insured—Short-term Opioid Use Episodes
eTable 3. Regression Results: Extent of Coprescribing for the Commercially Insured—Long-term Opioid Use Episodes
eTable 4. Regression Results: Extent of Coprescribing for Medicare Advantage—Short-term Episodes
eTable 5. Regression Results: Extent of Coprescribing for Medicare Advantage—Long-term Episodes
eTable 6. Adjusted Estimates: Extent and Intensity of Coprescription
eTable 7. Regression results: Intensity of Coprescribing for the Commercially Insured—Short-term Episodes
eTable 8. Regression Results: Intensity of Coprescribing for the Commercially Insured—Long-term Episodes
eTable 9. Regression Results: Intensity of Coprescribing for Medicare Advantage—Short-term Episodes
eTable 10. Regression Results: Intensity of Coprescribing for Medicare Advantage— Long-term Episodes
eTable 11. Regression Results: Intensity of Coprescription Among Commercial Beneficiaries With and Without Same Physician Prescribing Opioids and Benzodiazepines—Short-term Opioid Use Episodes
eTable 12. Regression Results: Intensity of Coprescription Among Commercial Beneficiaries With and Without Same Physician Prescribing Opioids and Benzodiazepines: Long-term Opioid Use Episodes
eTable 13. Regression Results: Intensity of Coprescription Among Medicare Advantage Beneficiaries With and Without Same Physician Prescribing Opioids and Benzodiazepines—Short-term Episodes
eTable 14. Regression Results: Intensity of Coprescription Among Medicare Advantage Beneficiaries With and Without Same Physician Prescribing Opioids and Benzodiazepines—Long-term Episodes

© 2019 Jeffery MM et al. *JAMA Network Open.*

eTable 15. Intensity of Coprescribing Before and After the Guideline Release: Comparing Same Physician Prescribing to Different Physicians Prescribing Opioids and Benzodiazepines
eTable 16. Extent of Coprescription by Race/Ethnicity: Adjusted Percent of Overlapping Opioids and BZDs in Month
eTable 17. Changes in Extent of Opioid and Benzodiazepine Coprescribing Associated With CDC Guideline Release, by Race/Ethnicity
eTable 18. Regression Results Secondary Analysis: Race/Ethnicity Short-term Episodes
eTable 19. Regression Results Secondary Analysis: Race/Ethnicity Long-term Episodes
eTable 20. Extent of Coprescription by Opioid Dose: Adjusted Percent With Overlapping Opioids and Benzodiazepines in Month
eTable 21. Changes in Extent of Opioid and Benzodiazepine Coprescribing Associated With CDC Guideline Release, by Opioid Dose
eTable 22. Regression Results Sensitivity Analysis: Episodes Starting Before vs After Guideline Release
eTable 23. Adjusted Coprescribing Extent Estimates and Trends in Long-term Opioid Use Episodes Starting Before vs After the Guidelines Were Released
eFigure 1. Cohort Flow Chart Describing Construction of the Study Sample
eFigure 2. Race/Ethnicity: Coprescribing Extent
eFigure 3. Coprescribing Extent by Opioid Dose
eReferences

This supplementary material has been provided by the authors to give readers additional information about their work.

© 2019 Jeffery MM et al. *JAMA Network Open.*

eAppendix 1. Identification of Opioids and Conversion to Morphine Milligram Equivalents

We identified all opioid drugs present in the table of NDC codes in OptumLabs Data Warehouse. For the purposes of this analysis, we classified tramadol as an opioid. To limit the sample to drugs intended for home use, we excluded any injected or infused drug—those for which the dosage form was vial, syringe, ampule, cartridge, IV solution, etc. We included only drugs which had a defined dose unit like a tablet, pill, mg/mL, etc. This excludes drugs in powder or bulk form. We included both single drug formulations and combinations of drugs. eTable 1 includes all opioid drug combinations found in the table of NDC codes. Both long-acting and short-acting formulations were included. Conversion factors were taken from the CDC compilation.[1]

eAppendix 2. Details of the Statistical Analysis Methodology

Changes in coprescribing were analyzed both as a one-time change in level at the time of CDC guideline release and as a change in trend after the guideline release. To implement this approach, piecewise regression with a single knot spline was used and three main exposure variables were created. The variables *PreRelease* and *Release* were components of a spline with a knot at the release of the guidelines (March 2016). *PreRelease* measured the trend in the outcome variable before the intervention, while *Release* captured the change in trend after the intervention. *AfterRelease* was an indicator for time after March 2016. This indicator was used to measure the change in level of the outcome variable from just before to just after the guidelines release.

The unit of observation for the analysis of the extent of coprescription was a person-month during which the beneficiary had opioids available for at least one day; the unit of observation for the analysis of the intensity of coprescription was a person-month during which

© 2019 Jeffery MM et al. *JAMA Network Open.*

the beneficiary had at least one day of overlapping opioids and benzodiazepines.. A generalized linear model was specified with clustered standard errors to account for repeated observations on the same patients. The model was adjusted for beneficiary covariates age, sex, race/ethnicity, state of residence, and a series of indicator variables for each of the Elixhauser comorbidities[2] measured on a rolling 6 month basis. A logit specification was used to assess the extent of coprescription, while a negative binomial specification was used to assess intensity of coprescription. In the latter model, the natural log of the number of opioid days was included with the coefficient constrained to 1 to allow interpretation of the incidence rates as the proportion of overlapping days of opioids and benzodiazepines. In the main analyses of intensity and extent of coprescribing, separate models were estimated for the two beneficiary insurance populations (commercial and MA) and two types of opioid use episodes (long-term and short-term). For the subanalysis of coprescribing extent by race and ethnicity, commercial and MA populations were modeled together, with a binary variable indicating population added to the model.

In a sensitivity analysis, coprescribing by the same physician was further explored in comparison to one physician prescribing the benzodiazepine and another prescribing the opioid. This specification included interactions of the three main exposure variables with a categorical variable measuring whether the same physician prescribed the opioids and benzodiazepines; this model was limited to person-months with any overlap days. The categorical variable could take the following values; (1) at least one day of overlap with prescriptions written by different physicians; (2) at least one day of overlap with prescriptions written by the same physician; (3) at least one day of overlap, but it was not possible to assess whether the same physician prescribed the drugs because of unknown prescriber identification numbers on one or both of the

© 2019 Jeffery MM et al. *JAMA Network Open.*

prescriptions. Some beneficiaries had multiple opioid and multiple benzodiazepine prescriptions available in the same month. In those cases, if there was at least one pair of overlapping opioid and benzodiazepine prescriptions from the same physician, the categorical variable was set to "same physician." A truncated negative binomial model was used because the outcome distribution could not take the value 0.

A post-hoc sensitivity analysis was specified to assess initiation of benzodiazepines in the first 90 days of new long-term opioid use episodes that began before the guideline release versus those that began after the guideline release. Separate models were specified for commercial and Medicare Advantage populations.

The Holm sequentially rejective multiple test procedure was used to control the family-wise error rate at 0.05.[3] Tables, text, and figures denote with asterisks whether comparisons are statistically significant after this adjustment. For the purpose of controlling the error rates, the analyses of extent and intensity of coprescribing were treated as a single family of analyses, as were the analyses of same physician prescribing, the analyses by race and ethnicity, and the analyses of long-term episodes beginning before and after the guideline release.

Summary statistics are presented as percent and standard error (SE) for categorical variables, and as median and interquartile range (IQR, 25th percentile, 75th percentile) for continuous variables.

Complete regression results are available in the Supplementary Appendix Tables S3-S7 and S14 along with further detail on model specifications. Figures present adjusted rates of overlapping opioid and benzodiazepine fills, and adjusted proportion of opioid days where benzodiazepines were also available (overlap days), with 95 confidence intervals in tables;

© 2019 Jeffery MM et al. *JAMA Network Open.*

unadjusted values are plotted along with lines representing the adjusted rates. All analyses were performed with Stata/MP version 15.0.[4]

eAppendix 3. Elixhauser Comorbidity Calculations

ICD 9 and 10 codes from Quan et al.[2] were used to define the Elixhauser comorbidities. We created flags for the presence of at least one inpatient or two outpatient dates of service with a diagnosis from the Elixhauser definitions. Flags were created for each month, looking back at the prior 6 months of health care service use. The flags were set to 1 if the definition was met, or 0 if it was not. A separate flag was created to indicate whether the beneficiary had not had 6 prior months of coverage and was included separately in the models.

eAppendix 4. Cancer

To exclude beneficiaries with cancer, we looked for at least 2 evaluation and management procedure codes on different dates of service where one of the first 4 diagnoses on the claim was a cancer diagnosis. An opioid episode was excluded if the beneficiary had at least 2 cancer evaluation and management claims during the period from 3 months before the start of the episode through the end of the episode. The codes used to define cancer for the purpose of excluding opioid use episodes were ICD9 codes 140 through 209, ICD9 codes 230 through 239, ICD10 codes C00 through C97, ICD10 codes D00 through D09, and ICD10 codes D37 through D47.

eAppendix 5. Sickle Cell Disease

To exclude beneficiaries with sickle cell disease, we used the same procedure as used to exclude episodes for cancer. The codes used to define sickle cell disease were ICD9 codes 282.41, 282.42, 282.6x;[5] and ICD10 codes D570.x, D571.x, D574.x, and D578.x.

© 2019 Jeffery MM et al. *JAMA Network Open.*

eAppendix 6. Hospice

We excluded any opioid use episode with one or more hospice procedure or revenue codes during the period from 3 months before the start of the episode through the end of the episode. Hospice procedure codes were 99377, 99378, G0182, G0337, G9474-G9479, G9524, Q5001-Q5010, S0255, S9126, and T2042-T2046. Hospice revenue codes were 0115, 0125, 0135, 0145, 0155, 0235, and 065x.

eTable 1. Opioid Drugs and Combinations Included

Opioid	Drug combinations included	Long acting	Short acting	Conversion factor*
**Buprenorphine**	Buprenorphine	X		10<br>Patch version: 12.6
	Buprenorphine/Naloxone	X		10
**Butorphanol**	Butorphanol		X	7
**Codeine**	Codeine		X	0.15
	Codeine/Acetaminophen		X	0.15
	Codeine/Acetaminophen/ Butabarbital		X	0.15
	Codeine/Acetaminophen/ Butalbital		X	0.15
	Codeine/Aspirin		X	0.15
	Codeine/Aspirin/Butalbital/ Caffeine		X	0.15
	Codeine/Aspirin/Carisoprodol		X	0.15
	Codeine/Aspirin/Phenacetin/ Caffeine		X	0.15
**Dihydrocodeine**	Dihydrocodeine/Acetaminophen/ Caffeine		X	0.25
	Dihydrocodeine/Aspirin/Caffeine		X	0.25
**Fentanyl**	Fentanyl	X	X	LA: 0.72<br>SA: 0.13 - 0.18
**Hydrocodone**	Hydrocodone	X	X	1
	Hydrocodone/Acetaminophen		X	1
	Hydrocodone/Acetaminophen/Diet.Sup.11		X	1
	Hydrocodone/Aspirin		X	1
	Hydrocodone/Ibuprofen		X	1
**Hydromorphone**	Hydromorphone	X	X	4
**Levomethadyl**	Levomethadyl	X		8
**Levorphanol**	Levorphanol	X		11
**Meperidine**	Meperidine/Acetaminophen		X	0.1
	Meperidine/Promethazine		X	0.1
**Methadone**	Methadone	X		3

© 2019 Jeffery MM et al. *JAMA Network Open.*

Opioid	Drug combinations included	Long acting	Short acting	Conversion factor*
**Morphine**	Morphine Sulfate	X	X	1
	Morphine Sulfate/Naltrexone	X		1
**Opium**	Opium		X	1
	Opium/Belladonna		X	1
**Oxycodone**	Oxycodone	X	X	1.5
	Oxycodone/Acetaminophen		X	1.5
	Oxycodone/Aspirin		X	1.5
	Oxycodone/Ibuprofen		X	1.5
**Oxymorphone**	Oxymorphone	X	X	3
**Pentazocine**	Pentazocine/Acetaminophen		X	0.37
	Pentazocine/Aspirin		X	0.37
	Pentazocine/Naloxone		X	0.37
**Tapentadol**	Tapentadol	X	X	0.4
**Tramadol**	Tramadol	X	X	0.1
	Tramadol/Acetaminophen		X	0.1
	Tramadol/Dietary Supplement No. 11		X	0.1
* Factor used to convert mg of drug to mg of morphine equivalents[1]				

© 2019 Jeffery MM et al. *JAMA Network Open.*

eTable 2. Regression Results: Extent of Coprescribing for the Commercially Insured—Short-term Opioid Use Episodes

Outcome: any overlap days<br>Population: commercial, short-term episodes	Coef.	Robust Std. Err.	z	P>z	[95 Conf.	Interval]
Spline components						
months before release	0.002	0.000	6.540	0.000	0.001	0.003
months after release	-0.003	0.001	-4.980	0.000	-0.004	-0.002
binary post-release	-0.032	0.007	-4.830	0.000	-0.044	-0.019
state						
AL	-0.003	0.099	-0.030	0.974	-0.197	0.190
AR	0.098	0.098	1.010	0.315	-0.094	0.291
AZ	-0.081	0.097	-0.840	0.402	-0.270	0.108
CA	-0.032	0.096	-0.330	0.741	-0.221	0.157
CO	-0.029	0.096	-0.300	0.763	-0.218	0.160
CT	0.042	0.100	0.420	0.671	-0.153	0.237
DC	-0.219	0.107	-2.050	0.040	-0.428	-0.010
DE	-0.163	0.122	-1.340	0.181	-0.402	0.076
FL	0.156	0.096	1.630	0.104	-0.032	0.345
GA	-0.100	0.097	-1.030	0.301	-0.289	0.089
HI	-0.570	0.208	-2.740	0.006	-0.979	-0.162
IA	-0.286	0.098	-2.920	0.003	-0.478	-0.094
ID	-0.356	0.108	-3.310	0.001	-0.567	-0.145
IL	-0.133	0.097	-1.370	0.169	-0.322	0.057
IN	-0.291	0.097	-2.990	0.003	-0.481	-0.100
KS	-0.233	0.099	-2.360	0.018	-0.427	-0.040
KY	-0.390	0.100	-3.910	0.000	-0.585	-0.194
LA	0.188	0.097	1.940	0.052	-0.002	0.377
MA	-0.076	0.099	-0.760	0.445	-0.270	0.118
MD	-0.226	0.097	-2.330	0.020	-0.416	-0.036
ME	-0.305	0.122	-2.500	0.012	-0.544	-0.066
MI	-0.013	0.098	-0.130	0.893	-0.205	0.179

© 2019 Jeffery MM et al. *JAMA Network Open.*

Outcome: any overlap days<br>Population: commercial, short-term episodes	Coef.	Robust Std. Err.	z	P>z	[95 Conf.	Interval]
MN	-0.652	0.097	-6.700	0.000	-0.842	-0.461
MO	-0.087	0.097	-0.900	0.371	-0.276	0.103
MS	-0.151	0.098	-1.540	0.125	-0.343	0.042
MT	-0.398	0.124	-3.200	0.001	-0.642	-0.154
NC	0.078	0.097	0.810	0.421	-0.111	0.267
ND	-0.450	0.114	-3.940	0.000	-0.673	-0.226
NE	-0.298	0.098	-3.040	0.002	-0.490	-0.106
NH	0.068	0.106	0.640	0.521	-0.140	0.277
NJ	-0.048	0.098	-0.490	0.624	-0.239	0.144
NM	-0.261	0.102	-2.550	0.011	-0.461	-0.061
NV	0.105	0.100	1.060	0.291	-0.090	0.301
NY	-0.201	0.097	-2.070	0.039	-0.392	-0.010
OH	-0.384	0.097	-3.970	0.000	-0.573	-0.195
OK	-0.222	0.098	-2.270	0.023	-0.413	-0.031
OR	-0.359	0.099	-3.640	0.000	-0.552	-0.166
PA	-0.139	0.098	-1.420	0.155	-0.330	0.052
RI	0.024	0.101	0.240	0.811	-0.174	0.222
SC	0.161	0.098	1.630	0.103	-0.032	0.353
SD	-0.434	0.130	-3.340	0.001	-0.689	-0.179
TN	-0.169	0.097	-1.730	0.083	-0.359	0.022
TX	-0.104	0.096	-1.080	0.279	-0.292	0.084
UT	-0.177	0.098	-1.810	0.071	-0.369	0.015
VA	-0.102	0.097	-1.050	0.293	-0.292	0.088
VT	-0.108	0.168	-0.640	0.521	-0.438	0.222
WA	-0.334	0.098	-3.420	0.001	-0.526	-0.143
WI	-0.285	0.097	-2.940	0.003	-0.474	-0.095
WV	-0.167	0.109	-1.530	0.125	-0.381	0.046
WY	-0.433	0.117	-3.700	0.000	-0.662	-0.203
Unknown/other	-0.086	0.102	-0.850	0.398	-0.287	0.114
race/ethnicity						

© 2019 Jeffery MM et al. *JAMA Network Open.*

Outcome: any overlap days<br>Population: commercial, short-term episodes	Coef.	Robust Std. Err.	z	P>z	[95 Conf.	Interval]
B	-0.467	0.009	-54.290	0.000	-0.484	-0.450
H	-0.383	0.008	-47.900	0.000	-0.399	-0.367
A	-0.633	0.015	-41.770	0.000	-0.662	-0.603
U	-0.096	0.011	-8.400	0.000	-0.118	-0.073
elix_chf	-0.023	0.026	-0.900	0.366	-0.074	0.027
elix_arrhythmia	0.107	0.015	7.190	0.000	0.078	0.137
elix_valvular	-0.045	0.025	-1.810	0.070	-0.095	0.004
elix_pulm_circ	0.017	0.035	0.500	0.619	-0.051	0.085
elix_periph_vasc	0.006	0.026	0.240	0.807	-0.045	0.058
elix_htn_uncomp	0.142	0.007	20.970	0.000	0.129	0.156
elix_htn_comp	0.035	0.029	1.210	0.227	-0.022	0.092
elix_oth_neuro	0.534	0.020	27.020	0.000	0.495	0.572
elix_chron_pulm	0.300	0.011	26.530	0.000	0.278	0.322
elix_pep_ulcer	0.192	0.057	3.400	0.001	0.081	0.303
elix_dm_wo_cc	-0.107	0.011	-9.770	0.000	-0.129	-0.086
elix_dm_w_cc	-0.122	0.017	-7.210	0.000	-0.156	-0.089
elix_paralysis	0.167	0.051	3.290	0.001	0.067	0.266
elix_renal	-0.118	0.027	-4.350	0.000	-0.172	-0.065
elix_solid_tumor_wo_mets	0.079	0.022	3.600	0.000	0.036	0.122
elix_liver	0.068	0.023	3.000	0.003	0.024	0.113
elix_met_cancer	0.000	0.085	0.000	0.997	-0.167	0.167
elix_hiv_aids	0.614	0.049	12.550	0.000	0.518	0.710
elix_rheum_arth_coll	0.136	0.017	8.010	0.000	0.103	0.170
elix_hypothyroid	0.166	0.012	14.390	0.000	0.144	0.189
elix_lymphoma	0.198	0.073	2.710	0.007	0.055	0.341
elix_coagulopathy	-0.078	0.029	-2.670	0.008	-0.135	-0.021
elix_obesity	-0.144	0.012	-12.050	0.000	-0.168	-0.121
elix_weight_loss	0.212	0.034	6.190	0.000	0.145	0.279
elix_fluid_electrolyte	0.125	0.016	7.630	0.000	0.093	0.157
elix_blood_loss_anemia	-0.227	0.049	-4.680	0.000	-0.322	-0.132

© 2019 Jeffery MM et al. *JAMA Network Open.*

Outcome: any overlap days Population: commercial, short-term episodes	Coef.	Robust Std. Err.	z	P>z	[95 Conf.	Interval]
elix_deficiency_anemia	0.106	0.025	4.220	0.000	0.056	0.155
elix_alcohol	0.142	0.026	5.500	0.000	0.092	0.193
elix_drug_abuse	0.518	0.027	19.370	0.000	0.466	0.571
elix_psychosis	0.649	0.039	16.580	0.000	0.573	0.726
elix_depression	1.319	0.008	169.150	0.000	1.303	1.334
less than 6 months prior insurance coverage	0.005	0.005	0.900	0.367	-0.006	0.015
female	0.435	0.005	91.930	0.000	0.426	0.445
age	0.018	0.000	107.230	0.000	0.017	0.018
_cons	-4.585	0.222	-20.680	0.000	-5.020	-4.151

Observations: 6,485,891 person-months contributed by 3,564,678 individuals

eTable 3. Regression Results: Extent of Coprescribing for the Commercially Insured—Long-term Opioid Use Episodes

Outcome: any overlap days Population: commercial, long-term episodes	Coef.	Robust Std. Err.	z	P>z	[95 Conf.	Interval]
Spline components						
months before release	0.003	0.001	2.010	0.044	0.000	0.005
months after release	-0.007	0.002	-3.780	0.000	-0.011	-0.004
binary post-release	0.051	0.017	2.950	0.003	0.017	0.085
state						
AL	0.968	0.469	2.060	0.039	0.048	1.887
AR	0.870	0.469	1.850	0.064	-0.050	1.789
AZ	0.532	0.465	1.140	0.252	-0.379	1.443
CA	0.861	0.464	1.850	0.064	-0.049	1.772
CO	0.723	0.465	1.550	0.120	-0.189	1.635
CT	1.065	0.488	2.180	0.029	0.108	2.022
DC	1.110	0.532	2.090	0.037	0.068	2.152

© 2019 Jeffery MM et al. *JAMA Network Open.*

Outcome: any overlap days Population: commercial, long-term episodes	Coef.	Robust Std. Err.	z	P>z	[95 Conf.	Interval]
DE	1.157	0.542	2.140	0.033	0.095	2.218
FL	1.011	0.463	2.180	0.029	0.103	1.919
GA	0.859	0.465	1.850	0.065	-0.053	1.770
HI	1.399	0.765	1.830	0.067	-0.100	2.897
IA	0.664	0.472	1.400	0.160	-0.262	1.590
ID	0.813	0.497	1.640	0.102	-0.161	1.786
IL	0.903	0.465	1.940	0.052	-0.009	1.815
IN	0.663	0.466	1.420	0.155	-0.250	1.575
KS	0.642	0.472	1.360	0.174	-0.283	1.568
KY	0.585	0.474	1.230	0.217	-0.344	1.513
LA	1.046	0.465	2.250	0.024	0.135	1.958
MA	0.909	0.487	1.870	0.062	-0.045	1.863
MD	0.584	0.468	1.250	0.213	-0.334	1.502
ME	0.918	0.574	1.600	0.110	-0.207	2.043
MI	0.874	0.468	1.870	0.062	-0.043	1.790
MN	0.364	0.469	0.780	0.437	-0.554	1.283
MO	0.982	0.465	2.110	0.035	0.071	1.893
MS	0.742	0.468	1.580	0.113	-0.177	1.660
MT	0.878	0.558	1.570	0.115	-0.215	1.971
NC	0.866	0.465	1.860	0.062	-0.045	1.776
ND	0.670	0.531	1.260	0.208	-0.372	1.711
NE	0.515	0.474	1.090	0.277	-0.413	1.444
NH	0.650	0.535	1.220	0.224	-0.397	1.698
NJ	1.023	0.474	2.160	0.031	0.094	1.953
NM	0.128	0.488	0.260	0.793	-0.829	1.085
NV	0.854	0.476	1.790	0.073	-0.079	1.788
NY	0.832	0.472	1.760	0.078	-0.093	1.757
OH	0.523	0.465	1.130	0.260	-0.387	1.434
OK	0.657	0.467	1.410	0.159	-0.258	1.573
OR	0.193	0.475	0.410	0.685	-0.739	1.124
PA	0.774	0.470	1.650	0.099	-0.147	1.696

© 2019 Jeffery MM et al. *JAMA Network Open.*

Outcome: any overlap days<br>Population: commercial, long-term episodes	Coef.	Robust Std. Err.	z	P>z	[95 Conf.	Interval]
RI	1.110	0.491	2.260	0.024	0.148	2.071
SC	0.904	0.471	1.920	0.055	-0.018	1.826
SD	-0.037	0.575	-0.070	0.948	-1.165	1.090
TN	0.717	0.467	1.540	0.125	-0.198	1.632
TX	0.763	0.463	1.650	0.100	-0.145	1.670
UT	0.821	0.471	1.740	0.081	-0.101	1.744
VA	0.862	0.469	1.840	0.066	-0.057	1.780
VT	1.499	0.804	1.860	0.062	-0.077	3.076
WA	0.439	0.471	0.930	0.351	-0.484	1.363
WI	0.604	0.467	1.300	0.195	-0.310	1.519
WV	0.501	0.510	0.980	0.326	-0.499	1.500
WY	0.287	0.585	0.490	0.624	-0.860	1.434
Unknown/other	0.863	0.482	1.790	0.073	-0.082	1.807
race/ethnicity						
B	-0.290	0.036	-8.080	0.000	-0.361	-0.220
H	-0.266	0.039	-6.800	0.000	-0.343	-0.189
A	-0.255	0.098	-2.610	0.009	-0.446	-0.064
U	-0.021	0.059	-0.350	0.723	-0.136	0.095
elix_chf	0.067	0.073	0.910	0.360	-0.077	0.211
elix_arrhythmia	0.084	0.044	1.890	0.058	-0.003	0.171
elix_valvular	-0.083	0.072	-1.160	0.247	-0.224	0.058
elix_pulm_circ	-0.066	0.092	-0.710	0.477	-0.246	0.115
elix_periph_vasc	-0.126	0.068	-1.840	0.066	-0.260	0.008
elix_htn_uncomp	0.127	0.021	6.190	0.000	0.087	0.168
elix_htn_comp	0.079	0.078	1.010	0.313	-0.074	0.232
elix_oth_neuro	0.521	0.057	9.190	0.000	0.410	0.632
elix_chron_pulm	0.227	0.035	6.560	0.000	0.159	0.295
elix_pep_ulcer	0.049	0.132	0.370	0.709	-0.210	0.308
elix_dm_wo_cc	-0.072	0.034	-2.150	0.032	-0.138	-0.006

© 2019 Jeffery MM et al. *JAMA Network Open.*

Outcome: any overlap days Population: commercial, long-term episodes	Coef.	Robust Std. Err.	z	P>z	[95 Conf.	Interval]
elix_dm_w_cc	-0.226	0.048	-4.700	0.000	-0.320	-0.132
elix_paralysis	-0.187	0.118	-1.580	0.114	-0.418	0.045
elix_renal	-0.087	0.077	-1.130	0.260	-0.238	0.064
elix_solid_tumor_wo_mets	-0.131	0.164	-0.800	0.426	-0.452	0.191
elix_liver	-0.022	0.062	-0.360	0.720	-0.144	0.100
elix_met_cancer	-1.363	0.502	-2.720	0.007	-2.347	-0.379
elix_hiv_aids	1.091	0.210	5.190	0.000	0.679	1.503
elix_rheum_arth_coll	-0.173	0.037	-4.620	0.000	-0.246	-0.100
elix_hypothyroid	0.145	0.038	3.790	0.000	0.070	0.220
elix_lymphoma	0.621	0.365	1.700	0.089	-0.095	1.336
elix_coagulopathy	0.006	0.083	0.080	0.940	-0.156	0.168
elix_obesity	-0.212	0.035	-6.060	0.000	-0.281	-0.143
elix_weight_loss	0.159	0.074	2.150	0.032	0.014	0.305
elix_fluid_electrolyte	0.157	0.042	3.740	0.000	0.075	0.239
elix_blood_loss_anemia	-0.163	0.142	-1.140	0.253	-0.442	0.116
elix_deficiency_anemia	0.102	0.074	1.380	0.169	-0.043	0.246
elix_alcohol	0.097	0.076	1.290	0.198	-0.051	0.245
elix_drug_abuse	0.155	0.043	3.640	0.000	0.072	0.239
elix_psychosis	0.637	0.126	5.060	0.000	0.390	0.884
elix_depression	1.033	0.025	41.220	0.000	0.984	1.082
less than 6 months prior insurance coverage	-0.047	0.024	-1.990	0.047	-0.093	-0.001
female	0.514	0.021	24.320	0.000	0.473	0.556
age	-0.005	0.001	-5.450	0.000	-0.007	-0.003
_cons	-3.863	0.976	-3.960	0.000	-5.776	-1.949

Observations: 734,545 person-months contributed by 59,423 individuals

© 2019 Jeffery MM et al. *JAMA Network Open.*

eTable 4. Regression Results: Extent of Coprescribing for Medicare Advantage—Short-term Episodes

Outcome: any overlap days Population: Medicare Advantage, short-term episodes	Coef.	Robust Std. Err.	z	P>z	[95 Conf.	Interval]
Spline components						
months before release	0.003	0.000	7.160	0.000	0.002	0.004
months after release	0.000	0.001	0.410	0.679	-0.001	0.002
binary post-release	-0.089	0.008	-10.490	0.000	-0.106	-0.072
state						
AL	-0.038	0.904	-0.040	0.966	-1.810	1.733
AR	-0.004	0.904	0.000	0.996	-1.776	1.768
AZ	-0.280	0.904	-0.310	0.757	-2.052	1.492
CA	-0.360	0.904	-0.400	0.691	-2.132	1.412
CO	-0.465	0.905	-0.510	0.608	-2.239	1.309
CT	-0.150	0.904	-0.170	0.868	-1.922	1.622
DC	-0.661	0.907	-0.730	0.466	-2.440	1.117
DE	-0.058	0.920	-0.060	0.950	-1.860	1.745
FL	0.161	0.904	0.180	0.859	-1.611	1.932
GA	-0.088	0.904	-0.100	0.922	-1.860	1.683
HI	-0.634	0.905	-0.700	0.484	-2.408	1.140
IA	-0.326	0.904	-0.360	0.719	-2.098	1.447
ID	-0.606	0.905	-0.670	0.503	-2.380	1.167
IL	-0.204	0.904	-0.230	0.822	-1.975	1.568
IN	-0.407	0.904	-0.450	0.652	-2.179	1.365
KS	-0.144	0.906	-0.160	0.873	-1.920	1.632
KY	-0.189	0.906	-0.210	0.835	-1.965	1.587
LA	0.287	0.908	0.320	0.752	-1.492	2.066
MA	-0.196	0.904	-0.220	0.829	-1.968	1.577
MD	-0.437	0.907	-0.480	0.630	-2.215	1.340
ME	-0.340	0.906	-0.380	0.707	-2.115	1.435

© 2019 Jeffery MM et al. *JAMA Network Open.*

Outcome: any overlap days Population: Medicare Advantage, short-term episodes	Coef.	Robust Std. Err.	z	P>z	[95 Conf.	Interval]
MI	-0.411	0.911	-0.450	0.652	-2.198	1.375
MN	-0.724	0.904	-0.800	0.423	-2.496	1.048
MO	-0.106	0.904	-0.120	0.907	-1.877	1.666
MS	-0.196	0.920	-0.210	0.832	-2.000	1.608
MT	-0.500	0.919	-0.540	0.586	-2.300	1.301
NC	0.073	0.904	0.080	0.936	-1.699	1.844
ND	-0.549	0.907	-0.610	0.545	-2.326	1.228
NE	-0.215	0.905	-0.240	0.812	-1.988	1.558
NH	-0.097	0.906	-0.110	0.915	-1.873	1.679
NJ	-0.173	0.904	-0.190	0.848	-1.945	1.599
NM	-0.385	0.904	-0.430	0.670	-2.158	1.388
NV	-0.290	0.917	-0.320	0.752	-2.087	1.507
NY	-0.524	0.904	-0.580	0.562	-2.296	1.247
OH	-0.247	0.904	-0.270	0.785	-2.019	1.525
OK	0.023	0.917	0.020	0.980	-1.775	1.820
OR	-0.631	0.905	-0.700	0.486	-2.404	1.142
PA	-0.110	0.905	-0.120	0.903	-1.883	1.663
RI	-0.053	0.904	-0.060	0.953	-1.826	1.719
SC	0.000	0.904	0.000	1.000	-1.771	1.772
SD	-0.543	0.907	-0.600	0.549	-2.321	1.234
TN	0.002	0.904	0.000	0.998	-1.770	1.774
TX	-0.399	0.904	-0.440	0.659	-2.171	1.372
UT	-0.311	0.904	-0.340	0.731	-2.082	1.461
VA	0.030	0.904	0.030	0.973	-1.742	1.802
VT	-0.479	0.907	-0.530	0.598	-2.257	1.300
WA	-0.642	0.904	-0.710	0.478	-2.414	1.130
WI	-0.343	0.904	-0.380	0.705	-2.114	1.429
WV	0.034	0.929	0.040	0.971	-1.788	1.856
WY	-0.710	0.916	-0.780	0.438	-2.506	1.086
Unknown/other	-0.250	0.907	-0.280	0.782	-2.028	1.527

© 2019 Jeffery MM et al. *JAMA Network Open.*

Outcome: any overlap days Population: Medicare Advantage, short-term episodes	Coef.	Robust Std. Err.	z	P>z	[95 Conf.	Interval]
race/ethnicity						
B	-0.528	0.010	-51.320	0.000	-0.548	-0.508
H	0.087	0.012	7.500	0.000	0.064	0.110
A	-0.549	0.030	-18.050	0.000	-0.608	-0.489
U	-0.065	0.015	-4.450	0.000	-0.094	-0.036
elix_chf	0.060	0.012	4.940	0.000	0.036	0.083
elix_arrhythmia	0.018	0.010	1.860	0.063	-0.001	0.037
elix_valvular	-0.053	0.015	-3.660	0.000	-0.082	-0.025
elix_pulm_circ	0.034	0.021	1.620	0.105	-0.007	0.075
elix_periph_vasc	-0.011	0.012	-0.860	0.391	-0.035	0.014
elix_htn_uncomp	0.007	0.006	1.290	0.198	-0.004	0.019
elix_htn_comp	-0.027	0.014	-1.870	0.061	-0.054	0.001
elix_oth_neuro	0.286	0.013	21.930	0.000	0.260	0.312
elix_chron_pulm	0.331	0.008	39.410	0.000	0.315	0.348
elix_pep_ulcer	-0.016	0.041	-0.390	0.700	-0.097	0.065
elix_dm_wo_cc	-0.107	0.008	-13.360	0.000	-0.123	-0.091
elix_dm_w_cc	-0.173	0.010	-17.010	0.000	-0.193	-0.153
elix_paralysis	-0.087	0.032	-2.710	0.007	-0.150	-0.024
elix_renal	-0.080	0.013	-6.370	0.000	-0.105	-0.055
elix_solid_tumor_wo_mets	-0.078	0.018	-4.250	0.000	-0.114	-0.042
elix_liver	0.003	0.021	0.130	0.896	-0.038	0.044
elix_met_cancer	-0.031	0.074	-0.420	0.678	-0.175	0.114
elix_hiv_aids	0.077	0.060	1.270	0.205	-0.042	0.195
elix_rheum_arth_coll	-0.081	0.016	-5.170	0.000	-0.112	-0.051
elix_hypothyroid	0.126	0.010	12.420	0.000	0.106	0.145
elix_lymphoma	-0.036	0.062	-0.580	0.563	-0.157	0.085
elix_coagulopathy	-0.072	0.023	-3.170	0.002	-0.117	-0.028
elix_obesity	-0.238	0.014	-17.100	0.000	-0.265	-0.210

© 2019 Jeffery MM et al. *JAMA Network Open.*

Outcome: any overlap days Population: Medicare Advantage, short-term episodes	Coef.	Robust Std. Err.	z	P>z	[95 Conf.	Interval]
elix_weight_loss	0.127	0.020	6.390	0.000	0.088	0.166
elix_fluid_electrolyte	0.064	0.012	5.570	0.000	0.042	0.087
elix_blood_loss_anemia	-0.049	0.034	-1.450	0.147	-0.116	0.017
elix_deficiency_anemia	0.084	0.018	4.700	0.000	0.049	0.119
elix_alcohol	-0.024	0.029	-0.830	0.409	-0.080	0.032
elix_drug_abuse	0.409	0.026	15.750	0.000	0.358	0.459
elix_psychosis	0.544	0.020	27.410	0.000	0.505	0.583
elix_depression	0.968	0.009	109.920	0.000	0.950	0.985
less than 6 months prior insurance coverage	-0.023	0.007	-3.170	0.002	-0.037	-0.009
female	0.454	0.007	65.520	0.000	0.440	0.467
age	-0.019	0.000	-58.930	0.000	-0.019	-0.018
_cons	-2.761	0.949	-2.910	0.004	-4.621	-0.901

Observations: 3,255,263 person-months contributed by 1,262,727 individuals

eTable 5. Regression Results: Extent of Coprescribing for Medicare Advantage—Long-term Episodes

Outcome: any overlap days Population: Medicare Advantage, long-term episodes	Coef.	Robust Std. Err.	z	P>z	[95 Conf.	Interval]
Spline components						
months before release	0.005	0.001	4.000	0.000	0.003	0.008
months after release	-0.010	0.002	-5.690	0.000	-0.013	-0.007
binary post-release	-0.008	0.016	-0.460	0.644	-0.039	0.024
state						
AL	-2.866	0.054	-52.930	0.000	-2.972	-2.759

© 2019 Jeffery MM et al. *JAMA Network Open.*

Outcome: any overlap days Population: Medicare Advantage, long-term episodes	Coef.	Robust Std. Err.	z	P>z	[95 Conf.	Interval]
AR	-2.957	0.076	-38.850	0.000	-3.107	-2.808
AZ	-3.305	0.092	-35.740	0.000	-3.487	-3.124
CA	-3.208	0.104	-30.940	0.000	-3.411	-3.004
CO	-3.588	0.117	-30.770	0.000	-3.817	-3.359
CT	-3.069	0.084	-36.320	0.000	-3.234	-2.903
DC	-3.885	0.260	-14.920	0.000	-4.395	-3.374
DE	-3.001	0.331	-9.070	0.000	-3.650	-2.353
FL	-2.759	0.031	-89.320	0.000	-2.819	-2.698
GA	-3.053	0.043	-70.670	0.000	-3.138	-2.969
HI	-3.148	0.163	-19.330	0.000	-3.467	-2.829
IA	-3.181	0.111	-28.540	0.000	-3.399	-2.962
ID	-3.344	0.134	-25.000	0.000	-3.606	-3.082
IL	-3.115	0.055	-56.540	0.000	-3.223	-3.007
IN	-3.266	0.058	-56.360	0.000	-3.379	-3.152
KS	-3.116	0.185	-16.810	0.000	-3.479	-2.752
KY	-3.010	0.175	-17.170	0.000	-3.353	-2.666
LA	-3.404	0.333	-10.210	0.000	-4.058	-2.750
MA	-3.090	0.103	-30.050	0.000	-3.291	-2.888
MD	-3.682	0.205	-17.920	0.000	-4.085	-3.279
ME	-3.343	0.189	-17.680	0.000	-3.714	-2.972
MI	-3.359	0.383	-8.760	0.000	-4.111	-2.608
MN	-3.642	0.085	-42.680	0.000	-3.809	-3.475
MO	-2.975	0.051	-58.830	0.000	-3.074	-2.876
MS	-2.304	0.424	-5.440	0.000	-3.135	-1.474
MT	-3.448	0.499	-6.900	0.000	-4.427	-2.470
NC	-2.769	0.037	-74.410	0.000	-2.842	-2.696
ND	-3.378	0.242	-13.980	0.000	-3.851	-2.904
NE	-3.164	0.116	-27.220	0.000	-3.392	-2.936
NH	-3.096	0.189	-16.350	0.000	-3.467	-2.725
NJ	-2.948	0.070	-42.200	0.000	-3.085	-2.811

© 2019 Jeffery MM et al. *JAMA Network Open.*

Outcome: any overlap days Population: Medicare Advantage, long-term episodes	Coef.	Robust Std. Err.	z	P>z	[95 Conf.	Interval]
NM	-3.321	0.101	-32.960	0.000	-3.519	-3.124
NV	-2.951	0.418	-7.050	0.000	-3.771	-2.131
NY	-3.352	0.048	-69.690	0.000	-3.446	-3.257
OH	-3.147	0.054	-58.760	0.000	-3.252	-3.042
OK	-3.522	0.456	-7.720	0.000	-4.416	-2.628
OR	-3.638	0.133	-27.390	0.000	-3.898	-3.378
PA	-3.142	0.104	-30.110	0.000	-3.346	-2.937
RI	-3.335	0.100	-33.210	0.000	-3.532	-3.138
SC	-2.873	0.058	-49.170	0.000	-2.987	-2.758
SD	-2.976	0.202	-14.750	0.000	-3.371	-2.580
TN	-2.987	0.063	-47.600	0.000	-3.110	-2.864
TX	-3.116	0.049	-63.590	0.000	-3.212	-3.020
UT	-3.137	0.069	-45.280	0.000	-3.273	-3.001
VA	-2.827	0.072	-39.320	0.000	-2.968	-2.686
VT	-3.757	0.268	-14.040	0.000	-4.281	-3.232
WA	-3.547	0.085	-41.840	0.000	-3.713	-3.381
WI	-3.257	0.049	-66.000	0.000	-3.353	-3.160
WV	-3.324	0.668	-4.980	0.000	-4.632	-2.015
WY	-3.551	0.397	-8.940	0.000	-4.329	-2.772
Unknown/other	-3.041	0.171	-17.830	0.000	-3.375	-2.707
race/ethnicity						
B	-0.497	0.029	-17.180	0.000	-0.554	-0.440
H	-0.007	0.037	-0.180	0.853	-0.079	0.066
A	-0.469	0.105	-4.450	0.000	-0.676	-0.262
U	-0.089	0.050	-1.790	0.074	-0.187	0.008
elix_chf	0.016	0.030	0.530	0.594	-0.043	0.075
elix_arrhythmia	0.020	0.027	0.760	0.446	-0.032	0.073
elix_valvular	-0.007	0.041	-0.170	0.864	-0.087	0.073

© 2019 Jeffery MM et al. *JAMA Network Open.*

Outcome: any overlap days Population: Medicare Advantage, long-term episodes	Coef.	Robust Std. Err.	z	P>z	[95 Conf.	Interval]
elix_pulm_circ	-0.090	0.053	-1.700	0.089	-0.194	0.014
elix_periph_vasc	-0.083	0.030	-2.800	0.005	-0.140	-0.025
elix_htn_uncomp	0.003	0.015	0.160	0.870	-0.028	0.033
elix_htn_comp	-0.007	0.036	-0.180	0.854	-0.077	0.064
elix_oth_neuro	0.226	0.033	6.820	0.000	0.161	0.291
elix_chron_pulm	0.329	0.021	15.440	0.000	0.288	0.371
elix_pep_ulcer	-0.013	0.081	-0.160	0.872	-0.171	0.145
elix_dm_wo_cc	-0.135	0.022	-6.210	0.000	-0.178	-0.093
elix_dm_w_cc	-0.240	0.026	-9.250	0.000	-0.291	-0.189
elix_paralysis	0.042	0.071	0.600	0.551	-0.097	0.181
elix_renal	-0.131	0.032	-4.110	0.000	-0.193	-0.069
elix_solid_tumor_wo_mets	-0.030	0.089	-0.330	0.740	-0.204	0.145
elix_liver	0.123	0.048	2.580	0.010	0.029	0.217
elix_met_cancer	-0.100	0.272	-0.370	0.712	-0.633	0.432
elix_hiv_aids	0.304	0.156	1.950	0.051	-0.001	0.609
elix_rheum_arth_coll	-0.189	0.033	-5.720	0.000	-0.254	-0.124
elix_hypothyroid	0.130	0.028	4.740	0.000	0.077	0.184
elix_lymphoma	0.228	0.242	0.940	0.345	-0.246	0.703
elix_coagulopathy	-0.040	0.058	-0.680	0.494	-0.153	0.074
elix_obesity	-0.201	0.034	-5.960	0.000	-0.267	-0.135
elix_weight_loss	-0.009	0.042	-0.210	0.830	-0.091	0.073
elix_fluid_electrolyte	0.109	0.026	4.170	0.000	0.058	0.160
elix_blood_loss_anemia	-0.036	0.077	-0.470	0.642	-0.188	0.116
elix_deficiency_anemia	0.008	0.043	0.190	0.850	-0.076	0.092
elix_alcohol	0.013	0.066	0.190	0.846	-0.117	0.142
elix_drug_abuse	0.154	0.041	3.800	0.000	0.075	0.234
elix_psychosis	0.460	0.047	9.820	0.000	0.368	0.552
elix_depression	0.760	0.021	35.610	0.000	0.718	0.802
less than 6 months prior insurance coverage	-0.099	0.025	-4.020	0.000	-0.148	-0.051

© 2019 Jeffery MM et al. *JAMA Network Open.*

Outcome: any overlap days Population: Medicare Advantage, long-term episodes	Coef.	Robust Std. Err.	z	P>z	[95 Conf.	Interval]
female	0.388	0.021	18.440	0.000	0.346	0.429
age	-0.027	0.001	-31.030	0.000	-0.029	-0.025
_cons	0.153	0.837	0.180	0.855	-1.488	1.793

Observations: 889,459 person-months contributed by 69,153 individuals

eTable 6. Adjusted Estimates: Extent and Intensity of Coprescription

	Population	Commercially insured		Medicare Advantage	
	Opioid episode type	Long-term	Short-term	Long-term	Short-term
		Value (95% CI)	Value (95% CI)	Value (95% CI)	Value (95% CI)
		**Extent of coprescription: Adjusted percent of overlapping opioids and BZDs in month**			
Adjusted percent of people with overlapping opioids and BZDs in month	Study start	22.07 (21.26 to 22.87)	8.83 (8.75 to 8.90)	24.42 (23.56 to 25.28)	13.52 (13.36 to 13.69)
	Just before guideline	23.20 (22.62 to 23.78)	9.24 (9.16 to 9.31)	26.78 (26.16 to 27.41)	14.47 (14.31 to 14.63)
	Just after guideline	24.08 (23.49 to 24.66)	8.98 (8.90 to 9.07)	26.65 (26.06 to 27.24)	13.45 (13.30 to 13.60)
	Study end	22.18 (21.49 to 22.86)	8.88 (8.79 to 8.97)	24.53 (23.98 to 25.09)	14.39 (14.24 to 14.54)
		**Intensity of coprescription: Adjusted percent of opioid days with concurrent BZDs**			
Adjusted percent of opioid days that also have BZDs available	Study start	78.36 (77.53 to 79.18)	79.03 (78.77 to 79.29)	82.62 (81.90 to 83.35)	82.62 (81.90 to 83.35)
	Just before guideline	79.60 (79.02 to 80.17)	79.44 (79.19 to 79.69)	83.83 (83.33 to 84.32)	83.83 (83.33 to 84.32)
	Just after guideline	79.51 (78.94 to 80.09)	79.13 (78.85 to 79.42)	83.50 (83.04 to 83.96)	83.50 (83.04 to 83.96)
	Study end	80.05 (79.33 to 80.77)	79.73 (79.41 to 80.04)	85.18 (84.74 to 85.62)	85.18 (84.74 to 85.62)

Adjusted percentages represent predictive margins from a logit or zero-truncated negative binomial model that included patient age, sex, race/ethnicity, state of residence, and Elixhauser comorbidity flags calculated on a rolling 6 month basis. Separate models were specified for each patient population and episode type (commercial and Medicare Advantage; long-term and short-term). Standard errors in the model were adjusted for clustering on individual patient.
BZD=benzodiazepine

© 2019 Jeffery MM et al. *JAMA Network Open.*

eTable 7. Regression Results: Intensity of Coprescribing for the Commercially Insured—Short-term Episodes

Outcome: number of overlap days Population: commercial, short-term episodes	Coef.	Robust Std. Err.	z	P>z	[95 Conf.	Interval]
Spline components						
months before release	0.000	0.000	1.810	0.071	0.000	0.000
months after release	0.000	0.000	0.620	0.535	0.000	0.000
binary post-release	-0.004	0.002	-1.600	0.110	-0.009	0.001
state						
AL	0.081	0.028	2.860	0.004	0.025	0.137
AR	0.053	0.028	1.890	0.059	-0.002	0.109
AZ	0.006	0.028	0.230	0.817	-0.048	0.061
CA	0.008	0.028	0.300	0.764	-0.046	0.063
CO	-0.015	0.028	-0.520	0.601	-0.070	0.040
CT	0.031	0.029	1.070	0.285	-0.026	0.088
DC	-0.022	0.032	-0.690	0.488	-0.086	0.041
DE	0.002	0.035	0.070	0.944	-0.067	0.071
FL	0.036	0.028	1.310	0.191	-0.018	0.091
GA	0.006	0.028	0.220	0.823	-0.049	0.061
HI	0.143	0.047	3.040	0.002	0.051	0.235
IA	0.008	0.028	0.280	0.777	-0.048	0.064
ID	-0.046	0.033	-1.400	0.161	-0.111	0.018
IL	0.026	0.028	0.920	0.358	-0.029	0.081
IN	0.028	0.028	0.980	0.328	-0.028	0.083
KS	0.037	0.029	1.300	0.192	-0.019	0.093
KY	0.032	0.029	1.090	0.275	-0.025	0.088
LA	0.034	0.028	1.230	0.220	-0.021	0.089
MA	0.023	0.029	0.780	0.434	-0.034	0.079
MD	0.019	0.028	0.690	0.491	-0.036	0.075
ME	0.008	0.039	0.210	0.832	-0.068	0.084
MI	0.044	0.028	1.540	0.123	-0.012	0.099
MN	-0.032	0.028	-1.140	0.256	-0.088	0.023
MO	0.039	0.028	1.410	0.160	-0.016	0.094
MS	0.050	0.028	1.760	0.079	-0.006	0.106
MT	0.018	0.038	0.490	0.626	-0.055	0.092
NC	0.013	0.028	0.480	0.633	-0.041	0.068
ND	-0.034	0.037	-0.920	0.358	-0.107	0.039
NE	-0.005	0.029	-0.190	0.850	-0.061	0.051
NH	0.003	0.032	0.100	0.917	-0.059	0.066
NJ	0.045	0.028	1.570	0.116	-0.011	0.100
NM	0.019	0.030	0.630	0.531	-0.040	0.078

© 2019 Jeffery MM et al. *JAMA Network Open.*

Outcome: number of overlap days<br>Population: commercial, short-term episodes	Coef.	Robust Std. Err.	z	P>z	[95 Conf.	Interval]
NV	0.041	0.029	1.420	0.154	-0.015	0.098
NY	0.044	0.028	1.550	0.122	-0.012	0.099
OH	0.015	0.028	0.550	0.583	-0.040	0.070
OK	0.018	0.028	0.640	0.523	-0.037	0.074
OR	-0.086	0.030	-2.890	0.004	-0.144	-0.028
PA	0.035	0.028	1.250	0.213	-0.020	0.091
RI	0.033	0.029	1.130	0.260	-0.025	0.091
SC	0.055	0.028	1.950	0.051	0.000	0.111
SD	-0.012	0.041	-0.300	0.767	-0.092	0.068
TN	0.047	0.028	1.670	0.095	-0.008	0.102
TX	0.010	0.028	0.350	0.730	-0.045	0.064
UT	0.001	0.029	0.040	0.970	-0.055	0.057
VA	0.008	0.028	0.290	0.773	-0.047	0.063
VT	-0.023	0.058	-0.390	0.695	-0.136	0.090
WA	-0.056	0.029	-1.950	0.051	-0.113	0.000
WI	-0.003	0.028	-0.110	0.909	-0.058	0.052
WV	0.054	0.031	1.760	0.078	-0.006	0.115
WY	-0.037	0.037	-1.000	0.315	-0.110	0.035
Unknown/other	0.033	0.031	1.080	0.279	-0.027	0.093
race/ethnicity						
B	-0.014	0.003	-5.380	0.000	-0.020	-0.009
H	-0.005	0.003	-1.910	0.056	-0.010	0.000
A	-0.032	0.006	-5.620	0.000	-0.043	-0.021
U	-0.002	0.004	-0.570	0.569	-0.009	0.005
elix_chf	0.002	0.007	0.230	0.818	-0.012	0.015
elix_arrhythmia	-0.009	0.004	-2.090	0.036	-0.018	-0.001
elix_valvular	-0.009	0.007	-1.260	0.206	-0.023	0.005
elix_pulm_circ	-0.005	0.010	-0.560	0.578	-0.024	0.014
elix_periph_vasc	-0.011	0.007	-1.490	0.137	-0.025	0.003
elix_htn_uncomp	-0.001	0.002	-0.350	0.723	-0.005	0.003
elix_htn_comp	0.001	0.008	0.120	0.906	-0.015	0.017
elix_oth_neuro	0.030	0.005	6.290	0.000	0.021	0.040
elix_chron_pulm	0.012	0.003	3.920	0.000	0.006	0.018
elix_pep_ulcer	0.004	0.015	0.270	0.790	-0.025	0.033
elix_dm_wo_cc	-0.008	0.003	-2.490	0.013	-0.015	-0.002
elix_dm_w_cc	0.001	0.005	0.180	0.860	-0.009	0.011
elix_paralysis	-0.003	0.012	-0.250	0.800	-0.026	0.020
elix_renal	0.006	0.007	0.760	0.447	-0.009	0.020
elix_solid_tumor_wo_mets	0.010	0.007	1.440	0.149	-0.004	0.024

© 2019 Jeffery MM et al. *JAMA Network Open.*

Outcome: number of overlap days<br>Population: commercial, short-term episodes	Coef.	Robust Std. Err.	z	P>z	[95 Conf.	Interval]
elix_liver	0.006	0.006	0.940	0.349	-0.007	0.019
elix_met_cancer	-0.054	0.030	-1.780	0.075	-0.113	0.005
elix_hiv_aids	0.053	0.013	4.040	0.000	0.027	0.079
elix_rheum_arth_coll	-0.019	0.005	-4.200	0.000	-0.028	-0.010
elix_hypothyroid	0.002	0.003	0.480	0.630	-0.005	0.008
elix_lymphoma	0.021	0.020	1.010	0.313	-0.019	0.061
elix_coagulopathy	-0.009	0.009	-1.010	0.313	-0.026	0.008
elix_obesity	-0.026	0.004	-6.560	0.000	-0.034	-0.018
elix_weight_loss	-0.013	0.009	-1.430	0.154	-0.032	0.005
elix_fluid_electrolyte	-0.018	0.005	-3.780	0.000	-0.028	-0.009
elix_blood_loss_anemia	-0.023	0.015	-1.530	0.126	-0.052	0.006
elix_deficiency_anemia	0.019	0.007	2.750	0.006	0.006	0.033
elix_alcohol	-0.005	0.007	-0.830	0.408	-0.018	0.008
elix_drug_abuse	0.045	0.006	7.360	0.000	0.033	0.057
elix_psychosis	0.056	0.007	7.970	0.000	0.043	0.070
elix_depression	0.066	0.002	34.690	0.000	0.062	0.070
less than 6 months prior insurance coverage	0.011	0.002	5.700	0.000	0.007	0.015
female	0.020	0.002	13.500	0.000	0.017	0.023
age	0.002	0.000	38.120	0.000	0.002	0.002
_cons	-0.511	0.078	-6.550	0.000	-0.663	-0.358
ln(op_days)	1.000	(exposure)				
/lnalpha	-2.681	0.010			-2.700	-2.662
alpha	0.069	0.001			0.067	0.070

Observations: 583,020 person-months contributed by 344,179 individuals

eTable 8. Regression Results: Intensity of Coprescribing for the Commercially Insured—Long-term Episodes

Outcome: number of overlap days<br>Population: commercial, long-term episodes	Coef.	Robust Std. Err.	z	P>z	[95 Conf.	Interval]
Spline components						
months before release	0.001	0.000	2.100	0.036	0.000	0.001
months after release	0.000	0.000	-0.770	0.444	-0.001	0.001
binary post-release	-0.001	0.004	-0.250	0.801	-0.009	0.007
state						
AL	0.327	0.127	2.570	0.010	0.077	0.576

Outcome: number of overlap days<br>Population: commercial, long-term episodes	Coef.	Robust Std. Err.	z	P>z	[95 Conf.	Interval]
AR	0.298	0.127	2.340	0.019	0.048	0.547
AZ	0.230	0.127	1.810	0.071	-0.019	0.479
CA	0.253	0.127	1.990	0.047	0.004	0.502
CO	0.216	0.127	1.700	0.090	-0.033	0.465
CT	0.231	0.130	1.780	0.076	-0.024	0.486
DC	0.237	0.141	1.690	0.091	-0.038	0.513
DE	0.251	0.137	1.830	0.067	-0.017	0.520
FL	0.299	0.127	2.360	0.018	0.051	0.547
GA	0.268	0.127	2.110	0.035	0.019	0.517
HI	0.300	0.154	1.950	0.052	-0.002	0.603
IA	0.262	0.129	2.040	0.041	0.011	0.514
ID	0.265	0.131	2.030	0.043	0.009	0.522
IL	0.293	0.127	2.310	0.021	0.044	0.542
IN	0.292	0.127	2.300	0.021	0.043	0.541
KS	0.257	0.128	2.010	0.045	0.006	0.508
KY	0.331	0.128	2.600	0.009	0.081	0.581
LA	0.299	0.127	2.360	0.018	0.050	0.548
MA	0.265	0.130	2.040	0.042	0.010	0.521
MD	0.268	0.128	2.100	0.036	0.018	0.518
ME	0.175	0.168	1.040	0.297	-0.154	0.505
MI	0.292	0.127	2.290	0.022	0.042	0.541
MN	0.206	0.128	1.610	0.107	-0.045	0.457
MO	0.274	0.127	2.160	0.031	0.025	0.523
MS	0.309	0.127	2.430	0.015	0.060	0.559
MT	0.276	0.144	1.920	0.055	-0.006	0.557
NC	0.283	0.127	2.230	0.026	0.034	0.531
ND	0.280	0.133	2.110	0.035	0.020	0.540
NE	0.266	0.128	2.080	0.038	0.015	0.518
NH	0.189	0.146	1.300	0.195	-0.097	0.474
NJ	0.277	0.128	2.160	0.031	0.026	0.528
NM	0.258	0.130	1.980	0.047	0.003	0.513
NV	0.293	0.129	2.280	0.023	0.041	0.545
NY	0.266	0.128	2.090	0.037	0.016	0.517
OH	0.275	0.127	2.160	0.031	0.026	0.523
OK	0.290	0.127	2.280	0.022	0.041	0.540
OR	0.000	0.138	0.000	0.999	-0.270	0.270
PA	0.295	0.127	2.320	0.020	0.046	0.545
RI	0.262	0.130	2.020	0.043	0.008	0.517
SC	0.269	0.128	2.110	0.035	0.019	0.520
SD	0.177	0.173	1.020	0.305	-0.161	0.515
TN	0.297	0.127	2.330	0.020	0.048	0.546

© 2019 Jeffery MM et al. *JAMA Network Open.*

Outcome: number of overlap days<br>Population: commercial, long-term episodes	Coef.	Robust Std. Err.	z	P>z	[95 Conf.	Interval]
TX	0.257	0.127	2.030	0.043	0.009	0.505
UT	0.236	0.129	1.840	0.066	-0.016	0.488
VA	0.220	0.128	1.720	0.086	-0.031	0.470
VT	0.366	0.132	2.760	0.006	0.107	0.626
WA	0.157	0.129	1.220	0.222	-0.095	0.410
WI	0.249	0.127	1.950	0.051	-0.001	0.498
WV	0.297	0.134	2.220	0.026	0.035	0.560
WY	0.267	0.148	1.800	0.072	-0.024	0.558
Unknown/other	0.277	0.129	2.150	0.032	0.024	0.530
race/ethnicity						
B	-0.016	0.007	-2.290	0.022	-0.030	-0.002
H	-0.009	0.008	-1.110	0.268	-0.024	0.007
A	-0.024	0.024	-1.000	0.315	-0.072	0.023
U	-0.007	0.011	-0.680	0.499	-0.029	0.014
elix_chf	-0.024	0.014	-1.720	0.086	-0.052	0.003
elix_arrhythmia	0.008	0.008	0.920	0.358	-0.009	0.024
elix_valvular	-0.008	0.014	-0.580	0.561	-0.037	0.020
elix_pulm_circ	-0.017	0.021	-0.800	0.426	-0.058	0.024
elix_periph_vasc	-0.042	0.015	-2.900	0.004	-0.070	-0.014
elix_htn_uncomp	-0.005	0.004	-1.270	0.204	-0.013	0.003
elix_htn_comp	0.006	0.015	0.410	0.679	-0.023	0.035
elix_oth_neuro	0.017	0.009	1.980	0.048	0.000	0.034
elix_chron_pulm	0.008	0.006	1.230	0.217	-0.005	0.020
elix_pep_ulcer	-0.004	0.023	-0.170	0.866	-0.049	0.041
elix_dm_wo_cc	-0.008	0.007	-1.180	0.239	-0.021	0.005
elix_dm_w_cc	-0.013	0.010	-1.380	0.167	-0.032	0.006
elix_paralysis	-0.030	0.021	-1.400	0.162	-0.072	0.012
elix_renal	0.017	0.015	1.100	0.272	-0.013	0.046
elix_solid_tumor_wo_mets	-0.043	0.032	-1.350	0.178	-0.106	0.020
elix_liver	0.002	0.011	0.180	0.855	-0.020	0.024
elix_met_cancer	-0.413	0.255	-1.620	0.106	-0.913	0.088
elix_hiv_aids	0.058	0.026	2.190	0.028	0.006	0.109
elix_rheum_arth_coll	-0.027	0.008	-3.430	0.001	-0.042	-0.011
elix_hypothyroid	-0.013	0.008	-1.630	0.102	-0.028	0.003
elix_lymphoma	-0.082	0.113	-0.720	0.472	-0.304	0.141
elix_coagulopathy	0.008	0.015	0.510	0.608	-0.022	0.037
elix_obesity	-0.018	0.008	-2.370	0.018	-0.033	-0.003
elix_weight_loss	-0.018	0.015	-1.210	0.225	-0.048	0.011
elix_fluid_electrolyte	-0.010	0.008	-1.250	0.210	-0.026	0.006

© 2019 Jeffery MM et al. *JAMA Network Open.*

Outcome: number of overlap days<br>Population: commercial, long-term episodes	Coef.	Robust Std. Err.	z	P>z	[95 Conf.	Interval]
elix_blood_loss_anemia	0.000	0.027	-0.020	0.986	-0.053	0.053
elix_deficiency_anemia	0.028	0.013	2.140	0.032	0.002	0.053
elix_alcohol	-0.007	0.014	-0.540	0.590	-0.035	0.020
elix_drug_abuse	-0.015	0.009	-1.730	0.083	-0.032	0.002
elix_psychosis	0.054	0.018	3.010	0.003	0.019	0.089
elix_depression	0.040	0.004	9.680	0.000	0.032	0.048
less than 6 months prior insurance coverage	0.003	0.006	0.510	0.613	-0.008	0.014
female	-0.009	0.004	-2.150	0.032	-0.017	-0.001
age	0.001	0.000	6.400	0.000	0.001	0.002
_cons	-0.957	0.229	-4.190	0.000	-1.405	-0.509
ln(op_days)	1.000	(exposure)				
/lnalpha	-1.923	0.015			-1.953	-1.894
alpha	0.146	0.002			0.142	0.150
Observations: 168,810 person-months contributed by 22,290 individuals						

eTable 9. Regression Results: Intensity of Coprescribing for Medicare Advantage—Short-term Episodes

Outcome: number of overlap days<br>Population: Medicare Advantage, short-term episodes	Coef.	Robust Std. Err.	z	P>z	[95 Conf.	Interval]
Spline components						
months before release	0.000	0.000	2.230	0.026	0.000	0.001
months after release	0.000	0.000	0.380	0.706	0.000	0.000
binary post-release	-0.002	0.002	-0.820	0.409	-0.007	0.003
state						
AL	-0.084	0.006	-13.690	0.000	-0.096	-0.072
AR	-0.081	0.007	-11.270	0.000	-0.095	-0.067
AZ	-0.105	0.008	-12.900	0.000	-0.122	-0.089
CA	-0.141	0.009	-16.340	0.000	-0.158	-0.124
CO	-0.133	0.012	-11.490	0.000	-0.155	-0.110
CT	-0.112	0.007	-15.000	0.000	-0.126	-0.097
DC	-0.114	0.021	-5.520	0.000	-0.154	-0.073
DE	-0.195	0.049	-4.010	0.000	-0.291	-0.100
FL	-0.087	0.005	-16.770	0.000	-0.097	-0.077
GA	-0.115	0.006	-20.380	0.000	-0.126	-0.104
HI	-0.107	0.012	-8.960	0.000	-0.130	-0.083
IA	-0.118	0.009	-12.570	0.000	-0.137	-0.100

© 2019 Jeffery MM et al. *JAMA Network Open.*

Outcome: number of overlap days<br>Population: Medicare Advantage, short-term episodes	Coef.	Robust Std. Err.	z	P>z	[95 Conf.	Interval]
ID	-0.142	0.012	-11.500	0.000	-0.166	-0.118
IL	-0.109	0.006	-18.350	0.000	-0.121	-0.097
IN	-0.111	0.007	-16.850	0.000	-0.124	-0.098
KS	-0.124	0.017	-7.270	0.000	-0.158	-0.091
KY	-0.092	0.014	-6.760	0.000	-0.119	-0.065
LA	-0.130	0.022	-6.010	0.000	-0.173	-0.088
MA	-0.119	0.009	-13.680	0.000	-0.136	-0.102
MD	-0.088	0.019	-4.650	0.000	-0.125	-0.051
ME	-0.123	0.014	-8.610	0.000	-0.151	-0.095
MI	-0.113	0.029	-3.960	0.000	-0.169	-0.057
MN	-0.161	0.008	-19.590	0.000	-0.177	-0.145
MO	-0.099	0.006	-16.940	0.000	-0.111	-0.088
MS	-0.077	0.033	-2.310	0.021	-0.142	-0.012
MT	-0.129	0.042	-3.040	0.002	-0.211	-0.046
NC	-0.109	0.005	-20.210	0.000	-0.120	-0.099
ND	-0.130	0.019	-6.920	0.000	-0.167	-0.093
NE	-0.110	0.011	-10.300	0.000	-0.131	-0.089
NH	-0.132	0.017	-7.740	0.000	-0.166	-0.099
NJ	-0.088	0.006	-14.040	0.000	-0.100	-0.076
NM	-0.127	0.009	-13.690	0.000	-0.145	-0.109
NV	-0.124	0.038	-3.230	0.001	-0.199	-0.049
NY	-0.123	0.006	-20.450	0.000	-0.135	-0.111
OH	-0.086	0.006	-13.870	0.000	-0.098	-0.074
OK	-0.093	0.028	-3.280	0.001	-0.148	-0.037
OR	-0.168	0.013	-12.550	0.000	-0.194	-0.142
PA	-0.123	0.010	-12.500	0.000	-0.142	-0.103
RI	-0.115	0.009	-12.910	0.000	-0.132	-0.097
SC	-0.092	0.006	-14.980	0.000	-0.104	-0.080
SD	-0.134	0.020	-6.620	0.000	-0.173	-0.094
TN	-0.079	0.006	-12.230	0.000	-0.091	-0.066
TX	-0.113	0.006	-19.280	0.000	-0.124	-0.101
UT	-0.129	0.007	-18.200	0.000	-0.143	-0.115
VA	-0.083	0.007	-12.060	0.000	-0.097	-0.070
VT	-0.120	0.019	-6.210	0.000	-0.157	-0.082
WA	-0.152	0.010	-15.400	0.000	-0.171	-0.132
WI	-0.121	0.006	-20.030	0.000	-0.133	-0.110
WV	-0.079	0.030	-2.610	0.009	-0.138	-0.020
WY	-0.217	0.046	-4.680	0.000	-0.309	-0.126
Unknown/other	-0.127	0.022	-5.650	0.000	-0.171	-0.083
race/ethnicity						

© 2019 Jeffery MM et al. *JAMA Network Open.*

Outcome: number of overlap days<br>Population: Medicare Advantage, short-term episodes	Coef.	Robust Std. Err.	z	P>z	[95 Conf.	Interval]
B	-0.011	0.002	-5.010	0.000	-0.015	-0.007
H	0.007	0.002	3.050	0.002	0.002	0.011
A	0.004	0.007	0.580	0.563	-0.009	0.017
U	-0.002	0.003	-0.650	0.519	-0.008	0.004
elix_chf	-0.005	0.003	-1.550	0.121	-0.010	0.001
elix_arrhythmia	-0.007	0.002	-2.990	0.003	-0.012	-0.002
elix_valvular	-0.003	0.004	-0.690	0.488	-0.010	0.005
elix_pulm_circ	0.002	0.005	0.340	0.736	-0.008	0.011
elix_periph_vasc	-0.008	0.003	-2.480	0.013	-0.014	-0.002
elix_htn_uncomp	-0.007	0.001	-5.170	0.000	-0.010	-0.005
elix_htn_comp	-0.009	0.004	-2.400	0.016	-0.016	-0.002
elix_oth_neuro	-0.002	0.003	-0.860	0.391	-0.008	0.003
elix_chron_pulm	0.004	0.002	2.320	0.020	0.001	0.008
elix_pep_ulcer	-0.001	0.010	-0.070	0.941	-0.020	0.019
elix_dm_wo_cc	-0.012	0.002	-6.200	0.000	-0.016	-0.008
elix_dm_w_cc	-0.011	0.003	-4.140	0.000	-0.016	-0.006
elix_paralysis	-0.013	0.007	-1.790	0.074	-0.028	0.001
elix_renal	-0.001	0.003	-0.460	0.643	-0.007	0.005
elix_solid_tumor_wo_mets	0.002	0.005	0.300	0.768	-0.009	0.012
elix_liver	-0.001	0.005	-0.130	0.900	-0.010	0.009
elix_met_cancer	0.019	0.019	0.990	0.323	-0.019	0.057
elix_hiv_aids	0.026	0.010	2.630	0.009	0.007	0.046
elix_rheum_arth_coll	-0.032	0.004	-8.760	0.000	-0.039	-0.025
elix_hypothyroid	-0.003	0.002	-1.100	0.269	-0.007	0.002
elix_lymphoma	0.011	0.016	0.670	0.501	-0.020	0.041
elix_coagulopathy	-0.004	0.006	-0.730	0.464	-0.015	0.007
elix_obesity	-0.020	0.004	-5.660	0.000	-0.027	-0.013
elix_weight_loss	-0.019	0.005	-3.800	0.000	-0.028	-0.009
elix_fluid_electrolyte	-0.008	0.003	-2.820	0.005	-0.014	-0.002
elix_blood_loss_anemia	-0.008	0.009	-0.920	0.359	-0.024	0.009
elix_deficiency_anemia	0.006	0.004	1.380	0.168	-0.002	0.014
elix_alcohol	-0.017	0.006	-2.790	0.005	-0.029	-0.005
elix_drug_abuse	0.017	0.005	3.500	0.000	0.007	0.026
elix_psychosis	0.012	0.004	3.150	0.002	0.005	0.019
elix_depression	0.019	0.002	10.820	0.000	0.015	0.022
less than 6 months prior insurance coverage	0.003	0.002	1.530	0.127	-0.001	0.007
female	0.001	0.001	0.890	0.373	-0.002	0.004
age	-0.001	0.000	-16.810	0.000	-0.001	-0.001

© 2019 Jeffery MM et al. *JAMA Network Open.*

Outcome: number of overlap days Population: Medicare Advantage, short-term episodes	Coef.	Robust Std. Err.	z	P>z	[95 Conf.	Interval]
_cons	-0.170	0.082	-2.060	0.040	-0.331	-0.008
ln(op_days)	1.000	(exposure)				
/lnalpha	-2.901	0.013			-2.926	-2.876
alpha	0.055	0.001			0.054	0.056
Observations: 454,771 person-months contributed by 193,656 individuals						

eTable 10. Regression Results: Intensity of Coprescribing for Medicare Advantage—Long-term Episodes

Outcome: number of overlap days Population: Medicare Advantage, long-term episodes	Coef.	Robust Std. Err.	z	P>z	[95 Conf.	Interval]
Spline components						
months before release	0.001	0.000	2.350	0.019	0.000	0.001
months after release	0.000	0.000	0.860	0.387	0.000	0.001
binary post-release	-0.004	0.003	-1.160	0.246	-0.010	0.003
state						
AL	-0.089	0.007	-12.340	0.000	-0.103	-0.074
AR	-0.106	0.011	-9.820	0.000	-0.127	-0.085
AZ	-0.145	0.018	-8.230	0.000	-0.180	-0.111
CA	-0.175	0.018	-9.630	0.000	-0.211	-0.140
CO	-0.189	0.023	-8.250	0.000	-0.234	-0.144
CT	-0.147	0.014	-10.550	0.000	-0.175	-0.120
DC	-0.225	0.038	-5.980	0.000	-0.298	-0.151
DE	-0.187	0.064	-2.910	0.004	-0.312	-0.061
FL	-0.097	0.004	-21.690	0.000	-0.106	-0.089
GA	-0.120	0.007	-17.780	0.000	-0.134	-0.107
HI	-0.100	0.022	-4.630	0.000	-0.142	-0.057
IA	-0.102	0.016	-6.270	0.000	-0.134	-0.070
ID	-0.167	0.022	-7.610	0.000	-0.210	-0.124
IL	-0.121	0.009	-13.560	0.000	-0.138	-0.103
IN	-0.133	0.010	-13.280	0.000	-0.152	-0.113
KS	-0.159	0.029	-5.430	0.000	-0.217	-0.102
KY	-0.114	0.027	-4.210	0.000	-0.166	-0.061
LA	-0.106	0.043	-2.500	0.012	-0.190	-0.023
MA	-0.119	0.014	-8.510	0.000	-0.146	-0.091
MD	-0.143	0.035	-4.130	0.000	-0.211	-0.075
ME	-0.172	0.034	-5.110	0.000	-0.238	-0.106
MI	-0.267	0.080	-3.350	0.001	-0.423	-0.111

© 2019 Jeffery MM et al. *JAMA Network Open.*

Outcome: number of overlap days Population: Medicare Advantage, long-term episodes	Coef.	Robust Std. Err.	z	P>z	[95 Conf.	Interval]
MN	-0.187	0.016	-11.420	0.000	-0.220	-0.155
MO	-0.119	0.008	-14.780	0.000	-0.135	-0.103
MS	-0.098	0.060	-1.630	0.103	-0.215	0.020
MT	-0.071	0.059	-1.220	0.224	-0.186	0.044
NC	-0.107	0.006	-19.320	0.000	-0.117	-0.096
ND	-0.122	0.037	-3.330	0.001	-0.194	-0.050
NE	-0.206	0.024	-8.670	0.000	-0.252	-0.159
NH	-0.185	0.042	-4.440	0.000	-0.267	-0.104
NJ	-0.116	0.010	-11.060	0.000	-0.137	-0.095
NM	-0.146	0.015	-9.980	0.000	-0.174	-0.117
NV	-0.203	0.079	-2.570	0.010	-0.358	-0.048
NY	-0.121	0.008	-16.090	0.000	-0.136	-0.106
OH	-0.105	0.008	-12.760	0.000	-0.121	-0.089
OK	-0.106	0.055	-1.910	0.056	-0.214	0.003
OR	-0.268	0.037	-7.340	0.000	-0.340	-0.197
PA	-0.151	0.017	-8.810	0.000	-0.185	-0.118
RI	-0.154	0.016	-9.540	0.000	-0.185	-0.122
SC	-0.108	0.008	-13.400	0.000	-0.123	-0.092
SD	-0.130	0.036	-3.630	0.000	-0.200	-0.060
TN	-0.081	0.007	-11.230	0.000	-0.096	-0.067
TX	-0.117	0.007	-16.220	0.000	-0.131	-0.103
UT	-0.142	0.011	-12.890	0.000	-0.163	-0.120
VA	-0.092	0.009	-10.370	0.000	-0.109	-0.075
VT	-0.228	0.058	-3.910	0.000	-0.342	-0.114
WA	-0.182	0.018	-10.140	0.000	-0.218	-0.147
WI	-0.168	0.010	-17.540	0.000	-0.187	-0.149
WV	-0.055	0.063	-0.880	0.378	-0.179	0.068
WY	-0.120	0.041	-2.940	0.003	-0.201	-0.040
Unknown/other	-0.140	0.021	-6.590	0.000	-0.181	-0.098
race/ethnicity						
B	-0.016	0.004	-3.480	0.001	-0.024	-0.007
H	-0.003	0.005	-0.650	0.518	-0.013	0.007
A	-0.070	0.023	-3.020	0.003	-0.115	-0.025
U	-0.011	0.008	-1.350	0.177	-0.026	0.005
elix_chf	-0.010	0.005	-1.870	0.062	-0.020	0.000
elix_arrhythmia	-0.014	0.005	-3.020	0.003	-0.023	-0.005
elix_valvular	-0.003	0.007	-0.460	0.647	-0.016	0.010
elix_pulm_circ	-0.010	0.010	-0.980	0.325	-0.028	0.009
elix_periph_vasc	-0.021	0.006	-3.800	0.000	-0.032	-0.010

© 2019 Jeffery MM et al. *JAMA Network Open.*

Outcome: number of overlap days<br>Population: Medicare Advantage, long-term episodes	Coef.	Robust Std. Err.	z	P>z	[95 Conf.	Interval]
elix_htn_uncomp	-0.003	0.002	-1.260	0.209	-0.008	0.002
elix_htn_comp	0.002	0.007	0.240	0.811	-0.012	0.015
elix_oth_neuro	-0.007	0.005	-1.370	0.170	-0.017	0.003
elix_chron_pulm	0.010	0.003	3.030	0.002	0.003	0.016
elix_pep_ulcer	-0.008	0.014	-0.570	0.571	-0.035	0.019
elix_dm_wo_cc	-0.010	0.004	-2.680	0.007	-0.017	-0.003
elix_dm_w_cc	-0.021	0.005	-4.690	0.000	-0.030	-0.012
elix_paralysis	-0.018	0.013	-1.440	0.151	-0.043	0.007
elix_renal	-0.019	0.006	-3.210	0.001	-0.031	-0.008
elix_solid_tumor_wo_mets	-0.007	0.017	-0.390	0.700	-0.041	0.027
elix_liver	-0.001	0.007	-0.140	0.891	-0.015	0.013
elix_met_cancer	0.059	0.045	1.320	0.187	-0.029	0.148
elix_hiv_aids	0.015	0.021	0.720	0.470	-0.026	0.056
elix_rheum_arth_coll	-0.034	0.006	-5.890	0.000	-0.046	-0.023
elix_hypothyroid	-0.008	0.005	-1.630	0.103	-0.017	0.002
elix_lymphoma	0.040	0.039	1.010	0.314	-0.038	0.117
elix_coagulopathy	-0.009	0.010	-0.830	0.406	-0.029	0.012
elix_obesity	-0.016	0.006	-2.740	0.006	-0.027	-0.004
elix_weight_loss	-0.019	0.008	-2.300	0.022	-0.034	-0.003
elix_fluid_electrolyte	-0.008	0.005	-1.680	0.093	-0.017	0.001
elix_blood_loss_anemia	-0.002	0.013	-0.120	0.901	-0.027	0.024
elix_deficiency_anemia	-0.004	0.007	-0.500	0.618	-0.017	0.010
elix_alcohol	-0.022	0.009	-2.280	0.022	-0.040	-0.003
elix_drug_abuse	-0.002	0.006	-0.380	0.703	-0.014	0.009
elix_psychosis	0.004	0.006	0.680	0.498	-0.008	0.017
elix_depression	0.012	0.003	3.950	0.000	0.006	0.018
less than 6 months prior insurance coverage	-0.004	0.005	-0.880	0.380	-0.014	0.006
female	-0.012	0.003	-3.790	0.000	-0.017	-0.006
age	-0.001	0.000	-11.090	0.000	-0.002	-0.001
_cons	-0.318	0.156	-2.030	0.042	-0.623	-0.012
ln(op_days)	1.000	(exposure)				
/lnalpha	-2.410	0.018			-2.445	-2.374
alpha	0.090	0.002			0.087	0.093

Observations: 228,195 person-months contributed by 24,979 individuals

© 2019 Jeffery MM et al. *JAMA Network Open.*

eTable 11. Regression Results: Intensity of Coprescription Among Commercial Beneficiaries With and Without Same Physician Prescribing Opioids and Benzodiazepines—Short-term Opioid Use Episodes

Outcome: number of overlap days<br>Population: Commercial short-term episodes	IRR	Robust Std. Err.	z	P>z	[95 Conf.	Interval]
Same physician prescribing both medications						
Yes	0.577	0.091	-3.500	0.000	0.424	0.785
Unknown	0.841	0.290	-0.500	0.616	0.428	1.653
Spline components						
months before release	1.000	0.000	-0.130	0.895	1.000	1.000
months after release	1.000	0.000	0.030	0.977	0.999	1.001
binary post-release	0.996	0.004	-1.030	0.303	0.989	1.004
same physician#months before release						
Yes	1.001	0.000	3.230	0.001	1.000	1.001
Unknown	1.000	0.001	0.210	0.837	0.999	1.001
same physician#months after release						
Yes	1.000	0.000	-1.200	0.230	0.999	1.000
Unknown	1.001	0.001	1.770	0.077	1.000	1.003
same physician#binary post-release						
Yes	0.996	0.005	-0.760	0.445	0.986	1.006
Unknown	0.983	0.011	-1.510	0.132	0.961	1.005
state						
AL	1.086	0.032	2.830	0.005	1.026	1.150
AR	1.058	0.031	1.930	0.053	0.999	1.120
AZ	0.999	0.029	-0.020	0.983	0.945	1.057
CA	1.009	0.029	0.330	0.744	0.954	1.068
CO	0.971	0.028	-1.040	0.300	0.917	1.027
CT	1.010	0.030	0.330	0.744	0.952	1.071
DC	0.976	0.033	-0.710	0.478	0.914	1.043
DE	1.001	0.036	0.020	0.984	0.932	1.075
FL	1.032	0.030	1.100	0.272	0.976	1.091
GA	0.999	0.029	-0.050	0.961	0.944	1.057
HI	1.139	0.063	2.370	0.018	1.023	1.270
IA	0.999	0.029	-0.040	0.969	0.943	1.058
ID	0.944	0.033	-1.660	0.097	0.883	1.010
IL	1.021	0.029	0.710	0.481	0.965	1.080
IN	1.028	0.030	0.960	0.335	0.972	1.088
KS	1.035	0.031	1.180	0.239	0.977	1.097
KY	1.023	0.031	0.770	0.439	0.965	1.085
LA	1.026	0.030	0.900	0.367	0.970	1.086
MA	1.001	0.030	0.050	0.964	0.944	1.062

© 2019 Jeffery MM et al. *JAMA Network Open.*

Outcome: number of overlap days<br>Population: Commercial short-term episodes	IRR	Robust Std. Err.	z	P>z	[95 Conf.	Interval]
MD	1.017	0.029	0.580	0.560	0.961	1.077
ME	0.994	0.041	-0.140	0.892	0.917	1.078
MI	1.056	0.031	1.870	0.062	0.997	1.118
MN	0.946	0.028	-1.910	0.056	0.893	1.002
MO	1.038	0.030	1.290	0.198	0.981	1.098
MS	1.049	0.031	1.620	0.104	0.990	1.110
MT	1.006	0.040	0.150	0.884	0.931	1.087
NC	1.009	0.029	0.330	0.745	0.954	1.068
ND	0.960	0.038	-1.030	0.302	0.889	1.037
NE	0.984	0.029	-0.550	0.579	0.929	1.042
NH	0.978	0.032	-0.680	0.494	0.916	1.043
NJ	1.035	0.030	1.180	0.239	0.977	1.096
NM	1.004	0.031	0.140	0.886	0.945	1.068
NV	1.051	0.031	1.670	0.095	0.991	1.114
NY	1.040	0.030	1.350	0.178	0.982	1.101
OH	1.005	0.029	0.170	0.864	0.950	1.063
OK	1.013	0.030	0.440	0.658	0.957	1.073
OR	0.881	0.027	-4.090	0.000	0.829	0.936
PA	1.033	0.030	1.110	0.265	0.976	1.094
RI	1.023	0.031	0.750	0.451	0.964	1.086
SC	1.055	0.031	1.840	0.065	0.997	1.118
SD	0.971	0.041	-0.690	0.493	0.894	1.055
TN	1.039	0.030	1.320	0.186	0.982	1.100
TX	1.005	0.029	0.180	0.856	0.950	1.063
UT	0.989	0.029	-0.390	0.697	0.933	1.048
VA	1.004	0.029	0.130	0.896	0.948	1.063
VT	0.944	0.059	-0.920	0.360	0.835	1.068
WA	0.914	0.027	-2.990	0.003	0.862	0.970
WI	0.975	0.028	-0.860	0.389	0.922	1.032
WV	1.054	0.034	1.630	0.103	0.990	1.122
WY	0.956	0.037	-1.160	0.247	0.886	1.032
Unknown/other	1.026	0.032	0.800	0.423	0.964	1.091
race/ethnicity						
B	0.988	0.003	-4.270	0.000	0.982	0.993
H	0.996	0.003	-1.390	0.165	0.990	1.002
A	0.957	0.006	-6.880	0.000	0.945	0.969
U	0.997	0.004	-0.700	0.486	0.989	1.005
elix_chf	1.001	0.007	0.100	0.920	0.987	1.015
elix_arrhythmia	0.993	0.005	-1.600	0.110	0.984	1.002

© 2019 Jeffery MM et al. *JAMA Network Open.*

Outcome: number of overlap days<br>Population: Commercial short-term episodes	IRR	Robust Std. Err.	z	P>z	[95 Conf.	Interval]
elix_valvular	0.990	0.007	-1.300	0.193	0.976	1.005
elix_pulm_circ	0.997	0.010	-0.290	0.770	0.978	1.017
elix_periph_vasc	0.993	0.008	-0.890	0.376	0.979	1.008
elix_htn_uncomp	1.005	0.002	2.370	0.018	1.001	1.009
elix_htn_comp	1.003	0.008	0.350	0.730	0.986	1.020
elix_oth_neuro	1.037	0.005	7.330	0.000	1.027	1.048
elix_chron_pulm	1.017	0.003	5.130	0.000	1.010	1.023
elix_pep_ulcer	1.012	0.016	0.730	0.464	0.981	1.043
elix_dm_wo_cc	0.995	0.003	-1.420	0.157	0.988	1.002
elix_dm_w_cc	1.003	0.005	0.670	0.505	0.993	1.014
elix_paralysis	1.014	0.012	1.130	0.260	0.990	1.038
elix_renal	1.003	0.008	0.360	0.721	0.988	1.018
elix_solid_tumor_wo_mets	1.004	0.007	0.510	0.607	0.989	1.019
elix_liver	1.009	0.007	1.400	0.162	0.996	1.023
elix_met_cancer	0.949	0.030	-1.660	0.097	0.891	1.010
elix_hiv_aids	1.060	0.015	4.250	0.000	1.032	1.090
elix_rheum_arth_coll	0.992	0.005	-1.800	0.071	0.983	1.001
elix_hypothyroid	1.004	0.003	1.040	0.298	0.997	1.010
elix_lymphoma	1.019	0.022	0.880	0.380	0.977	1.062
elix_coagulopathy	0.994	0.009	-0.640	0.523	0.977	1.012
elix_obesity	0.977	0.004	-5.470	0.000	0.969	0.985
elix_weight_loss	0.992	0.010	-0.850	0.397	0.973	1.011
elix_fluid_electrolyte	0.986	0.005	-2.730	0.006	0.977	0.996
elix_blood_loss_anemia	0.978	0.015	-1.380	0.166	0.949	1.009
elix_deficiency_anemia	1.022	0.008	2.980	0.003	1.008	1.037
elix_alcohol	0.995	0.007	-0.770	0.442	0.981	1.008
elix_drug_abuse	1.056	0.007	8.540	0.000	1.043	1.070
elix_psychosis	1.054	0.008	6.950	0.000	1.038	1.070
elix_depression	1.068	0.002	32.360	0.000	1.064	1.072
less than 6 months prior insurance coverage	1.011	0.002	5.320	0.000	1.007	1.016
female	1.019	0.002	11.390	0.000	1.016	1.022
age	1.003	0.000	47.370	0.000	1.003	1.003
_cons	0.665	0.077	-3.510	0.000	0.530	0.835
ln(op_days)	1.000	(exposure)				
/lnalpha	-2.499	0.010			-2.518	-2.480
alpha	0.082	0.001			0.081	0.084

Observations: 583,020 person-months contributed by 344,179 individuals

© 2019 Jeffery MM et al. *JAMA Network Open.*

eTable 12. Regression Results: Intensity of Coprescription Among Commercial Beneficiaries With and Without Same Physician Prescribing Opioids and Benzodiazepines: Long-term Opioid Use Episodes

Outcome: number of overlap days Population: Commercial, long-term episodes	IRR	Robust Std. Err.	z	P>z	[95 Conf.	Interval]
Same physician prescribing both medications						
Yes	0.912	0.346	-0.240	0.808	0.434	1.918
Unknown	0.237	0.320	-1.060	0.287	0.017	3.357
Spline components						
months before release	1.001	0.000	1.200	0.229	1.000	1.001
months after release	0.999	0.001	-1.310	0.190	0.998	1.000
binary post-release	1.007	0.007	1.060	0.287	0.994	1.021
same physician#months before release						
Yes	1.000	0.001	0.370	0.709	0.999	1.001
Unknown	1.002	0.002	1.030	0.303	0.998	1.006
same physician#months after release						
Yes	1.000	0.000	-1.200	0.230	0.999	1.000
Unknown	1.001	0.001	1.770	0.077	1.000	1.003
same physician#binary post-release						
Yes	1.001	0.001	0.990	0.323	0.999	1.002
Unknown	0.999	0.003	-0.320	0.747	0.994	1.005
state						
AL	1.387	0.171	2.650	0.008	1.088	1.766
AR	1.340	0.166	2.370	0.018	1.052	1.707
AZ	1.265	0.156	1.910	0.057	0.993	1.610
CA	1.295	0.160	2.100	0.036	1.017	1.648
CO	1.246	0.154	1.780	0.075	0.978	1.587
CT	1.260	0.159	1.830	0.067	0.984	1.614
DC	1.273	0.176	1.740	0.081	0.970	1.670
DE	1.303	0.174	1.980	0.048	1.003	1.692
FL	1.351	0.166	2.450	0.014	1.062	1.719
GA	1.316	0.162	2.230	0.026	1.033	1.676
HI	1.364	0.218	1.950	0.051	0.998	1.865
IA	1.306	0.163	2.140	0.033	1.022	1.668
ID	1.310	0.167	2.130	0.034	1.021	1.681
IL	1.344	0.165	2.400	0.016	1.056	1.711
IN	1.339	0.165	2.370	0.018	1.051	1.704
KS	1.298	0.162	2.090	0.036	1.017	1.656
KY	1.391	0.172	2.670	0.008	1.092	1.773
LA	1.356	0.167	2.470	0.014	1.065	1.726
MA	1.302	0.165	2.080	0.038	1.015	1.669

© 2019 Jeffery MM et al. *JAMA Network Open.*

Outcome: number of overlap days<br>Population: Commercial, long-term episodes	IRR	Robust Std. Err.	z	P>z	[95 Conf.	Interval]
MD	1.326	0.164	2.280	0.023	1.041	1.691
ME	1.196	0.197	1.090	0.277	0.866	1.651
MI	1.335	0.165	2.340	0.019	1.048	1.700
MN	1.230	0.153	1.670	0.095	0.965	1.569
MO	1.314	0.162	2.210	0.027	1.032	1.672
MS	1.365	0.169	2.520	0.012	1.072	1.739
MT	1.306	0.183	1.910	0.056	0.993	1.719
NC	1.333	0.164	2.330	0.020	1.047	1.697
ND	1.319	0.170	2.150	0.031	1.025	1.697
NE	1.302	0.162	2.120	0.034	1.020	1.662
NH	1.216	0.173	1.370	0.171	0.919	1.608
NJ	1.328	0.165	2.280	0.022	1.041	1.694
NM	1.302	0.165	2.090	0.037	1.017	1.668
NV	1.342	0.168	2.360	0.018	1.051	1.715
NY	1.309	0.162	2.170	0.030	1.027	1.669
OH	1.319	0.163	2.250	0.024	1.036	1.680
OK	1.338	0.165	2.360	0.018	1.051	1.705
OR	0.995	0.134	-0.040	0.971	0.765	1.295
PA	1.348	0.167	2.410	0.016	1.058	1.718
RI	1.303	0.165	2.090	0.036	1.017	1.669
SC	1.315	0.163	2.210	0.027	1.031	1.677
SD	1.184	0.200	1.000	0.316	0.851	1.648
TN	1.348	0.166	2.420	0.015	1.059	1.717
TX	1.303	0.160	2.150	0.031	1.024	1.658
UT	1.272	0.159	1.930	0.054	0.996	1.624
VA	1.258	0.156	1.850	0.064	0.987	1.604
VT	1.465	0.195	2.870	0.004	1.128	1.902
WA	1.168	0.146	1.240	0.214	0.914	1.493
WI	1.290	0.159	2.060	0.039	1.012	1.643
WV	1.340	0.175	2.250	0.025	1.038	1.730
WY	1.306	0.188	1.860	0.063	0.985	1.732
Unknown/other	1.323	0.166	2.230	0.026	1.035	1.692
race/ethnicity						
B	0.981	0.007	-2.700	0.007	0.968	0.995
H	0.990	0.008	-1.320	0.188	0.975	1.005
A	0.975	0.024	-1.030	0.304	0.930	1.023
U	0.995	0.011	-0.470	0.638	0.973	1.017
elix_chf	0.977	0.014	-1.680	0.093	0.950	1.004
elix_arrhythmia	1.008	0.008	0.940	0.347	0.992	1.024

© 2019 Jeffery MM et al. *JAMA Network Open.*

Outcome: number of overlap days<br>Population: Commercial, long-term episodes	IRR	Robust Std. Err.	z	P>z	[95 Conf.	Interval]
elix_valvular	0.991	0.014	-0.620	0.535	0.963	1.020
elix_pulm_circ	0.982	0.021	-0.870	0.386	0.942	1.023
elix_periph_vasc	0.957	0.014	-3.000	0.003	0.930	0.985
elix_htn_uncomp	0.995	0.004	-1.370	0.170	0.987	1.002
elix_htn_comp	1.001	0.015	0.070	0.947	0.972	1.031
elix_oth_neuro	1.021	0.009	2.380	0.017	1.004	1.039
elix_chron_pulm	1.009	0.006	1.410	0.157	0.997	1.021
elix_pep_ulcer	0.990	0.023	-0.430	0.664	0.947	1.036
elix_dm_wo_cc	0.992	0.007	-1.220	0.221	0.979	1.005
elix_dm_w_cc	0.988	0.010	-1.230	0.218	0.969	1.007
elix_paralysis	0.962	0.021	-1.780	0.075	0.923	1.004
elix_renal	1.017	0.015	1.100	0.273	0.987	1.047
elix_solid_tumor_wo_mets	0.958	0.031	-1.310	0.189	0.899	1.021
elix_liver	0.999	0.011	-0.100	0.924	0.977	1.021
elix_met_cancer	0.654	0.173	-1.600	0.109	0.389	1.099
elix_hiv_aids	1.063	0.029	2.260	0.024	1.008	1.122
elix_rheum_arth_coll	0.983	0.008	-2.160	0.031	0.968	0.998
elix_hypothyroid	0.989	0.008	-1.470	0.140	0.974	1.004
elix_lymphoma	0.925	0.102	-0.700	0.482	0.745	1.149
elix_coagulopathy	1.009	0.015	0.570	0.569	0.980	1.038
elix_obesity	0.983	0.007	-2.250	0.024	0.969	0.998
elix_weight_loss	0.981	0.015	-1.270	0.206	0.953	1.010
elix_fluid_electrolyte	0.989	0.008	-1.350	0.178	0.974	1.005
elix_blood_loss_anemia	0.999	0.027	-0.030	0.975	0.948	1.053
elix_deficiency_anemia	1.031	0.014	2.290	0.022	1.004	1.058
elix_alcohol	0.991	0.014	-0.630	0.528	0.965	1.019
elix_drug_abuse	0.992	0.009	-0.970	0.330	0.975	1.009
elix_psychosis	1.060	0.019	3.190	0.001	1.023	1.099
elix_depression	1.052	0.004	11.990	0.000	1.043	1.061
less than 6 months prior insurance coverage	0.998	0.006	-0.330	0.742	0.987	1.010
female	0.993	0.004	-1.650	0.099	0.985	1.001
age	1.001	0.000	6.410	0.000	1.001	1.002
_cons	0.379	0.127	-2.890	0.004	0.197	0.732
ln(op_days)	1.000	(exposure)				
/lnalpha	-1.919	0.015			-1.948	-1.889
alpha	0.147	0.002			0.142	0.151
Observations: 168,810 person-months contributed by 22,290 individuals						

© 2019 Jeffery MM et al. *JAMA Network Open.*

eTable 13. Regression Results: Intensity of Coprescription Among Medicare Advantage Beneficiaries With and Without Same Physician Prescribing Opioids and Benzodiazepines—Short-term Episodes

Outcome: number of overlap days Population: Medicare Advantage short-term episodes	IRR	Robust Std. Err.	z	P>z	[95 Conf.	Interval]
Same physician prescribing both medications						
Yes	0.848	0.147	-0.950	0.342	0.603	1.192
Unknown	0.991	0.342	-0.030	0.979	0.503	1.950
Spline components						
months before release	1.000	0.000	0.870	0.383	1.000	1.001
months after release	1.000	0.000	-0.160	0.870	0.999	1.001
binary post-release	0.999	0.004	-0.290	0.769	0.991	1.007
same physician#months before release						
Yes	1.000	0.000	1.050	0.292	1.000	1.001
Unknown	1.000	0.001	0.000	0.996	0.999	1.001
same physician#months after release						
Yes	1.000	0.000	0.890	0.375	1.000	1.001
Unknown	1.000	0.001	0.020	0.980	0.999	1.001
same physician#binary post-release						
Yes	0.996	0.005	-0.820	0.413	0.986	1.006
Unknown	1.003	0.009	0.290	0.773	0.984	1.021
state						
AL	0.910	0.005	-17.470	0.000	0.900	0.920
AR	0.914	0.006	-13.600	0.000	0.902	0.926
AZ	0.890	0.007	-14.980	0.000	0.877	0.904
CA	0.858	0.007	-18.380	0.000	0.844	0.872
CO	0.867	0.010	-12.410	0.000	0.847	0.887
CT	0.881	0.006	-18.070	0.000	0.869	0.894
DC	0.883	0.018	-6.040	0.000	0.848	0.919
DE	0.819	0.040	-4.050	0.000	0.743	0.902
FL	0.907	0.004	-22.860	0.000	0.900	0.915
GA	0.881	0.004	-26.430	0.000	0.873	0.889
HI	0.890	0.011	-9.760	0.000	0.869	0.911
IA	0.875	0.008	-14.540	0.000	0.860	0.891
ID	0.852	0.011	-12.840	0.000	0.832	0.873
IL	0.885	0.005	-23.500	0.000	0.876	0.894
IN	0.885	0.005	-20.550	0.000	0.875	0.895
KS	0.872	0.015	-7.950	0.000	0.843	0.902
KY	0.898	0.012	-7.760	0.000	0.874	0.923
LA	0.868	0.019	-6.520	0.000	0.832	0.906

© 2019 Jeffery MM et al. *JAMA Network Open.*

Outcome: number of overlap days<br>Population: Medicare Advantage short-term episodes	IRR	Robust Std. Err.	z	P>z	[95 Conf.	Interval]
MA	0.874	0.007	-15.910	0.000	0.860	0.889
MD	0.905	0.018	-5.180	0.000	0.871	0.940
ME	0.868	0.013	-9.720	0.000	0.844	0.893
MI	0.881	0.026	-4.350	0.000	0.832	0.933
MN	0.833	0.007	-23.070	0.000	0.820	0.846
MO	0.894	0.005	-22.020	0.000	0.885	0.903
MS	0.913	0.031	-2.710	0.007	0.855	0.975
MT	0.867	0.038	-3.250	0.001	0.796	0.945
NC	0.887	0.004	-26.490	0.000	0.879	0.895
ND	0.862	0.017	-7.670	0.000	0.830	0.896
NE	0.884	0.009	-11.760	0.000	0.866	0.903
NH	0.860	0.015	-8.490	0.000	0.831	0.891
NJ	0.904	0.005	-18.160	0.000	0.894	0.914
NM	0.870	0.008	-15.550	0.000	0.855	0.885
NV	0.874	0.034	-3.420	0.001	0.810	0.944
NY	0.874	0.005	-25.530	0.000	0.865	0.883
OH	0.905	0.005	-18.110	0.000	0.895	0.915
OK	0.902	0.026	-3.570	0.000	0.853	0.955
OR	0.827	0.011	-13.830	0.000	0.805	0.850
PA	0.875	0.008	-14.050	0.000	0.858	0.891
RI	0.879	0.008	-14.950	0.000	0.864	0.894
SC	0.901	0.005	-19.050	0.000	0.892	0.911
SD	0.862	0.018	-7.230	0.000	0.828	0.898
TN	0.914	0.005	-15.610	0.000	0.904	0.924
TX	0.884	0.004	-24.250	0.000	0.876	0.893
UT	0.865	0.006	-22.040	0.000	0.854	0.877
VA	0.910	0.006	-14.970	0.000	0.899	0.921
VT	0.871	0.017	-6.950	0.000	0.838	0.906
WA	0.843	0.008	-17.430	0.000	0.827	0.859
WI	0.871	0.005	-25.800	0.000	0.862	0.880
WV	0.917	0.028	-2.840	0.004	0.863	0.973
WY	0.787	0.038	-4.960	0.000	0.715	0.865
Unknown/other	0.869	0.020	-6.140	0.000	0.831	0.909
race/ethnicity						
B	0.989	0.002	-4.850	0.000	0.985	0.994
H	1.010	0.002	4.160	0.000	1.005	1.014
A	1.003	0.007	0.510	0.612	0.990	1.017
U	0.997	0.003	-0.870	0.386	0.991	1.004
elix_chf	0.996	0.003	-1.430	0.153	0.990	1.002

© 2019 Jeffery MM et al. *JAMA Network Open.*

Outcome: number of overlap days<br>Population: Medicare Advantage short-term episodes	IRR	Robust Std. Err.	z	P>z	[95 Conf.	Interval]
elix_arrhythmia	0.992	0.002	-3.190	0.001	0.987	0.997
elix_valvular	0.997	0.004	-0.780	0.433	0.989	1.005
elix_pulm_circ	1.003	0.005	0.560	0.573	0.993	1.013
elix_periph_vasc	0.992	0.003	-2.440	0.015	0.986	0.998
elix_htn_uncomp	0.994	0.001	-4.420	0.000	0.991	0.996
elix_htn_comp	0.991	0.004	-2.310	0.021	0.984	0.999
elix_oth_neuro	0.998	0.003	-0.640	0.523	0.993	1.004
elix_chron_pulm	1.006	0.002	2.960	0.003	1.002	1.009
elix_pep_ulcer	0.999	0.010	-0.080	0.936	0.980	1.019
elix_dm_wo_cc	0.988	0.002	-5.950	0.000	0.984	0.992
elix_dm_w_cc	0.990	0.003	-3.790	0.000	0.985	0.995
elix_paralysis	0.987	0.008	-1.690	0.091	0.973	1.002
elix_renal	0.999	0.003	-0.360	0.718	0.993	1.005
elix_solid_tumor_wo_mets	0.998	0.005	-0.380	0.701	0.988	1.008
elix_liver	0.999	0.005	-0.160	0.874	0.990	1.009
elix_met_cancer	1.023	0.020	1.170	0.242	0.985	1.063
elix_hiv_aids	1.029	0.011	2.730	0.006	1.008	1.050
elix_rheum_arth_coll	0.975	0.004	-6.800	0.000	0.968	0.982
elix_hypothyroid	0.998	0.002	-0.900	0.366	0.993	1.003
elix_lymphoma	1.008	0.016	0.490	0.626	0.976	1.041
elix_coagulopathy	0.996	0.006	-0.620	0.536	0.985	1.008
elix_obesity	0.981	0.004	-5.310	0.000	0.974	0.988
elix_weight_loss	0.982	0.005	-3.670	0.000	0.972	0.991
elix_fluid_electrolyte	0.991	0.003	-2.900	0.004	0.986	0.997
elix_blood_loss_anemia	0.993	0.009	-0.770	0.442	0.977	1.010
elix_deficiency_anemia	1.007	0.004	1.550	0.121	0.998	1.015
elix_alcohol	0.981	0.006	-3.070	0.002	0.969	0.993
elix_drug_abuse	1.019	0.005	3.870	0.000	1.009	1.029
elix_psychosis	1.012	0.004	3.130	0.002	1.005	1.020
elix_depression	1.024	0.002	12.840	0.000	1.020	1.027
less than 6 months prior insurance coverage	1.001	0.002	0.580	0.564	0.997	1.005
female	1.004	0.002	2.500	0.012	1.001	1.007
age	0.999	0.000	-16.970	0.000	0.999	0.999
_cons	0.887	0.121	-0.880	0.380	0.679	1.159
ln(op_days)	1.000	(exposure)				
/lnalpha	-2.826	0.013			-2.851	-2.801
alpha	0.059	0.001			0.058	0.061
Observations: 454,771 person-months contributed by 193,656 individuals						

© 2019 Jeffery MM et al. *JAMA Network Open.*

eTable 14. Regression Results: Intensity of Coprescription Among Medicare Advantage Beneficiaries With and Without Same Physician Prescribing Opioids and Benzodiazepines—Long-term Episodes

Outcome: number of overlap days Population: Medicare Advantage long-term episodes	IRR	Robust Std. Err.	z	P>z	[95 Conf.	Interval]
Same physician prescribing both medications						
Yes	0.816	0.268	-0.620	0.536	0.428	1.555
Unknown	1.116	0.781	0.160	0.876	0.283	4.400
Spline components						
months before release	1.000	0.000	1.170	0.242	1.000	1.001
months after release	1.000	0.001	0.470	0.641	0.999	1.001
binary post-release	0.989	0.006	-1.750	0.081	0.977	1.001
same physician#months before release						
Yes	1.000	0.000	0.730	0.466	0.999	1.001
Unknown	1.000	0.001	-0.200	0.845	0.998	1.002
same physician#months after release						
Yes	1.000	0.001	-0.270	0.787	0.999	1.001
Unknown	1.001	0.001	0.650	0.516	0.998	1.003
same physician#binary post-release						
Yes	1.008	0.007	1.090	0.274	0.994	1.023
Unknown	1.021	0.014	1.530	0.125	0.994	1.048
state						
AL	0.926	0.007	-10.540	0.000	0.913	0.939
AR	0.911	0.010	-8.580	0.000	0.892	0.931
AZ	0.876	0.015	-7.600	0.000	0.847	0.907
CA	0.850	0.016	-8.830	0.000	0.820	0.881
CO	0.844	0.019	-7.450	0.000	0.807	0.882
CT	0.875	0.012	-9.480	0.000	0.851	0.900
DC	0.809	0.030	-5.660	0.000	0.751	0.870
DE	0.858	0.056	-2.370	0.018	0.755	0.974
FL	0.919	0.004	-17.710	0.000	0.910	0.928
GA	0.897	0.006	-15.750	0.000	0.885	0.909
HI	0.922	0.021	-3.560	0.000	0.882	0.964
IA	0.917	0.015	-5.220	0.000	0.887	0.947
ID	0.859	0.019	-6.820	0.000	0.822	0.897
IL	0.898	0.008	-11.840	0.000	0.882	0.914
IN	0.888	0.009	-11.750	0.000	0.871	0.906
KS	0.856	0.025	-5.360	0.000	0.809	0.906
KY	0.910	0.025	-3.410	0.001	0.862	0.961

© 2019 Jeffery MM et al. *JAMA Network Open.*

Outcome: number of overlap days Population: Medicare Advantage long-term episodes	IRR	Robust Std. Err.	z	P>z	[95 Conf.	Interval]
LA	0.921	0.042	-1.800	0.072	0.842	1.008
MA	0.903	0.013	-7.140	0.000	0.878	0.929
MD	0.885	0.030	-3.560	0.000	0.828	0.947
ME	0.851	0.028	-4.900	0.000	0.797	0.908
MI	0.776	0.061	-3.220	0.001	0.665	0.906
MN	0.839	0.014	-10.520	0.000	0.812	0.867
MO	0.896	0.007	-13.280	0.000	0.881	0.911
MS	0.922	0.054	-1.390	0.166	0.822	1.034
MT	0.930	0.054	-1.250	0.212	0.830	1.042
NC	0.912	0.005	-15.930	0.000	0.901	0.922
ND	0.902	0.035	-2.700	0.007	0.837	0.972
NE	0.826	0.020	-7.990	0.000	0.788	0.866
NH	0.841	0.034	-4.280	0.000	0.776	0.910
NJ	0.901	0.010	-9.800	0.000	0.882	0.920
NM	0.878	0.013	-8.780	0.000	0.853	0.904
NV	0.820	0.064	-2.550	0.011	0.704	0.955
NY	0.895	0.007	-14.570	0.000	0.881	0.908
OH	0.912	0.008	-10.810	0.000	0.897	0.928
OK	0.913	0.051	-1.610	0.107	0.818	1.020
OR	0.776	0.029	-6.890	0.000	0.722	0.834
PA	0.875	0.015	-7.810	0.000	0.846	0.905
RI	0.871	0.014	-8.560	0.000	0.843	0.899
SC	0.911	0.007	-11.450	0.000	0.896	0.925
SD	0.889	0.032	-3.280	0.001	0.829	0.954
TN	0.934	0.007	-9.390	0.000	0.921	0.948
TX	0.900	0.007	-14.340	0.000	0.887	0.913
UT	0.877	0.010	-11.850	0.000	0.858	0.896
VA	0.921	0.008	-9.240	0.000	0.905	0.937
VT	0.806	0.048	-3.630	0.000	0.717	0.905
WA	0.845	0.015	-9.210	0.000	0.816	0.876
WI	0.859	0.008	-15.380	0.000	0.842	0.876
WV	0.945	0.058	-0.920	0.357	0.838	1.066
WY	0.886	0.036	-2.980	0.003	0.818	0.959
Unknown/other	0.881	0.019	-5.790	0.000	0.844	0.920
race/ethnicity						
B	0.985	0.004	-3.480	0.001	0.976	0.993
H	0.998	0.005	-0.360	0.721	0.988	1.008
A	0.931	0.022	-3.050	0.002	0.890	0.975
U	0.991	0.008	-1.180	0.239	0.975	1.006

© 2019 Jeffery MM et al. *JAMA Network Open.*

Outcome: number of overlap days<br>Population: Medicare Advantage long-term episodes	IRR	Robust Std. Err.	z	P>z	[95 Conf.	Interval]
elix_chf	0.990	0.005	-1.970	0.049	0.980	1.000
elix_arrhythmia	0.987	0.004	-2.970	0.003	0.978	0.995
elix_valvular	0.996	0.007	-0.530	0.597	0.983	1.010
elix_pulm_circ	0.989	0.009	-1.180	0.239	0.970	1.008
elix_periph_vasc	0.978	0.005	-3.980	0.000	0.967	0.989
elix_htn_uncomp	0.996	0.002	-1.500	0.135	0.992	1.001
elix_htn_comp	1.001	0.007	0.110	0.909	0.988	1.014
elix_oth_neuro	0.993	0.005	-1.420	0.155	0.983	1.003
elix_chron_pulm	1.010	0.003	3.110	0.002	1.004	1.016
elix_pep_ulcer	0.990	0.014	-0.730	0.466	0.963	1.017
elix_dm_wo_cc	0.991	0.004	-2.590	0.010	0.984	0.998
elix_dm_w_cc	0.979	0.004	-4.680	0.000	0.970	0.988
elix_paralysis	0.981	0.012	-1.510	0.131	0.957	1.006
elix_renal	0.982	0.006	-3.040	0.002	0.970	0.994
elix_solid_tumor_wo_mets	0.990	0.017	-0.560	0.578	0.957	1.025
elix_liver	0.998	0.007	-0.280	0.779	0.984	1.012
elix_met_cancer	1.065	0.046	1.450	0.146	0.978	1.160
elix_hiv_aids	1.020	0.022	0.900	0.366	0.977	1.064
elix_rheum_arth_coll	0.974	0.006	-4.470	0.000	0.963	0.985
elix_hypothyroid	0.993	0.005	-1.490	0.136	0.984	1.002
elix_lymphoma	1.035	0.041	0.870	0.384	0.958	1.117
elix_coagulopathy	0.991	0.010	-0.890	0.372	0.971	1.011
elix_obesity	0.985	0.006	-2.650	0.008	0.974	0.996
elix_weight_loss	0.980	0.008	-2.560	0.010	0.964	0.995
elix_fluid_electrolyte	0.991	0.005	-1.940	0.052	0.982	1.000
elix_blood_loss_anemia	1.000	0.013	0.020	0.985	0.975	1.026
elix_deficiency_anemia	0.996	0.007	-0.570	0.570	0.982	1.010
elix_alcohol	0.978	0.009	-2.330	0.020	0.960	0.996
elix_drug_abuse	1.002	0.006	0.410	0.685	0.991	1.014
elix_psychosis	1.009	0.007	1.450	0.148	0.997	1.022
elix_depression	1.020	0.003	6.470	0.000	1.014	1.026
less than 6 months prior insurance coverage	0.994	0.005	-1.150	0.250	0.984	1.004
female	0.989	0.003	-3.540	0.000	0.983	0.995
age	0.998	0.000	-13.150	0.000	0.998	0.999
_cons	0.742	0.210	-1.060	0.291	0.426	1.292
ln(op_days)	1.000	(exposure)				
/lnalpha	-2.412	0.018			-2.447	-2.376
alpha	0.090	0.002			0.087	0.093
Observations: 228,195 person-months contributed by 24,979 individuals						

© 2019 Jeffery MM et al. *JAMA Network Open.*

eTable 15. Intensity of Coprescribing Before and After the Guideline Release: Comparing Same Physician Prescribing to Different Physicians Prescribing Opioids and Benzodiazepines

	Population	Commercially insured				Medicare Advantage	
Coprescribing type	Opioid episode type	Long-term	Short-term			Long-term	Short-term
		Value (95% CI)	Value (95% CI)			Value (95% CI)	Value (95% CI)
		Coprescribing intensity: Percent of opioid days in month with benzodiazepines available					
Same physician prescribing opioids and benzodiazepines	Before the guideline	80.51 (80.04 to 80.99)	75.09 (74.87 to 75.30)			84.54 (84.12 to 84.95)	83.42 (83.20 to 83.64)
	After the guideline	81.39 (80.91 to 81.87)	75.68 (75.44 to 75.91)			86.00 (85.67 to 86.33)	84.31 (84.11 to 84.50)
Different physicians prescribing opioids and benzodiazepines	Before the guideline	76.65 (76.02 to 77.27)	78.34 (78.13 to 78.55)			81.53 (80.96 to 82.10)	81.93 (81.67 to 82.19)
	After the guideline	77.45 (76.86 to 78.04)	78.24 (78.00 to 78.47)			81.79 (81.33 to 82.26)	82.24 (82.01 to 82.47)
Unknown	Before the guideline	72.94 (70.79 to 75.08)	70.74 (70.20 to 71.28)			79.36 (78.17 to 80.54)	81.04 (80.44 to 81.64)
	After the guideline	73.73 (71.66 to 75.81)	70.79 (70.22 to 0.00)			81.77 (80.99 to 82.55)	81.58 (81.15 to 82.01)
		Absolute difference in coprescribing intensity (base level = Different physicians; positive difference indicates group has higher coprescribing intensity than those receiving opioids and benzodiazepines from different physicians)					
Same physician prescribing opioids and benzodiazepines	Before the guideline	3.87 (3.11 to 4.62) [b]	-3.25 (-3.55 to -2.96) [b]			3.01 (2.33 to 3.69) [b]	1.49 (1.16 to 1.82) [b]
	After the guideline	3.94 (3.20 to 4.69) [b]	-2.56 (-2.88 to -2.23) [b]			4.21 (3.66 to 4.76) [b]	2.07 (1.77 to 2.37) [b]
Unknown	Before the guideline	-3.71 (-5.91 to -1.51) [b]	-7.60 (-8.18 to -7.02) [b]			-2.17 (-3.46 to -0.89) [b]	-0.89 (-1.54 to -0.24) [b]
	After the guideline	-3.72 (-5.84 to -1.59) [b]	-7.44 (-8.06 to -6.83) [b]			-0.02 (-0.90 to 0.85)	-0.66 (-1.15 to -0.17) [b]

Adjusted percentages represent predictive margins from a zero-truncated negative binomial model that included patient age, sex, race/ethnicity, state of residence, and Elixhauser comorbidity flags calculated on a rolling 6 month basis. Separate models were specified for each patient population and episode type (commercial and Medicare Advantage; long-term and short-term). Standard errors in the model were adjusted for clustering on individual patient.
Absolute differences are in percentage points
[b] Difference statistically significantly different than 0 after controlling for family-wise error rate of 0.05 within table

© 2019 Jeffery MM et al. *JAMA Network Open.*

eTable 16. Extent of Coprescription by Race/Ethnicity: Adjusted Percent of Overlapping Opioids and BZDs in Month

Race/ethnicity	White	Black	Hispanic	Asian	Unknown/other
	Value (95% CI)	Value (95% CI)	Value (95% CI)	Value (95% CI)	Value (95% CI)
	**Long-term opioid use episodes**				
Study start	24.31 (23.62 to 25.01)	19.53 (18.01 to 21.04)	22.08 (20.04 to 24.12)	19.62 (14.42 to 24.82)	23.50 (19.40 to 27.61)
Just before guideline	26.33 (25.82 to 26.84)	19.47 (18.39 to 20.54)	24.76 (23.24 to 26.28)	19.93 (16.19 to 23.67)	25.84 (22.87 to 28.81)
Just after guideline	27.07 (26.57 to 27.57)	19.01 (18.00 to 20.01)	23.54 (22.12 to 24.96)	19.97 (16.50 to 23.43)	25.70 (23.20 to 28.20)
Study end	24.71 (24.19 to 25.22)	18.12 (17.12 to 19.11)	23.11 (21.56 to 24.67)	18.46 (14.35 to 22.57)	23.19 (21.30 to 25.09)
	**Short-term opioid use episodes**				
Study start	11.35 (11.26 to 11.44)	7.24 (7.07 to 7.42)	9.10 (8.90 to 9.31)	6.43 (6.08 to 6.77)	10.90 (10.43 to 11.38)
Just before guideline	11.81 (11.72 to 11.90)	8.08 (7.90 to 8.27)	10.15 (9.93 to 10.37)	6.67 (6.33 to 7.02)	11.36 (10.88 to 11.83)
Just after guideline	11.40 (11.31 to 11.49)	7.29 (7.12 to 7.45)	9.43 (9.21 to 9.64)	6.25 (5.88 to 6.62)	10.55 (10.18 to 10.92)
Study end	11.71 (11.61 to 11.81)	7.67 (7.49 to 7.85)	9.70 (9.47 to 9.93)	6.95 (6.51 to 7.39)	10.67 (10.38 to 10.97)

© 2019 Jeffery MM et al. *JAMA Network Open.*

eTable 17. Changes in Extent of Opioid and Benzodiazepine Coprescribing Associated With CDC Guideline Release, by Race/Ethnicity

Race/ethnicity	White	Black	Hispanic	Asian	Unknown/other
	Value (95% CI)	Value (95% CI)	Value (95% CI)	Value (95% CI)	Value (95% CI)
	**Long-term opioid use episodes**				
Change in level (percentage points)	0.74 (0.26 to 1.22)	-0.46 (-1.48 to 0.56)	-1.22 (-2.67 to 0.23)	0.04 (-3.61 to 3.69)	-0.14 (-2.97 to 2.69)
p-value (change in level=0)	0.003 [b]	0.377	0.100	0.982	0.924
pre-guideline release slope[a]	0.93 (0.50 to 1.36)	-0.03 (-0.96 to 0.90)	1.24 (-0.07 to 2.54)	0.14 (-3.11 to 3.40)	1.08 (-1.46 to 3.61)
post-guideline release slope[a]	-1.18 (-1.56 to -0.80)	-0.44 (-1.19 to 0.30)	-0.21 (-1.32 to 0.90)	-0.76 (-3.65 to 2.14)	-1.26 (-2.99 to 0.48)
p-value (change in slope=0)	<0.001 [b]	0.529	0.128	0.709	0.174
	**Short-term opioid use episodes**				
Change in level (percentage points)	-0.41 (-0.52 to -0.29)	-0.80 (-1.02 to -0.58)	-0.72 (-1.00 to -0.45)	-0.42 (-0.89 to 0.05)	-0.80 (-1.35 to -0.26)
p-value (change in level=0)	<0.001 [b]	<0.001 [b]	<0.001 [b]	0.079	0.004
pre-guideline release slope[a]	0.21 (0.15 to 0.28)	0.39 (0.26 to 0.52)	0.48 (0.33 to 0.64)	0.11 (-0.15 to 0.37)	0.21 (-0.14 to 0.56)
post-guideline release slope[a]	0.15 (0.07 to 0.23)	0.19 (0.05 to 0.33)	0.14 (-0.05 to 0.32)	0.35 (0.01 to 0.69)	0.06 (-0.22 to 0.34)
p-value (change in slope=0)	0.266	0.054	0.006	0.283	0.527

Adjusted rates of overlapping opioid and benzodiazepine fills represent predictive margins from a logit model that included patient age, sex, race/ethnicity, state of residence, and Elixhauser comorbidity flags calculated on a rolling 6 month basis. Adjusted rates of opioid days with benzodiazepines were specified with the same covariates, but were negative binomial models using the number of opioid days in the month as an exposure variable (i.e., the natural log of opioid days was included in the model with the coefficient constrained to be 1). Separate models were specified for each patient population (commercial, Medicare Advantage). Standard errors in the model were adjusted for clustering on individual patient. BZD=benzodiazepine

[a] Slopes given in units of percentage point change per 12 months

[b] Statistically significant after controlling for family-wise error rate of 0.05 within table

© 2019 Jeffery MM et al. *JAMA Network Open.*

eTable 18. Regression Results Secondary Analysis: Race/Ethnicity Short-term Episodes

Any overlap days Commercial and Medicare	Coef.	Robust Std. Err.	Z	P>z	[95 Conf.	Interval]
race/ethnicity by months before release						
White, non-Hispanic	0.002	0.000	6.360	0.000	0.001	0.002
Black	0.005	0.001	5.740	0.000	0.003	0.006
Hispanic, any race	0.005	0.001	6.070	0.000	0.003	0.006
Asian	0.002	0.002	0.860	0.390	-0.002	0.005
Unknown/other	0.002	0.002	1.170	0.242	-0.001	0.005
race/ethnicity by months since release						
White, non-Hispanic	-0.001	0.000	-1.110	0.269	-0.001	0.000
Black	-0.002	0.001	-1.870	0.062	-0.005	0.000
Hispanic, any race	-0.003	0.001	-2.730	0.006	-0.006	-0.001
Asian	0.003	0.003	1.070	0.286	-0.003	0.009
Unknown/other	-0.001	0.002	-0.610	0.541	-0.005	0.003
state						
AL	0.041	0.097	0.430	0.670	-0.149	0.232
AR	0.125	0.098	1.280	0.199	-0.066	0.317
AZ	-0.115	0.097	-1.190	0.234	-0.305	0.075
CA	-0.103	0.097	-1.060	0.289	-0.292	0.087
CO	-0.066	0.097	-0.690	0.493	-0.256	0.123
CT	-0.076	0.098	-0.780	0.437	-0.268	0.116
DC	-0.358	0.105	-3.420	0.001	-0.564	-0.153
DE	-0.163	0.119	-1.370	0.169	-0.396	0.070
FL	0.212	0.096	2.190	0.028	0.023	0.401
GA	-0.066	0.097	-0.680	0.497	-0.255	0.124
HI	-0.474	0.108	-4.390	0.000	-0.686	-0.262
IA	-0.274	0.098	-2.800	0.005	-0.466	-0.083
ID	-0.471	0.102	-4.620	0.000	-0.670	-0.271
IL	-0.180	0.097	-1.860	0.063	-0.369	0.010
IN	-0.327	0.097	-3.360	0.001	-0.517	-0.136
KS	-0.219	0.099	-2.210	0.027	-0.412	-0.025
KY	-0.333	0.099	-3.350	0.001	-0.528	-0.138
LA	0.199	0.097	2.050	0.040	0.009	0.389
MA	-0.111	0.098	-1.130	0.260	-0.303	0.082
MD	-0.228	0.097	-2.340	0.020	-0.418	-0.037
ME	-0.268	0.107	-2.510	0.012	-0.477	-0.058
MI	-0.035	0.098	-0.360	0.722	-0.227	0.157
MN	-0.677	0.097	-6.960	0.000	-0.867	-0.486
MO	-0.060	0.097	-0.620	0.534	-0.250	0.129
MS	-0.135	0.098	-1.370	0.169	-0.328	0.058
MT	-0.402	0.120	-3.350	0.001	-0.636	-0.167

© 2019 Jeffery MM et al. *JAMA Network Open.*

Any overlap days Commercial and Medicare	Coef.	Robust Std. Err.	Z	P>z	[95 Conf.	Interval]
NC	0.094	0.097	0.980	0.329	-0.095	0.284
ND	-0.500	0.108	-4.650	0.000	-0.711	-0.289
NE	-0.260	0.098	-2.650	0.008	-0.452	-0.068
NH	0.043	0.104	0.410	0.680	-0.161	0.247
NJ	-0.061	0.097	-0.620	0.532	-0.251	0.130
NM	-0.191	0.100	-1.910	0.056	-0.386	0.005
NV	0.058	0.100	0.580	0.559	-0.138	0.254
NY	-0.382	0.097	-3.930	0.000	-0.572	-0.192
OH	-0.311	0.097	-3.210	0.001	-0.500	-0.121
OK	-0.219	0.098	-2.230	0.026	-0.411	-0.027
OR	-0.443	0.098	-4.500	0.000	-0.636	-0.250
PA	-0.130	0.098	-1.330	0.184	-0.321	0.062
RI	-0.004	0.099	-0.040	0.970	-0.198	0.191
SC	0.129	0.097	1.330	0.184	-0.062	0.320
SD	-0.503	0.113	-4.450	0.000	-0.725	-0.282
TN	-0.064	0.097	-0.650	0.513	-0.254	0.127
TX	-0.142	0.096	-1.480	0.139	-0.331	0.046
UT	-0.245	0.097	-2.510	0.012	-0.436	-0.054
VA	-0.035	0.097	-0.360	0.721	-0.225	0.156
VT	-0.356	0.120	-2.970	0.003	-0.590	-0.121
WA	-0.415	0.098	-4.250	0.000	-0.607	-0.224
WI	-0.291	0.097	-3.010	0.003	-0.481	-0.101
WV	-0.129	0.109	-1.190	0.235	-0.343	0.084
WY	-0.491	0.115	-4.270	0.000	-0.716	-0.265
Unknown/other	-0.124	0.101	-1.220	0.221	-0.323	0.075
post-guideline (binary) by race/ethnicity						
0 by B	-2.395	0.575	-4.170	0.000	-3.522	-1.269
0 by H	-2.190	0.555	-3.950	0.000	-3.278	-1.103
0 by A	-0.505	1.237	-0.410	0.683	-2.930	1.919
0 by U	-0.066	1.052	-0.060	0.950	-2.127	1.996
1 by W	-0.041	0.006	-6.980	0.000	-0.053	-0.030
1 by B	-2.511	0.583	-4.310	0.000	-3.654	-1.369
1 by H	-2.275	0.563	-4.040	0.000	-3.379	-1.172
1 by A	-0.577	1.258	-0.460	0.647	-3.043	1.889
1 by U	-0.151	1.070	-0.140	0.888	-2.248	1.945
age	0.008	0.000	50.430	0.000	0.007	0.008
female	0.416	0.004	105.190	0.000	0.408	0.424
beneficiary type						
Medicare	0.181	0.006	28.140	0.000	0.168	0.194

© 2019 Jeffery MM et al. *JAMA Network Open.*

Any overlap days Commercial and Medicare	Coef.	Robust Std. Err.	Z	P>z	[95 Conf.	Interval]
elix_chf	-0.024	0.011	-2.160	0.031	-0.045	-0.002
elix_arrhythmia	-0.035	0.008	-4.260	0.000	-0.051	-0.019
elix_valvular	-0.070	0.013	-5.520	0.000	-0.095	-0.045
elix_pulm_circ	0.031	0.018	1.690	0.091	-0.005	0.066
elix_periph_vasc	-0.092	0.011	-8.090	0.000	-0.115	-0.070
elix_htn_uncomp	0.054	0.005	11.950	0.000	0.045	0.063
elix_htn_comp	-0.044	0.013	-3.410	0.001	-0.069	-0.019
elix_oth_neuro	0.349	0.011	31.130	0.000	0.327	0.370
elix_chron_pulm	0.337	0.007	49.420	0.000	0.324	0.351
elix_pep_ulcer	0.052	0.034	1.530	0.125	-0.014	0.119
elix_dm_wo_cc	-0.076	0.007	-11.580	0.000	-0.089	-0.063
elix_dm_w_cc	-0.131	0.009	-14.810	0.000	-0.148	-0.113
elix_paralysis	0.027	0.028	0.990	0.322	-0.027	0.081
elix_renal	-0.158	0.011	-13.750	0.000	-0.180	-0.135
elix_solid_tumor_wo_mets	-0.024	0.014	-1.660	0.097	-0.052	0.004
elix_liver	0.121	0.016	7.770	0.000	0.090	0.151
elix_met_cancer	-0.041	0.057	-0.720	0.473	-0.153	0.071
elix_hiv_aids	0.496	0.039	12.750	0.000	0.419	0.572
elix_rheum_arth_coll	0.057	0.012	4.880	0.000	0.034	0.080
elix_hypothyroid	0.129	0.008	16.770	0.000	0.114	0.145
elix_lymphoma	0.047	0.048	0.990	0.324	-0.047	0.141
elix_coagulopathy	-0.052	0.018	-2.890	0.004	-0.088	-0.017
elix_obesity	-0.095	0.009	-10.500	0.000	-0.113	-0.077
elix_weight_loss	0.068	0.018	3.850	0.000	0.033	0.102
elix_fluid_electrolyte	0.064	0.010	6.760	0.000	0.046	0.083
elix_blood_loss_anemia	-0.133	0.028	-4.720	0.000	-0.188	-0.078
elix_deficiency_anemia	0.072	0.015	4.860	0.000	0.043	0.100
elix_alcohol	0.160	0.019	8.320	0.000	0.122	0.198
elix_drug_abuse	0.555	0.019	29.990	0.000	0.519	0.591
elix_psychosis	0.607	0.018	33.130	0.000	0.571	0.643
elix_depression	1.178	0.006	197.920	0.000	1.166	1.189
less than 6 months prior insurance coverage	-0.007	0.004	-1.550	0.122	-0.015	0.002
_cons	-3.988	0.211	-18.900	0.000	-4.402	-3.575
Observations: 9,741,154 person-months contributed by 4,823,962 individuals						

eTable 19. Regression Results Secondary Analysis: Race/Ethnicity Long-term Episodes

Any overlap days Commercial and Medicare long-term episodes	Coef.	Robust Std. Err.	Z	P>z	[95 Conf.	Interval]
race/ethnicity by months before release						
White, non-Hispanic	0.004	0.001	4.210	0.000	0.002	0.006

© 2019 Jeffery MM et al. *JAMA Network Open.*

Any overlap days Commercial and Medicare long-term episodes	Coef.	Robust Std. Err.	Z	P>z	[95 Conf.	Interval]
Black	0.000	0.003	-0.060	0.953	-0.005	0.005
Hispanic, any race	0.006	0.003	1.840	0.066	0.000	0.012
Asian	0.001	0.009	0.080	0.933	-0.017	0.019
Unknown/other	0.005	0.006	0.830	0.409	-0.007	0.017
race/ethnicity by months since release						
White, non-Hispanic	-0.010	0.001	-6.610	0.000	-0.013	-0.007
Black	-0.002	0.004	-0.650	0.518	-0.010	0.005
Hispanic, any race	-0.007	0.005	-1.520	0.130	-0.016	0.002
Asian	-0.005	0.013	-0.380	0.707	-0.031	0.021
Unknown/other	-0.011	0.008	-1.360	0.174	-0.027	0.005
state						
AL	0.919	0.419	2.190	0.028	0.097	1.740
AR	0.823	0.421	1.960	0.050	-0.001	1.647
AZ	0.446	0.419	1.060	0.288	-0.376	1.268
CA	0.719	0.419	1.710	0.086	-0.103	1.540
CO	0.528	0.420	1.260	0.209	-0.295	1.350
CT	0.730	0.424	1.720	0.085	-0.100	1.560
DC	0.416	0.463	0.900	0.368	-0.491	1.324
DE	0.926	0.470	1.970	0.049	0.005	1.847
FL	1.009	0.417	2.420	0.016	0.191	1.828
GA	0.733	0.418	1.750	0.080	-0.087	1.552
HI	0.654	0.444	1.470	0.141	-0.217	1.525
IA	0.584	0.423	1.380	0.167	-0.245	1.414
ID	0.503	0.432	1.170	0.244	-0.343	1.349
IL	0.704	0.419	1.680	0.093	-0.117	1.525
IN	0.526	0.419	1.260	0.209	-0.295	1.347
KS	0.586	0.426	1.380	0.169	-0.249	1.420
KY	0.578	0.426	1.360	0.175	-0.258	1.414
LA	0.974	0.420	2.320	0.020	0.150	1.797
MA	0.713	0.426	1.670	0.094	-0.122	1.547
MD	0.428	0.423	1.010	0.312	-0.402	1.257
ME	0.544	0.450	1.210	0.227	-0.338	1.427
MI	0.766	0.423	1.810	0.070	-0.063	1.595
MN	0.213	0.421	0.510	0.612	-0.611	1.038
MO	0.848	0.418	2.030	0.043	0.028	1.669
MS	0.716	0.424	1.690	0.091	-0.115	1.546
MT	0.669	0.496	1.350	0.177	-0.302	1.640
NC	0.940	0.418	2.250	0.024	0.121	1.759
ND	0.433	0.454	0.950	0.340	-0.457	1.323
NE	0.509	0.424	1.200	0.230	-0.322	1.341

© 2019 Jeffery MM et al. *JAMA Network Open.*

Any overlap days Commercial and Medicare long-term episodes	Coef.	Robust Std. Err.	Z	P>z	[95 Conf.	Interval]
NH	0.666	0.445	1.500	0.134	-0.206	1.538
NJ	0.860	0.421	2.040	0.041	0.034	1.685
NM	0.411	0.425	0.970	0.334	-0.423	1.244
NV	0.729	0.432	1.690	0.091	-0.117	1.575
NY	0.468	0.419	1.120	0.264	-0.353	1.289
OH	0.541	0.418	1.290	0.196	-0.279	1.361
OK	0.543	0.422	1.290	0.198	-0.284	1.371
OR	0.123	0.426	0.290	0.773	-0.712	0.958
PA	0.640	0.422	1.510	0.130	-0.188	1.467
RI	0.549	0.426	1.290	0.197	-0.286	1.384
SC	0.889	0.420	2.120	0.034	0.067	1.711
SD	0.600	0.455	1.320	0.187	-0.292	1.491
TN	0.749	0.419	1.790	0.074	-0.073	1.571
TX	0.682	0.418	1.630	0.102	-0.137	1.501
UT	0.658	0.420	1.570	0.117	-0.166	1.483
VA	0.905	0.420	2.150	0.031	0.081	1.728
VT	0.200	0.482	0.410	0.678	-0.745	1.146
WA	0.276	0.422	0.650	0.513	-0.551	1.102
WI	0.516	0.419	1.230	0.218	-0.305	1.336
WV	0.416	0.465	0.900	0.371	-0.495	1.327
WY	0.186	0.498	0.370	0.709	-0.791	1.163
Unknown/other	0.762	0.429	1.780	0.076	-0.079	1.604
post-guideline (binary) by race/ethnicity						
0 by B	2.605	1.878	1.390	0.165	-1.075	6.285
0 by H	-1.245	2.286	-0.540	0.586	-5.725	3.235
0 by A	2.008	6.119	0.330	0.743	-9.985	14.001
0 by U	-0.546	4.155	-0.130	0.895	-8.689	7.597
1 by W	0.040	0.013	3.000	0.003	0.014	0.066
1 by B	2.574	1.892	1.360	0.174	-1.134	6.282
1 by H	-1.315	2.303	-0.570	0.568	-5.829	3.199
1 by A	2.011	6.177	0.330	0.745	-10.095	14.117
1 by U	-0.553	4.191	-0.130	0.895	-8.768	7.662
age	-0.018	0.001	-28.400	0.000	-0.019	-0.016
female	0.428	0.015	28.660	0.000	0.399	0.458
beneficiary type						
Medicare	0.368	0.021	17.850	0.000	0.328	0.409
elix_chf	-0.016	0.028	-0.560	0.574	-0.070	0.039
elix_arrhythmia	0.001	0.023	0.030	0.977	-0.045	0.046

© 2019 Jeffery MM et al. *JAMA Network Open.*

Any overlap days Commercial and Medicare long-term episodes	Coef.	Robust Std. Err.	Z	P>z	[95 Conf.	Interval]
elix_valvular	-0.019	0.036	-0.540	0.592	-0.089	0.051
elix_pulm_circ	-0.074	0.046	-1.600	0.109	-0.165	0.017
elix_periph_vasc	-0.145	0.027	-5.310	0.000	-0.199	-0.092
elix_htn_uncomp	0.047	0.012	3.800	0.000	0.023	0.072
elix_htn_comp	-0.011	0.033	-0.330	0.739	-0.075	0.053
elix_oth_neuro	0.282	0.029	9.830	0.000	0.226	0.339
elix_chron_pulm	0.315	0.018	17.330	0.000	0.279	0.351
elix_pep_ulcer	0.008	0.069	0.120	0.903	-0.126	0.143
elix_dm_wo_cc	-0.108	0.018	-5.890	0.000	-0.144	-0.072
elix_dm_w_cc	-0.220	0.023	-9.620	0.000	-0.265	-0.175
elix_paralysis	0.013	0.061	0.220	0.825	-0.106	0.133
elix_renal	-0.170	0.029	-5.780	0.000	-0.227	-0.112
elix_solid_tumor_wo_mets	-0.070	0.078	-0.900	0.370	-0.223	0.083
elix_liver	0.128	0.038	3.400	0.001	0.054	0.201
elix_met_cancer	-0.313	0.248	-1.260	0.206	-0.798	0.172
elix_hiv_aids	0.600	0.127	4.710	0.000	0.351	0.850
elix_rheum_arth_coll	-0.162	0.025	-6.530	0.000	-0.210	-0.113
elix_hypothyroid	0.120	0.022	5.320	0.000	0.076	0.164
elix_lymphoma	0.341	0.201	1.690	0.091	-0.054	0.736
elix_coagulopathy	0.005	0.047	0.100	0.924	-0.088	0.097
elix_obesity	-0.167	0.024	-6.900	0.000	-0.214	-0.119
elix_weight_loss	-0.015	0.036	-0.400	0.686	-0.086	0.056
elix_fluid_electrolyte	0.123	0.022	5.590	0.000	0.080	0.166
elix_blood_loss_anemia	-0.082	0.067	-1.210	0.225	-0.214	0.050
elix_deficiency_anemia	0.023	0.037	0.610	0.543	-0.050	0.095
elix_alcohol	0.119	0.050	2.390	0.017	0.021	0.216
elix_drug_abuse	0.187	0.029	6.390	0.000	0.130	0.245
elix_psychosis	0.454	0.044	10.300	0.000	0.367	0.540
elix_depression	0.854	0.016	52.330	0.000	0.822	0.886
less than 6 months prior insurance coverage	-0.085	0.017	-4.980	0.000	-0.119	-0.052
_cons	-4.201	0.798	-5.260	0.000	-5.765	-2.636
Observations: 1,624,004 person-months contributed by 128,410 individuals						

© 2019 Jeffery MM et al. *JAMA Network Open.*

eTable 20. Extent of Coprescription by Opioid Dose: Adjusted Percent With Overlapping Opioids and Benzodiazepines in Month

Opioid dose	Less than 20 MME per day	20 to 50 MME	50 to 90 MME	90 to 120 MME	120 or more MME
	Value (95% CI)	Value (95% CI)	Value (95% CI)	Value (95% CI)	Value (95% CI)
	**Long-term opioid use episodes**				
Study start	19.74 (18.86 to 20.61)	23.43 (22.63 to 24.23)	28.94 (27.46 to 30.43)	32.06 (29.26 to 34.86)	36.09 (33.26 to 38.91)
Just before guideline	20.30 (19.65 to 20.96)	24.63 (24.04 to 25.22)	31.22 (30.11 to 32.33)	33.43 (31.43 to 35.43)	37.80 (35.94 to 39.66)
Just after guideline	20.79 (20.14 to 21.43)	25.11 (24.53 to 25.69)	31.66 (30.55 to 32.78)	34.19 (32.18 to 36.19)	38.42 (36.59 to 40.26)
Study end	19.20 (18.55 to 19.86)	23.28 (22.68 to 23.89)	29.25 (28.08 to 30.41)	30.19 (28.19 to 32.20)	35.90 (33.83 to 37.96)
	**Short-term opioid use episodes**				
Study start	10.65 (10.48 to 10.81)	9.81 (9.72 to 9.90)	11.02 (10.87 to 11.18)	15.32 (14.82 to 15.82)	17.87 (17.16 to 18.59)
Just before guideline	11.22 (11.07 to 11.37)	10.41 (10.32 to 10.50)	11.47 (11.31 to 11.62)	14.52 (14.06 to 14.98)	19.69 (18.94 to 20.45)
Just after guideline	10.69 (10.54 to 10.84)	9.98 (9.89 to 10.07)	10.75 (10.58 to 10.92)	14.34 (13.84 to 14.85)	19.07 (18.24 to 19.89)
Study end	10.75 (10.60 to 10.89)	10.20 (10.10 to 10.29)	11.91 (11.71 to 12.11)	14.46 (13.93 to 14.99)	19.60 (18.74 to 20.46)

eTable 21. Changes in Extent of Opioid and Benzodiazepine Coprescribing Associated With CDC Guideline Release, by Opioid Dose

Opioid dose	Less than 20 MME per day	20 to 50 MME	50 to 90 MME	90 to 120 MME	120 or more MME
	Value (95% CI)	Value (95% CI)	Value (95% CI)	Value (95% CI)	Value (95% CI)
	**Long-term opioid use episodes**				

© 2019 Jeffery MM et al. *JAMA Network Open.*

Opioid dose	Less than 20 MME per day	20 to 50 MME	50 to 90 MME	90 to 120 MME	120 or more MME
	Value (95% CI)	Value (95% CI)	Value (95% CI)	Value (95% CI)	Value (95% CI)
Change in level (percentage points)	0.48 (-0.19 to 1.16)	0.48 (-0.11 to 1.07)	0.44 (-0.72 to 1.60)	0.76 (-1.39 to 2.90)	0.62 (-1.14 to 2.38)
p-value (change in level=0)	0.162	0.108	0.460	0.490	0.489
pre-guideline release slope[a]	0.26 (-0.31 to 0.83)	0.55 (0.05 to 1.06)	1.05 (0.10 to 2.01)	0.63 (-1.17 to 2.43)	0.79 (-0.91 to 2.49)
post-guideline release slope[a]	-0.79 (-1.29 to -0.29)	-0.91 (-1.37 to -0.46)	-1.21 (-2.09 to -0.33)	-2.00 (-3.56 to -0.43)	-1.26 (-2.72 to 0.19)
p-value (change in slope=0)	0.012	0.000 [b]	0.002 [b]	0.046	0.095
	**Short-term opioid use episodes**				
Change in level (percentage points)	-0.53 (-0.72 to -0.34)	-0.43 (-0.55 to -0.32)	-0.71 (-0.94 to -0.49)	-0.17 (-0.84 to 0.49)	-0.62 (-1.69 to 0.45)
p-value (change in level=0)	0.000 [b]	0.000 [b]	0.000 [b]	0.604	0.253
pre-guideline release slope[a]	0.27 (0.15 to 0.38)	0.28 (0.21 to 0.34)	0.20 (0.08 to 0.33)	-0.37 (-0.74 to 0.00)	0.84 (0.27 to 1.41)
post-guideline release slope[a]	0.03 (-0.10 to 0.15)	0.11 (0.03 to 0.19)	0.58 (0.42 to 0.74)	0.06 (-0.39 to 0.50)	0.27 (-0.46 to 0.99)
p-value (change in slope=0)	0.008	0.002 [b]	0.000 [b]	0.154	0.233

Adjusted rates of overlapping opioid and benzodiazepine fills represent predictive margins from a logit model that included patient age, sex, race/ethnicity, state of residence, and Elixhauser comorbidity flags calculated on a rolling 6 month basis. eparate models were specified for each patient population and episode type (commercial and Medicare Advantage; long-term and short-term). Standard errors in the model were adjusted for clustering on individual patient.

[a] Slopes given in units of percentage point change per 12 months

[b] Statistically significant after controlling for family-wise error rate of 0.05 within table

© 2019 Jeffery MM et al. *JAMA Network Open.*

eTable 22. Regression Results Sensitivity Analysis: Episodes Starting Before vs After Guideline Release

Any overlap in the first 90 days of the episode Commercial and Medicare	Coef.	Std. Err.	z	P>z	[95 Conf.	Interval]
beneficiary type by months before release						
Commercial	-0.002	0.002	-0.990	0.321	-0.005	0.002
Medicare	0.001	0.002	0.550	0.584	-0.002	0.004
beneficiary type by months since release						
Commercial	-0.004	0.003	-1.260	0.207	-0.011	0.002
Medicare	-0.007	0.003	-2.410	0.016	-0.012	-0.001
state						
AL	0.497	0.658	0.750	0.451	-0.794	1.787
AR	0.424	0.659	0.640	0.520	-0.868	1.717
AZ	0.194	0.659	0.290	0.768	-1.097	1.485
CA	0.356	0.658	0.540	0.588	-0.934	1.647
CO	0.362	0.659	0.550	0.583	-0.930	1.653
CT	0.425	0.661	0.640	0.520	-0.870	1.720
DC	0.157	0.682	0.230	0.818	-1.179	1.493
DE	0.823	0.693	1.190	0.235	-0.535	2.181
FL	0.644	0.657	0.980	0.327	-0.645	1.932
GA	0.387	0.658	0.590	0.556	-0.902	1.676
HI	0.089	0.678	0.130	0.896	-1.241	1.418
IA	0.222	0.661	0.340	0.736	-1.072	1.517
ID	0.059	0.665	0.090	0.930	-1.244	1.361
IL	0.299	0.658	0.450	0.650	-0.991	1.588
IN	0.104	0.658	0.160	0.875	-1.187	1.394
KS	0.162	0.664	0.240	0.807	-1.138	1.463
KY	0.106	0.663	0.160	0.873	-1.194	1.407
LA	0.528	0.659	0.800	0.424	-0.765	1.820
MA	0.397	0.663	0.600	0.550	-0.902	1.696
MD	0.137	0.661	0.210	0.835	-1.158	1.433
ME	-0.031	0.680	-0.050	0.964	-1.364	1.302
MI	0.414	0.661	0.630	0.532	-0.883	1.710
MN	-0.064	0.659	-0.100	0.922	-1.357	1.228
MO	0.453	0.658	0.690	0.491	-0.837	1.742
MS	0.220	0.663	0.330	0.740	-1.079	1.519
MT	0.200	0.728	0.280	0.783	-1.227	1.627
NC	0.566	0.658	0.860	0.389	-0.723	1.855
ND	0.295	0.681	0.430	0.665	-1.039	1.629
NE	0.320	0.661	0.480	0.628	-0.976	1.616
NH	0.359	0.677	0.530	0.596	-0.968	1.685

© 2019 Jeffery MM et al. *JAMA Network Open.*

Any overlap in the first 90 days of the episode Commercial and Medicare	Coef.	Std. Err.	z	P>z	[95 Conf.	Interval]
NJ	0.496	0.659	0.750	0.452	-0.796	1.788
NM	0.019	0.664	0.030	0.977	-1.281	1.320
NV	0.246	0.666	0.370	0.713	-1.061	1.552
NY	0.065	0.658	0.100	0.921	-1.225	1.355
OH	0.131	0.658	0.200	0.842	-1.158	1.421
OK	0.059	0.661	0.090	0.929	-1.238	1.355
OR	0.113	0.662	0.170	0.864	-1.185	1.412
PA	0.215	0.660	0.330	0.745	-1.079	1.509
RI	0.226	0.663	0.340	0.734	-1.074	1.525
SC	0.577	0.659	0.880	0.381	-0.714	1.868
SD	0.039	0.688	0.060	0.954	-1.308	1.387
TN	0.334	0.659	0.510	0.612	-0.956	1.625
TX	0.307	0.658	0.470	0.641	-0.982	1.595
UT	0.324	0.659	0.490	0.622	-0.967	1.616
VA	0.436	0.659	0.660	0.508	-0.856	1.728
VT	-0.072	0.695	-0.100	0.918	-1.433	1.290
WA	0.092	0.660	0.140	0.889	-1.202	1.385
WI	0.217	0.658	0.330	0.742	-1.073	1.507
WV	0.219	0.683	0.320	0.749	-1.120	1.558
WY	-0.253	0.709	-0.360	0.722	-1.642	1.137
Unknown/other	0.466	0.681	0.680	0.494	-0.869	1.802
race/ethnicity						
B	-0.370	0.022	-16.860	0.000	-0.412	-0.327
H	-0.097	0.027	-3.670	0.000	-0.150	-0.045
A	-0.366	0.070	-5.240	0.000	-0.503	-0.229
U	-0.001	0.037	-0.020	0.987	-0.074	0.073
elix_chf	0.001	0.038	0.040	0.970	-0.073	0.076
elix_arrhythmia	0.001	0.032	0.040	0.965	-0.060	0.063
elix_valvular	-0.034	0.051	-0.660	0.510	-0.134	0.067
elix_pulm_circ	0.073	0.065	1.130	0.261	-0.054	0.200
elix_periph_vasc	-0.146	0.041	-3.600	0.000	-0.226	-0.067
elix_htn_uncomp	0.042	0.018	2.370	0.018	0.007	0.077
elix_htn_comp	-0.039	0.049	-0.790	0.430	-0.135	0.058
elix_oth_neuro	0.263	0.037	7.100	0.000	0.190	0.335
elix_chron_pulm	0.297	0.025	11.870	0.000	0.248	0.346
elix_pep_ulcer	-0.035	0.116	-0.300	0.763	-0.263	0.193
elix_dm_wo_cc	-0.088	0.024	-3.650	0.000	-0.135	-0.041
elix_dm_w_cc	-0.274	0.032	-8.470	0.000	-0.338	-0.211
elix_paralysis	-0.072	0.077	-0.940	0.350	-0.223	0.079
elix_renal	-0.129	0.041	-3.160	0.002	-0.210	-0.049

© 2019 Jeffery MM et al. *JAMA Network Open.*

Any overlap in the first 90 days of the episode Commercial and Medicare	Coef.	Std. Err.	z	P>z	[95 Conf.	Interval]
elix_solid_tumor_wo_mets	-0.020	0.104	-0.190	0.846	-0.223	0.183
elix_liver	0.089	0.058	1.540	0.124	-0.024	0.203
elix_met_cancer	0.278	0.353	0.790	0.432	-0.415	0.971
elix_hiv_aids	0.272	0.158	1.720	0.085	-0.037	0.582
elix_rheum_arth_coll	-0.187	0.035	-5.330	0.000	-0.256	-0.118
elix_hypothyroid	0.147	0.032	4.610	0.000	0.085	0.210
elix_lymphoma	0.551	0.297	1.850	0.064	-0.031	1.134
elix_coagulopathy	-0.007	0.070	-0.090	0.925	-0.143	0.130
elix_obesity	-0.207	0.039	-5.260	0.000	-0.284	-0.130
elix_weight_loss	0.018	0.059	0.310	0.756	-0.098	0.135
elix_fluid_electrolyte	0.133	0.036	3.650	0.000	0.062	0.205
elix_blood_loss_anemia	0.051	0.108	0.470	0.639	-0.161	0.262
elix_deficiency_anemia	-0.044	0.056	-0.780	0.435	-0.155	0.067
elix_alcohol	0.073	0.070	1.050	0.293	-0.063	0.210
elix_drug_abuse	0.292	0.053	5.500	0.000	0.188	0.396
elix_psychosis	0.407	0.055	7.400	0.000	0.299	0.514
elix_depression	0.851	0.024	35.580	0.000	0.805	0.898
less than 6 months prior insurance coverage	-0.001	0.020	-0.070	0.943	-0.041	0.038
post-guideline (binary) by beneficiary type						
0 by Medicare	-1.464	1.599	-0.920	0.360	-4.598	1.670
1 by com	0.091	0.043	2.140	0.032	0.008	0.175
1 by Medicare	-1.459	1.614	-0.900	0.366	-4.622	1.704
female	0.442	0.015	30.270	0.000	0.414	0.471
age	-0.018	0.001	-28.140	0.000	-0.019	-0.017
_cons	0.489	1.334	0.370	0.714	-2.125	3.104
Observations: 109,048 individuals/episodes						

eTable 23. Adjusted Coprescribing Extent Estimates and Trends in Long-term Opioid Use Episodes Starting Before vs After the Guidelines Were Released

	Com		MA
	Proportion of long-term opioid use episodes with opioid/benzodiazepine coprescribing in the first 90 days of the episode		
Study start	28.60% (27.58% to 29.62%)		29.09% (28.11% to 30.07%)
Just before guideline	27.61% (26.60% to 28.62%)		29.58% (28.61% to 30.54%)
Just after guideline	29.16% (27.86% to 30.45%)		29.63% (28.64% to 30.63%)
Study end	26.95% (25.26% to 28.64%)		27.06% (25.88% to 28.23%)

© 2019 Jeffery MM et al. *JAMA Network Open.*

	Com		MA
Change in level (percentage points)	1.55% (-0.09 to 3.19)		0.06% (-1.34% to 1.45%)
p-value (change in level=0)	0.0642		0.9378
pre-guideline release slope[a]	-0.46% (-1.26% to 0.34%)		0.22% (-0.54% to 0.99%)
post-guideline release slope[a]	-1.11% (-2.45% to 0.24%)		-1.29% (-2.26% to -0.32%)
p-value (change in slope=0)	0.415		0.0164

Adjusted rates of having overlapping opioid and benzodiazepine fills in the first 90 days of an incident long-term opioid use episode represent predictive margins from a logit model that included patient age, sex, race/ethnicity, state of residence, and Elixhauser comorbidity flags calculated on a rolling 6 month basis. The unit of observation was a long-term opioid use episode; episodes were included in the month in which they started. Separate models were specified for each patient population (commercial, Medicare Advantage). Standard errors in the model were adjusted for clustering on individual patient.

[a] Slopes given in units of percentage point change per 12 months

© 2019 Jeffery MM et al. *JAMA Network Open.*

eFigure 1. Cohort Flow Chart Describing Construction of the Study Sample

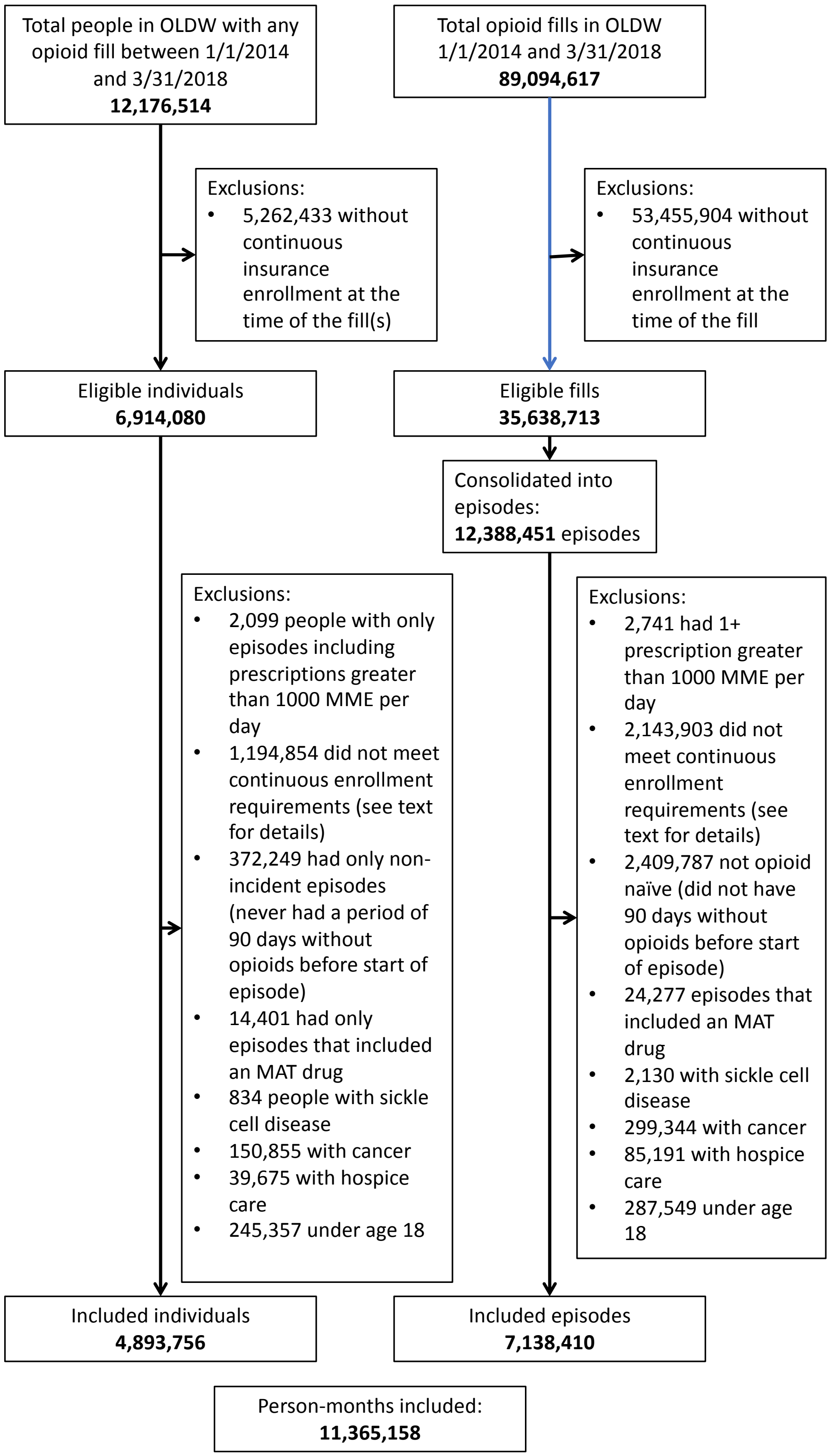

© 2019 Jeffery MM et al. *JAMA Network Open.*

eFigure 2. Race/Ethnicity: Coprescribing Extent

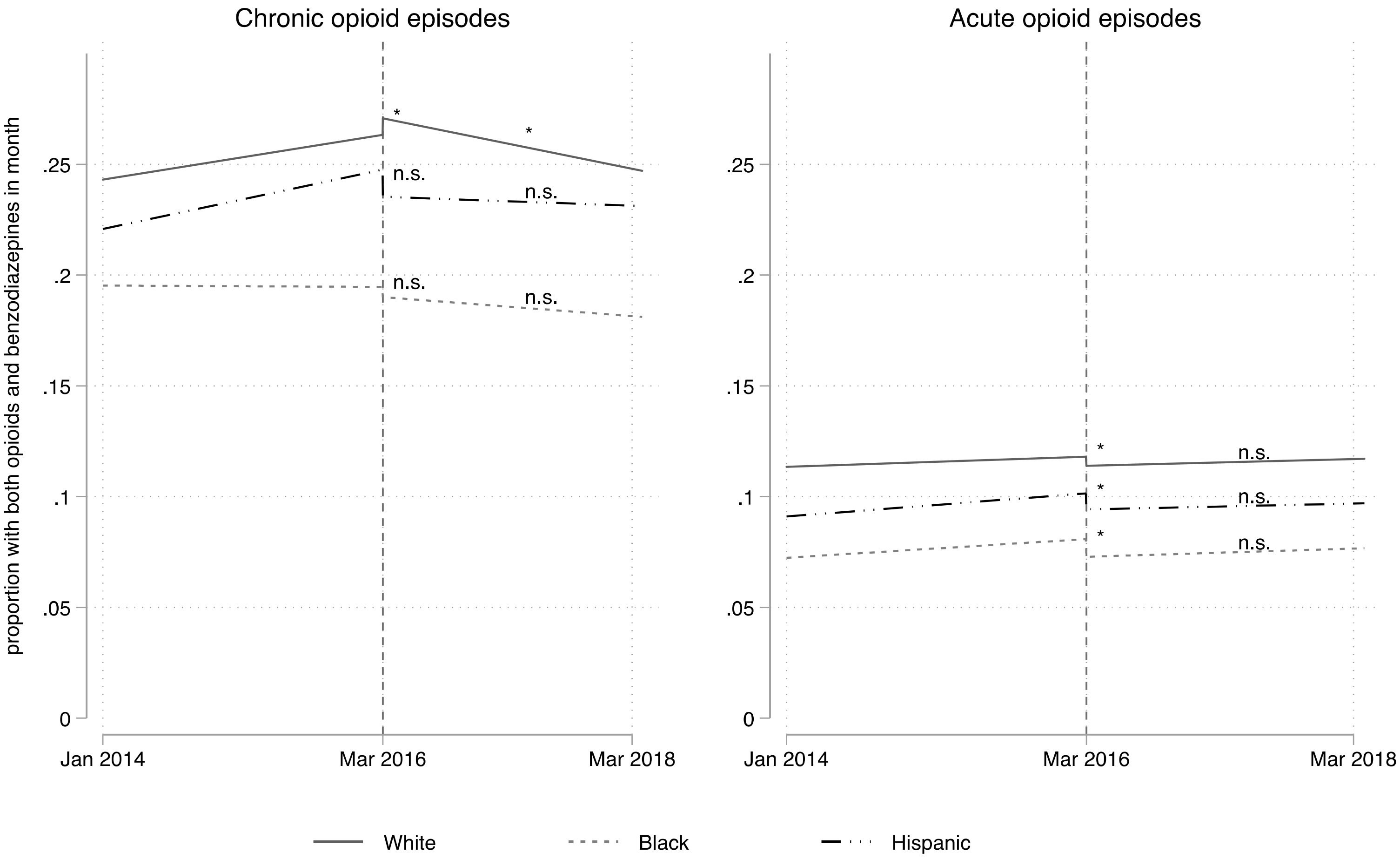

n.s. not statistically significant with family-wise error rate 0.05 across analyses in figure; * statistically significant at p=0.05. Statistical significance for change in level indicated next to line demarking release of opioid guidelines. Statistical significance for change in trend (line slope) indicated to the right of the statistical significance of the change in level. The plotplain package was used to display the figure.

© 2019 Jeffery MM et al. *JAMA Network Open.*

eFigure 3. Coprescribing Extent by Opioid Dose

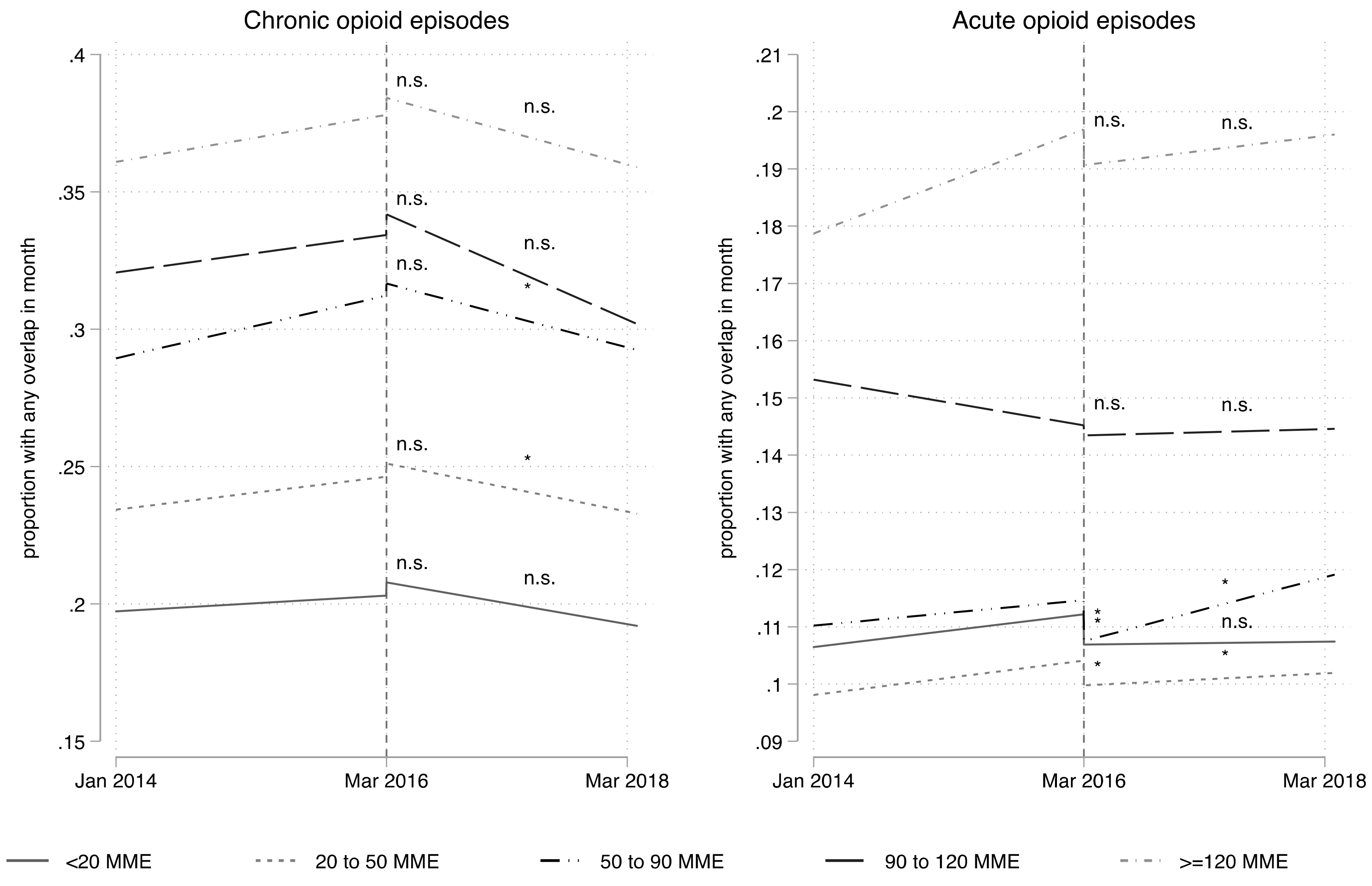

© 2019 Jeffery MM et al. *JAMA Network Open.*

n.s. not statistically significant with family-wise error rate 0.05 across analyses in figure; * statistically significant at p=0.05. Statistical significance for change in level indicated next to line demarking release of opioid guidelines. Statistical significance for change in trend (line slope) indicated to the right of the statistical significance of the change in level. The plotplain package was used to display the figure.

© 2019 Jeffery MM et al. *JAMA Network Open.*

eReferences

1. National Center for Injury Prevention and Control. CDC compilation of opioid analgesic formulations with morphine milligram equivalent conversion factors, 2015 version. 2015; http://www.pdmpassist.org/pdf/BJA_performance_measure_aid_MME_conversion.pdf.
2. Quan H, Sundararajan V, Halfon P, et al. Coding algorithms for defining comorbidities in ICD-9-CM and ICD-10 administrative data. *Medical care.* 2005;43(11):1130-1139.
3. Holm S. A Simple Sequentially Rejective Multiple Test Procedure. *Scandinavian Journal of Statistics.* 1979;6(2).
4. StataCorp. Stata/MP: Release 15. College Station, TX: StataCorp LLC; 2017.
5. Snyder AB, Lane PA, Zhou M, Paulukonis ST, Hulihan MM. The accuracy of hospital ICD-9-CM codes for determining Sickle Cell Disease genotype. *Journal of rare diseases research & treatment.* 2017;2(4):39-45.

© 2019 Jeffery MM et al. *JAMA Network Open.*
